# Towards a synthesis of the Caribbean biogeography of terrestrial arthropods

**DOI:** 10.1186/s12862-019-1576-z

**Published:** 2020-01-24

**Authors:** Sarah C. Crews, Lauren A. Esposito

**Affiliations:** California Academy of Sciences, Institute for Biodiversity Science and Sustainability, 55 Music Concourse Drive, San Francisco, CA 94118 USA

**Keywords:** Biogeography, Insects, Arachnids, BioGeoBEARS, Isthmus of Panama, Central American seaway, Islands, Probabilistic inference

## Abstract

**Background:**

The immense geologic and ecological complexity of the Caribbean has created a natural laboratory for interpreting when and how organisms disperse through time and space. However, competing hypotheses compounded with this complexity have resulted in a lack of unifying principles of biogeography for the region. Though new data concerning the timing of geologic events and dispersal events are emerging, powerful new analytical tools now allow for explicit hypothesis testing. Arthropods, with varying dispersal ability and high levels of endemism in the Caribbean, are an important, albeit understudied, biogeographic model system. Herein, we include a comprehensive analysis of every publicly available genetic dataset (at the time of writing) of terrestrial Caribbean arthropod groups using a statistically robust pipeline to explicitly test the current extent of biogeographic hypotheses for the region.

**Results:**

Our findings indicate several important biogeographic generalizations for the region: the South American continent is the predominant origin of Caribbean arthropod fauna; GAARlandia played a role for some taxa in aiding dispersal from South America to the Greater Antilles; founder event dispersal explains the majority of dispersal events by terrestrial arthropods, and distance between landmasses is important for dispersal; most dispersal events occurred via island hopping; there is evidence of ‘reverse’ dispersal from islands to the mainland; dispersal across the present-day Isthmus of Panama generally occurred prior to 3 mya; the Greater Antilles harbor more lineage diversity than the Lesser Antilles, and the larger Greater Antilles typically have greater lineage diversity than the smaller islands; basal Caribbean taxa are primarily distributed in the Greater Antilles, the basal-most being from Cuba, and derived taxa are mostly distributed in the Lesser Antilles; Jamaican taxa are usually endemic and monophyletic.

**Conclusions:**

Given the diversity and deep history of terrestrial arthropods, incongruence of biogeographic patterns is expected, but focusing on both similarities and differences among divergent taxa with disparate life histories emphasizes the importance of particular qualities responsible for resulting diversification patterns. Furthermore, this study provides an analytical toolkit that can be used to guide researchers interested in answering questions pertaining to Caribbean biogeography using explicit hypothesis testing.

## Background

**Fig. 1:**
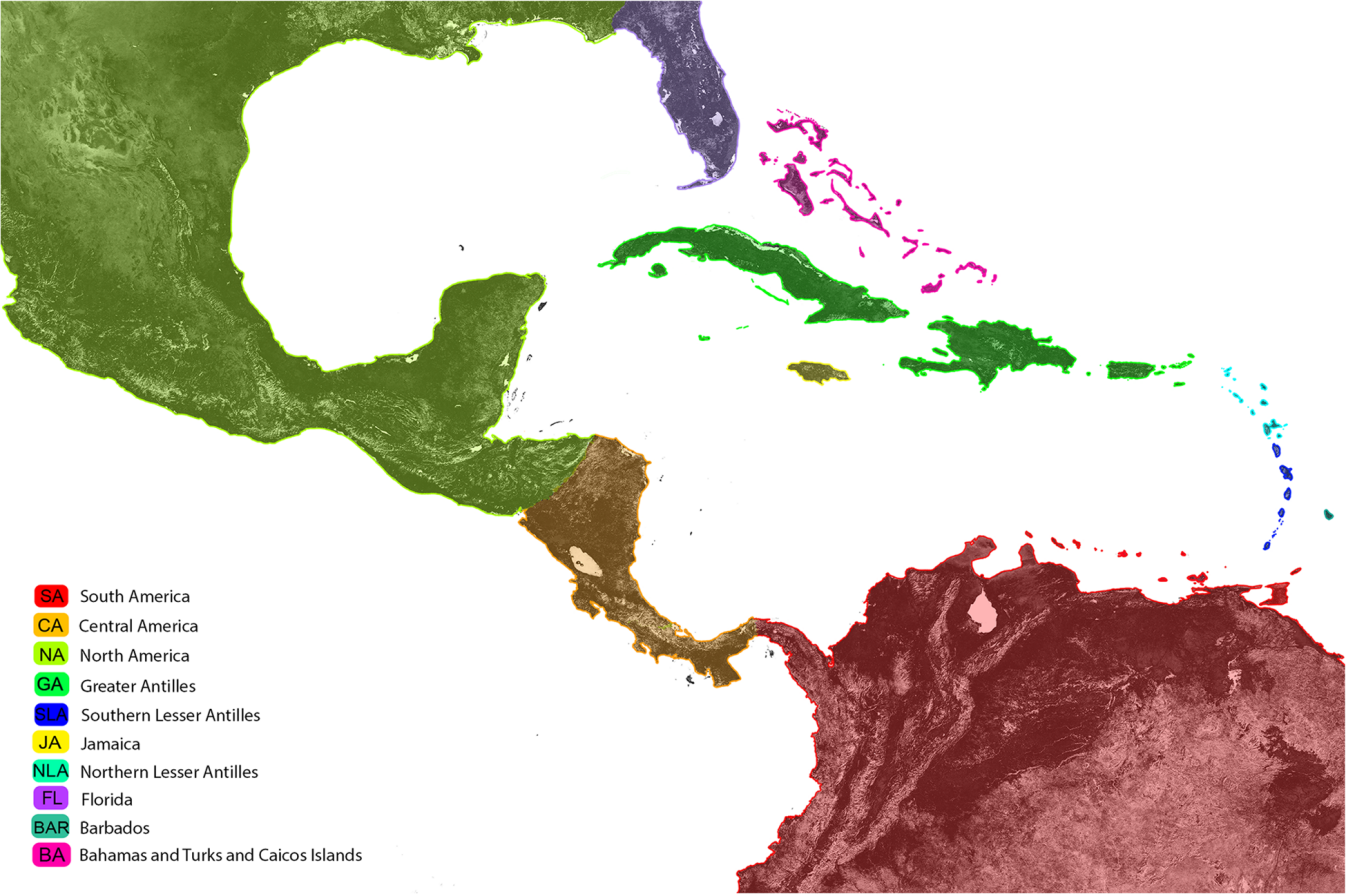
Map of the study region. The Caribbean Basin. Colors correspond to those used in subsequent figures of ancestral range estimations from BioGeoBEARS analyses. For information about geographic areas and geologic dates, see Additional file 1: Table S1. Base map created in ArcGIS v9.2 and areas shaded in Adobe Illustrator Creative Cloud. Red = South America (SA); Orange = Central America (CA); Yellow-Green = North America (NA); Purple = Florida (FL); Bright Pink = Bahamas and Turks and Caicos Islands (BA, TCI); Green = Greater Antilles (GA); Yellow = Jamaica (JA); Light Blue = Northern Lesser Antilles (NLA); Dark Blue = Southern Lesser Antilles (SLA); Blue-Green = Barbados (BAR)

The Caribbean region (Fig. 1) holds a long and rich history of entomological research and discovery. After nearly two centuries of entomological studies [1], we are still collecting and describing many new species of terrestrial arthropods (e.g., [2], and references therein, [3–7]). The > 700 islets and islands of the Caribbean (~ 240,000 km^2^), their dramatic elevational gradients (− 39 m to + 3098 m), and their proximity to two continents (North and South America) have resulted in a hyperdiversity of arthropods that can be both a boon and a pitfall for research. Unlike island systems such as Hawaii and French Polynesia where the terrestrial arthropod fauna is well-known [8], the Caribbean fauna is more diverse and less well-known, hampering areas of research such as biogeography.

The Caribbean Basin is a geologically complex region, bounded on the north, west, and south by continental landmasses. The Antillean Archipelago within the basin comprises islands with broad ages, size ranges, and elevational gradients. To the east is a volcanic arc comprising older volcanics as well as active volcanoes, and to the north are the Greater Antilles and the Bahamas Bank. While land may have been continuously available in the Greater Antilles for nearly 40 million years (my), islands such as those on the Bahama Bank are just over 100 thousand years old (kyo). Additionally, some islands, such as the Greater Antilles, may have been connected to one another and to the continental landmasses by land bridges (Wallacean islands), whereas the Lesser Antilles have never been connected to one another or the continents (de novo islands). For reviews of Caribbean geology, see [9–13] and references therein.

The primary biogeographic questions concerning Caribbean biota are how it arrived to the islands and where it is derived from. The ages of the present-day island biota have also been debated. For example, Hedges et al. [14] suggested that the bolide impact at the K-T boundary would have wiped out all organisms present on the islands, whereas Crother and Guyer [15] presented evidence to the contrary. However, geologic data [11, 12, 16] indicate that continuous land in the Greater Antilles was unavailable until after ~ 40 mya. More recent evidence about the region’s geology has shifted the discussion from whether organisms became established on islands via vicariance or dispersal to when organisms arrived and what happened after [17].

### Competing hypotheses

**Fig. 2:**
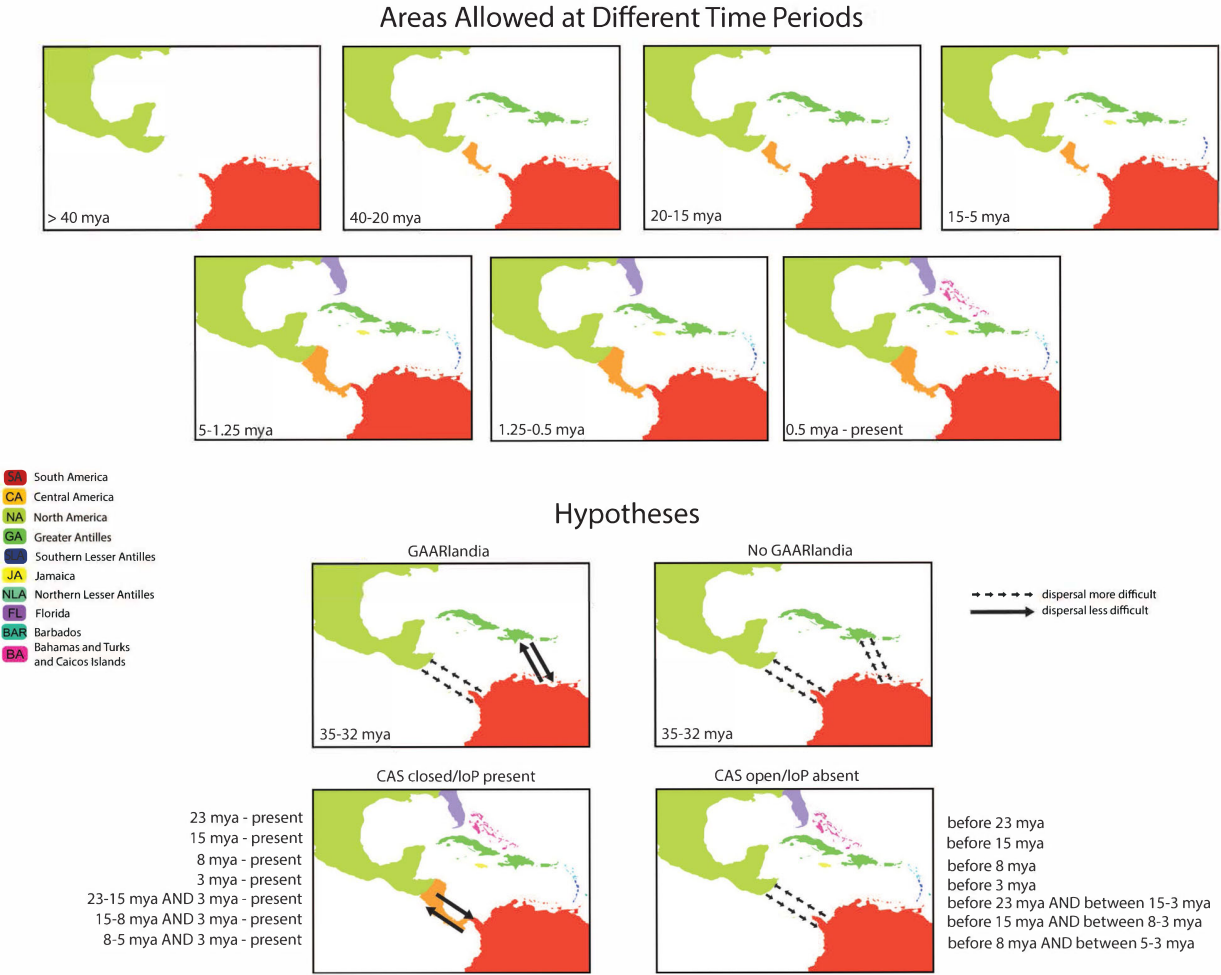
Historical Caribbean landmasses and major competing hypotheses for dispersal. Schematic representations of the Caribbean land areas hypothesized to be available at certain time periods (see Additional file 1: Table S1 for geologic references) and the major competing hypotheses examined herein (see Table 1). Arrows indicate dispersal, dates next to the images depicting the Isthmus of Panama relate to the hypothesized Central American Seaway closure(s)

In the last 25 years, new geologic and biologic evidence has allowed researchers to propound two somewhat controversial hypotheses (Fig. 2). The first being the GAARlandia (Greater Antilles-Aves Ridge) hypothesis of Iturralde-Vinent and MacPhee [11]. According to geologic and paleobiogeographic data, a landspan may have connected northern South America with the Greater Antilles 35–32 mya. This hypothesis has been invoked to explain the distribution of mammals [11], including bats [22, 23], as well as toads [24], spiders [25, 26], butterflies [27], fish [28], and plants [29, 30], although only two of these studies [25, 27] explicitly tested the GAARlandia hypothesis using statistical methods. Contrarily, some researchers (e.g., [31]) remain unconvinced that the Aves Ridge was subaerial based on the available geologic data and suggest the need for more sea-floor drilling to elucidate the extent of the land bridge or island chain.

A second equally controversial hypothesis that could greatly impact the origins of Caribbean fauna is the timing of the Central American Seaway (CAS) closure. The prevailing paradigm from both biologic and geologic data has been that the Isthmus of Panama (IoP) was completely formed (the CAS was completely closed) by the Late Pliocene, approximately 3 mya, but that the entire process occurred gradually over tens of millions of years. O’Dea et al. [18, 32] and Leigh et al. [33] and references therein review the geologic and biologic evidence for CAS closure at various times. In general, collision between the southern tip of Central America and the South American landmass began 24–23 mya ([19] and references therein), and it is thought that most of the volcanic arc was submerged; however, there is evidence that indicates significant land may have been above sea level during the Mid Miocene (~ 15–13 mya) in the form of an archipelago or a peninsula. Coates and Stallard [20] suggest a closure at 15 mya, and Osborne et al. [21] suggest the CAS closed between 12 and 7 mya. Leigh et al. [33] suggest that by 10 mya the land bridge was nearly complete, allowing some biotic interchange, and by 7 mya, most deep water populations were separated. After O’Dea et al. [18] exhaustively reviewed all available data, they concluded final closure nearer to the Late Pliocene. Bacon et al. [34, 35] used biologic data to infer geologic hypotheses, concluding an older age for IoP formation/CAS closure.

### Terrestrial arthropods in biogeographic modeling

The first study of Caribbean biogeography focused on vertebrates [36], though soon after, studies including terrestrial arthropods followed suit [37]. Arthropods are excellent study organisms for biogeography because they have relatively large populations and resilient life stages [38, 39]. Their resilience additionally means that there are often older lineages which can help clarify the effects of older geologic events [40]. Also, because similar patterns are often found between vertebrate and invertebrate taxa (e.g., [25, 41, 42]), such congruence only serves to reinforce the significance of the results (e.g., [43, 44]).

Great advances in our understanding of Caribbean arthropods have been made in the last 30 years. Liebherr [2] edited the first synthesis of Caribbean insect biogeography. Soon after, the first Caribbean biogeography studies using molecular data from insects began to emerge [45–48]. Over the past 10 years, numerous papers featuring terrestrial arthropods and focusing on historical biogeography of the Caribbean have been published (e.g., [25–27, 49–52]) as well as several phylogeographic studies (e.g., [41, 53, 54]). However, as noted by Rosen [55] and emphasized by Slater [56], “a stupendous multidisciplinary effort to resolve decisive patterns for the [Caribbean] region” is needed.

In this study we have examined all publicly available datasets of terrestrial arthropod taxa that met our criteria for selection: a taxon with a distribution across a significant part of the Caribbean Basin (> 75% land areas) with some endemic species or populations; mostly complete sampling of known lineages and distributions (> 75% across the distribution); genetic differentiation sufficient for phylogenetic analysis. Our analyses consist of ten taxa: *Platythyrea* ants, *Heterotermes* and *Nasutitermes* termites, butterflies from the genera *Calisto* and *Papilio*, *Drosophila* flies, centruroidine scorpions, and *Micrathena*, *Spintharus*, and *Selenops* spiders. These taxa provide a good representation of sedentary and more vagile taxa with various life histories. Our strategy was to maximize the sampling effort, including geographic, taxonomic, and phylogenetic coverage in order to test all of the prevailing hypotheses regarding the timing and route of dispersal into the Caribbean Archipelago. We time-calibrated phylogenies to explicitly test up to 252 biogeographic models per taxon in order to elucidate the overarching patterns of Caribbean biogeography for terrestrial arthropods.

### All models are wrong, but some are useful

We would like to make a call to biologists to interact more with geologists when studying biogeography so that the maximum information may be gleaned from the geological evidence and unlikely or impossible conclusions aren’t invoked to explain the results. Using biology to infer specific geologic events should only be done with extreme caution (e.g. – [43, 44, 57]) as the work of researchers outside of a specific discipline can be easily misinterpreted and subsequently mis-cited repeatedly in the literature. Most Caribbean biogeographic research has tried to invoke support for or against the GAARlandia land bridge (35–32 mya) using diversification rates coupled with the timing of diversification events. While the authors acknowledge GAARlandia is a controversial hypothesis, in this study the models assume that GAARlandia existed as a geologic entity, and we have evaluated the extent to which it would have played a role in dispersal into the Caribbean from South America.

There is also ongoing debate [58] about whether there are fundamental problems with biogeographic models including the ‘jump dispersal’ parameter. However, for island taxa, jump dispersal is known to be very important [59]. Portions of the Caribbean archipelago were never connected or proximal to other islands (Darwinian islands); therefore, the only route to these islands is via jump dispersal. Following this logic, jump dispersal is expected to be common in island taxa, especially those that fly or balloon, and any potential bias introduced is insignificant compared to the bias introduced by removing the ‘jump’ parameter. To fully examine this effect, we have tested 54–252 models per dataset. Similarly, we have incorporated our current understanding of fossils, molecular clocks, and geology to obtain dated phylogenies for hypothesis testing considering the current ongoing debate [60].

### A note about the term ‘colonization’

We would like to add a note about our omission of the term ‘colonization’ in this paper and make a plea to the biogeography community that this terminology be used with caution. The term ‘colonization’ has been used outside of the biology literature to refer to the arrival of European culture outside of Europe. The process of ‘colonization’ of the Americas (and many other parts of the world) by Europeans resulted in the death and enslavement of millions of people. This is particularly true in the Caribbean region. In an acknowledgement of the negative connotations associated with this term, and because none of the taxa we examined are invasive, we have chosen to omit it and instead discuss the process by which populations arrive to, and become established in, a new area by simply using the term ‘dispersal’.

## Results

For each arthropod taxon (*Platythyrea* ants, *Heterotermes* termites, *Nasutitermes* termites, *Calisto* butterflies, *Papilio* butterflies, *Drosophila* fruit flies, centruroidine scorpions, *Micrathena* spiders, *Spintharus* spiders, and *Selenops* spiders), we followed a pipeline of analyses, and the detailed results of all analyses can be found in Additional file 1. Briefly, for each, a phylogeny was constructed in MrBayes, and if the resulting topology was congruent with a published phylogeny for that taxon, then it was used as the constraint for dating in BEAST 2 and subsequent analyses in BioGeoBEARS (*Papilio*, centruroidine scorpions, *Heterotermes*). If the resulting topology was incongruent with the published phylogeny, both a topology constrained to the previously published topology and a topology constrained to our Bayesian consensus tree were used for all downstream dating and analyses (*Platythyrea*, *Nasutitermes*). In some cases, there was incongruence among published phylogenies (*Drosophila*), or irreconcilable differences between the published phylogeny and our own topology (*Micrathena*, *Spintharus*). For the former, we analyzed multiple phylogenies with various constraints based on previous studies, and for the latter, we used our Bayesian consensus tree for downstream analysis. Finally, some taxa had no published topology for which we could compare our results due to the addition of data, so the Bayesian consensus tree was used for all downstream dating and analysis (*Selenops*, *Calisto*).

Dating analyses for each taxon established minimum ages for the group. For those taxa that were younger than the proposed date of GAARlandia, the set of models which allow for the putative landspan were excluded from testing (*Platythyrea, Nasutitermes, Papilio, Drosophila, Selenops* – younger dated tree, see Table 2 and Additional file 1). Those taxa that were old enough to show any potential effect of GAARlandia were tested for the full suite of 252 models (*Heterotermes,* Centruroidinae, *Micrathena, Spintharus, Selenops* – older dated tree, see Table 2 and Additional file 1). Finally, *Calisto* is endemic to the Greater Antilles, so could only be analyzed under 18 models.

A total of 2282 biogeographic models were tested across all 10 taxa in BioGeoBEARS, and the results of biogeographic model testing for all taxa are summarized in Table 2. Resulting phylograms are shown in Figs. 3, 4, 5, 6, 7, 8, 9, 10, 11, 12, 13 and 14, and phylograms representing probabilities as pie charts can be found in Additional file 1: Figures S1–S5 and S7–S13 along with tables listing included taxa and respective GenBank reference numbers for samples (Additional file 1: Tables S5, S7–S15). All input and output files for all analyses can be found on figshare. Results from individual taxa are detailed in Additional file 1. In a few instances, the BioGeoBEARS analyses for certain models simply would not run, even when we changed starting parameters, probably because the likelihoods were too low for that particular model (Additional file 1: Table S3 and Table S4).

**Fig. 3:**
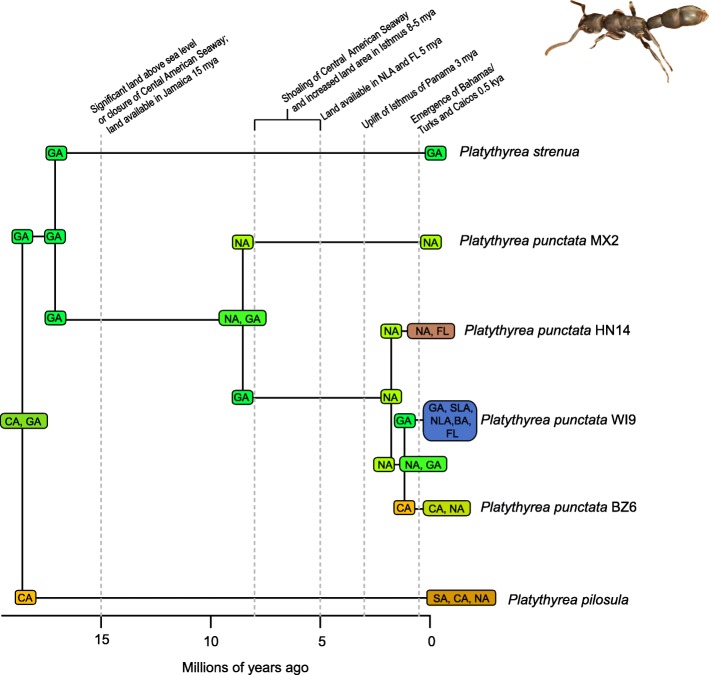
Phylogeny and ancestral range estimation for *Platythyrea* ants. BioGeoBEARS phylogram corresponding to the DIVALIKE C1a model (Table 1). The tree shows the single most probable geographic range at each node pre- and post-split. Colors correspond to Fig. 1. SA = South America; CA = Central America; NA = North America; GA = Greater Antilles; SLA = Southern Lesser Antilles; NLA = Northern Lesser Antilles; BA = Bahamas; FL = Florida. Ant photo by Judy Gallagher

**Fig. 4:**
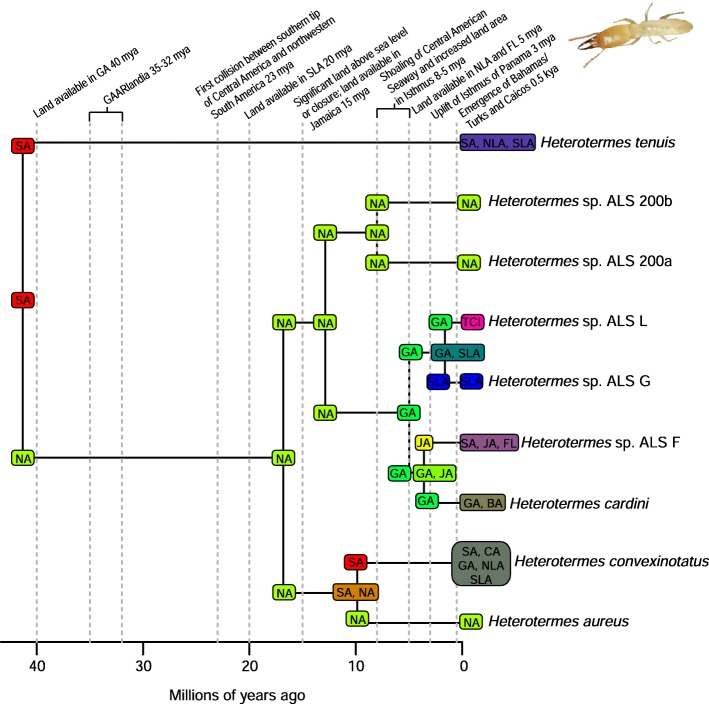
Phylogeny and ancestral range estimation for *Heterotermes* termites. BioGeoBEARS phylogram corresponding to the DIVALIKE A1a model (Table 1). The tree shows the single most probable geographical range at each node pre- and post-split. Colors correspond to Fig. 1. SA = South America; CA = Central America; NA = North America; GA = Greater Antilles; JA = Jamaica; SLA = Southern Lesser Antilles; NLA = Northern Lesser Antilles; TCI = Turks and Caicos Islands; FL = Florida. *Heterotermes* photo provided by Rudolf Scheffrahn

**Fig. 5:**
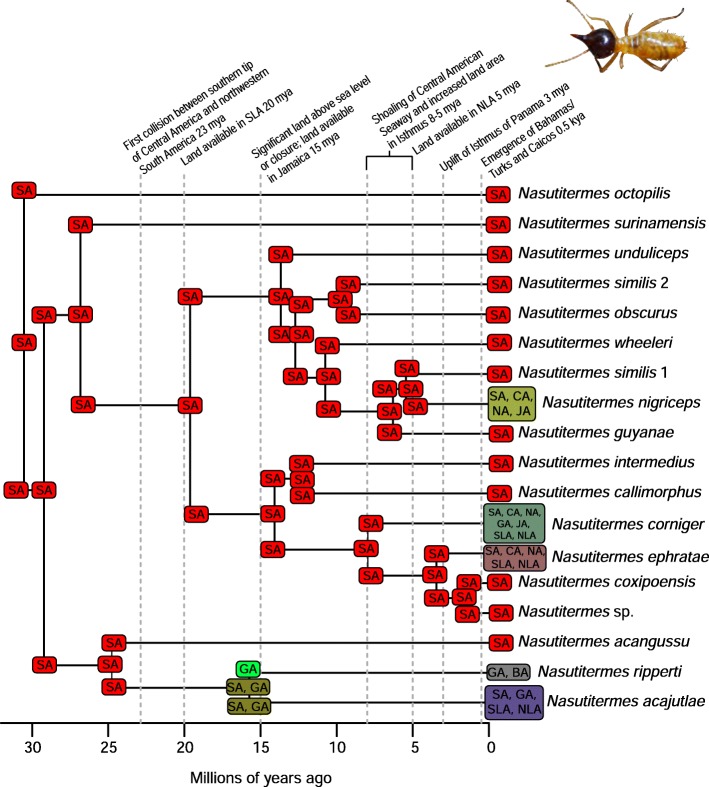
Phylogeny and ancestral range estimation for *Nasutitermes* termites. BioGeoBEARS phylogram corresponding to the DEC D1b model (Table 1). The tree shows the single most probable geographical range at each node pre- and post-split. Colors correspond to Fig. 1. SA = South America; CA = Central America; NA = North America; GA = Greater Antilles; JA = Jamaica; SLA = Southern Lesser Antilles; NLA = Northern Lesser Antilles; BA = Bahamas. *Nasutitermes* photo by Bernard Dupont

**Fig. 6:**
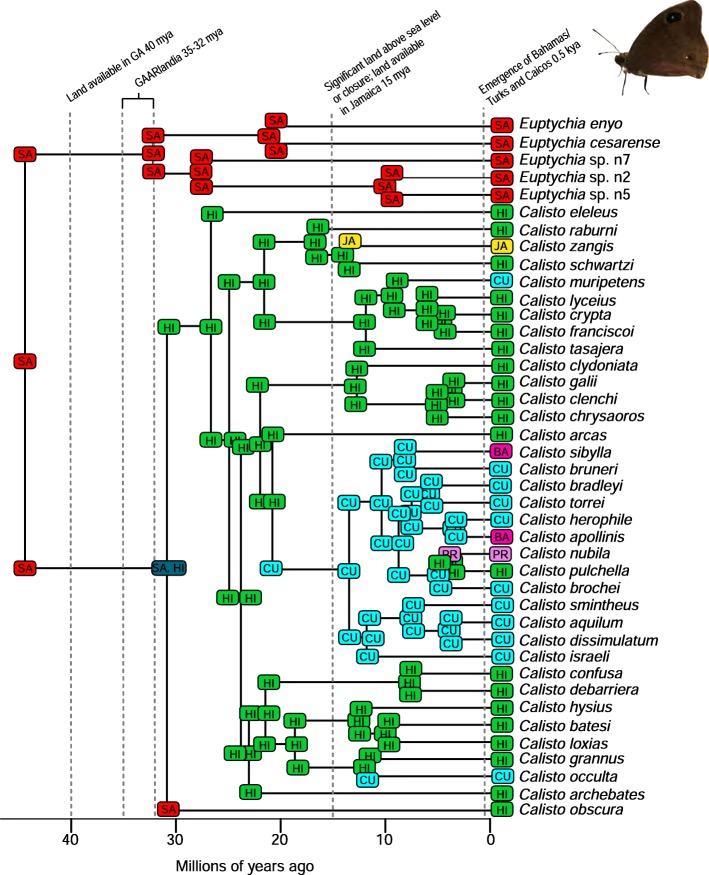
Phylogeny and ancestral range estimation for *Calisto* butterflies (*Euptychia* as the outgroup). BioGeoBEARS phylogram corresponding to the DEC + J A1b model (Table 1). The tree shows the single most probable geographical range at each node pre- and post-split. Colors correspond to Additional file 1: Figure S6. SA = South America; CU = Cuba; JA = Jamaica; HI = Hispaniola; PR = Puerto Rico; BA = Bahamas. Photo by S. Crews

**Fig. 7:**
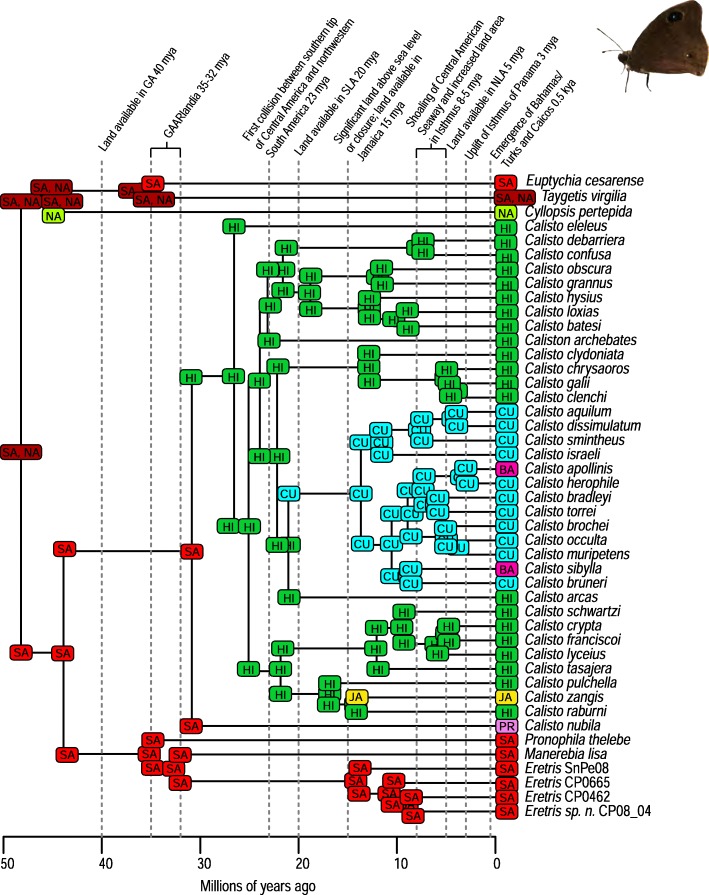
Phylogeny and ancestral range estimation for *Calisto* butterflies (*Euptychia* is not the sister taxon). BioGeoBEARS phylogram corresponding to the DEC + J A2a model (B2a, D2a, E2a, and G2a being equiprobable as the best models based on the AICc weights) (Table 1). The tree shows the single most probable geographical range at each node pre- and post-split. Colors correspond to Additional file 1: Figure S6. SA = South America; NA = North America; CU = Cuba; JA = Jamaica; HI = Hispaniola; PR = Puerto Rico; BA = Bahamas. Photo by S. Crews

**Fig. 8:**
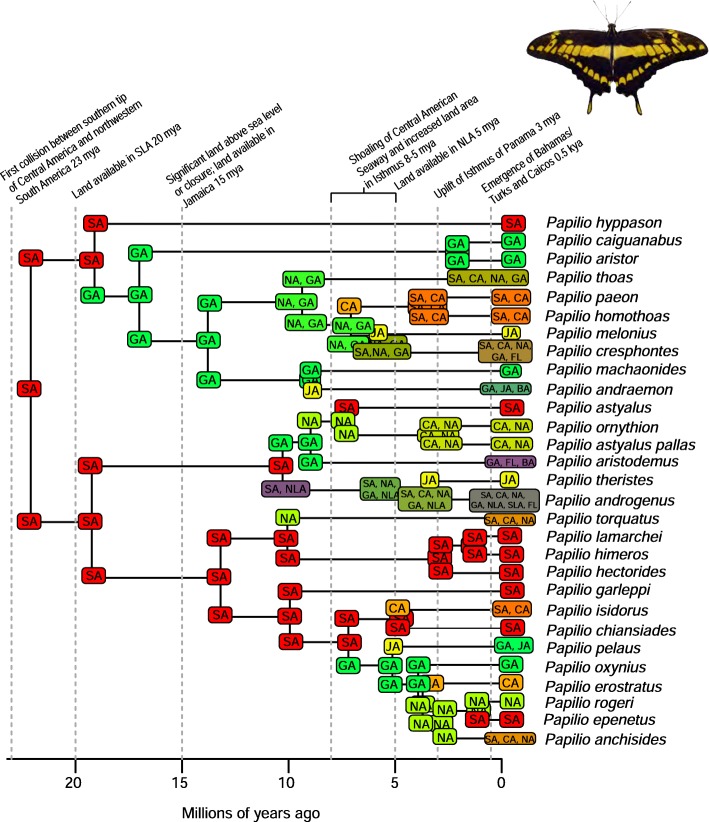
Phylogeny and ancestral range estimation for *Papilio* butterflies. BioGeoBEARS phylogram corresponding to the BAYAREALIKE + J C1a model (D1a is equally likely) (Table 1). The tree shows the single most probable geographical range at each node pre- and post-split. Colors correspond to Fig. 1. SA = South America; CA = Central America; NA = North America; GA = Greater Antilles; JA = Jamaica; SLA = Southern Lesser Antilles; NLA = Northern Lesser Antilles; FL = Florida; BA = Bahamas. *Papilio* photo by Eduardo Lopez

**Fig. 9:**
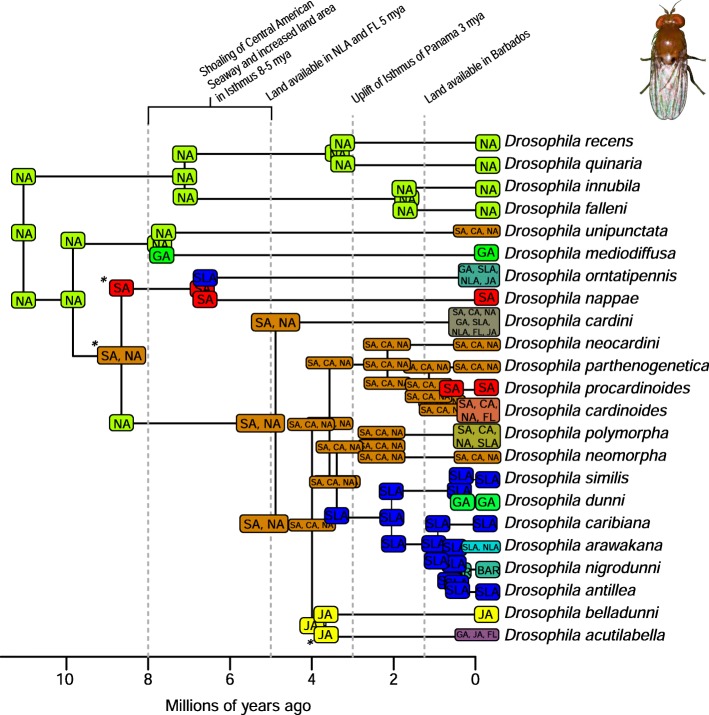
Phylogeny and ancestral range estimation for *Drosophila* flies. BioGeoBEARS phylogram corresponding to the BAYAREALIKE + J D1a model (Table 1) for the tree with the relationship of *D. belladunni* and *D. acutilabella* constrained (see Additional file 1). Asterisks indicate differences between the constrained and unconstrained trees in the ancestral range estimation. The tree shows the single most probable geographical range at each node pre- and post-split. Colors correspond to Fig. 1. SA = South America; CA = Central America; NA = North America; GA = Greater Antilles; JA = Jamaica; SLA = Southern Lesser Antilles; NLA = Northern Lesser Antilles; FL = Florida; BAR = Barbados. *Drosophila* photo by Mark Yokoyama

**Fig. 10:**
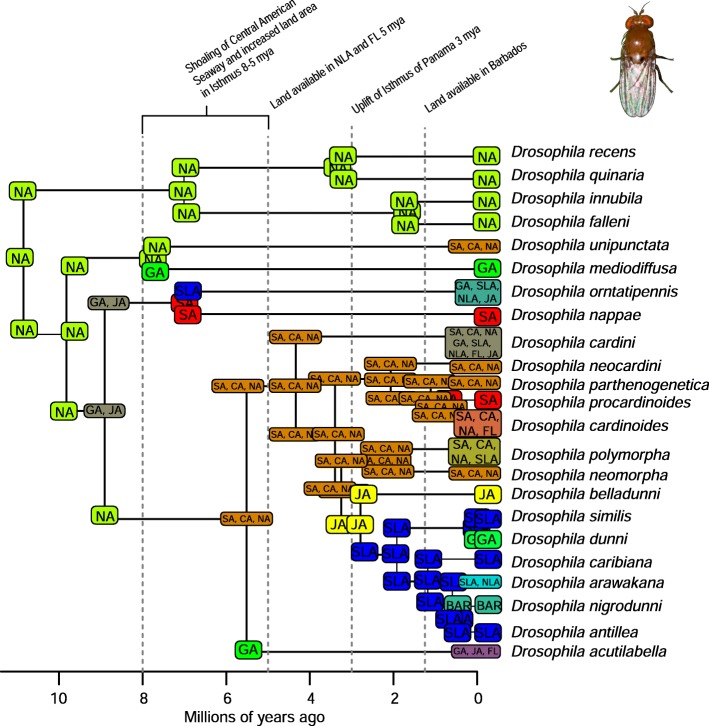
Phylogeny and ancestral range estimation from BioGeoBEARS for *Drosophila* flies without constraining the relationship of *D. belladunni* and *D. acutilabella.* The best model based on AICc weights is BAYAREALIKE + J D1a (Table 1). The tree shows the single most probable geographical range at each node pre- and post-split. Colors correspond to Fig. 1. SA = South America; CA = Central America; NA = North America; GA = Greater Antilles; JA = Jamaica; SLA = Southern Lesser Antilles; NLA = Northern Lesser Antilles; FL = Florida; BAR = Barbados. *Drosophila* photo by Mark Yokoyama

**Fig. 11:**
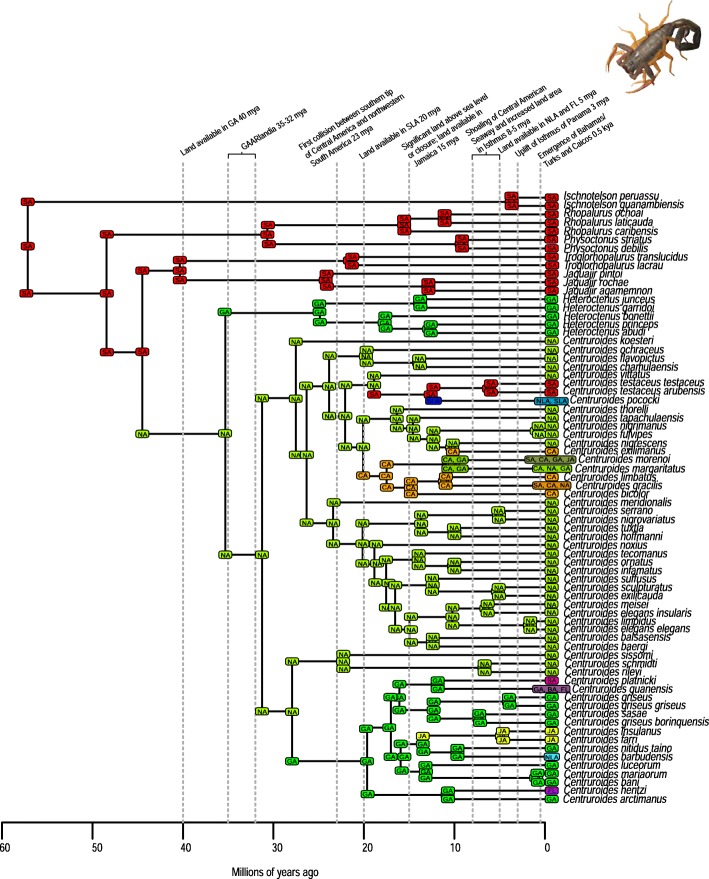
Phylogeny and ancestral range estimation for centruroidine scorpions. BioGeoBEARS phylogram corresponding to the BAYAREALIKE + J B1a and B2a models (Table 1). The tree shows the single most probable geographical range at each node pre- and post-split. Colors correspond to Fig. 1. SA = South America; CA = Central America; NA = North America; GA = Greater Antilles; JA = Jamaica; SLA = Southern Lesser Antilles; NLA = Northern Lesser Antilles; FL = Florida; BA = Bahamas. Photo by S. Crews

**Fig. 12:**
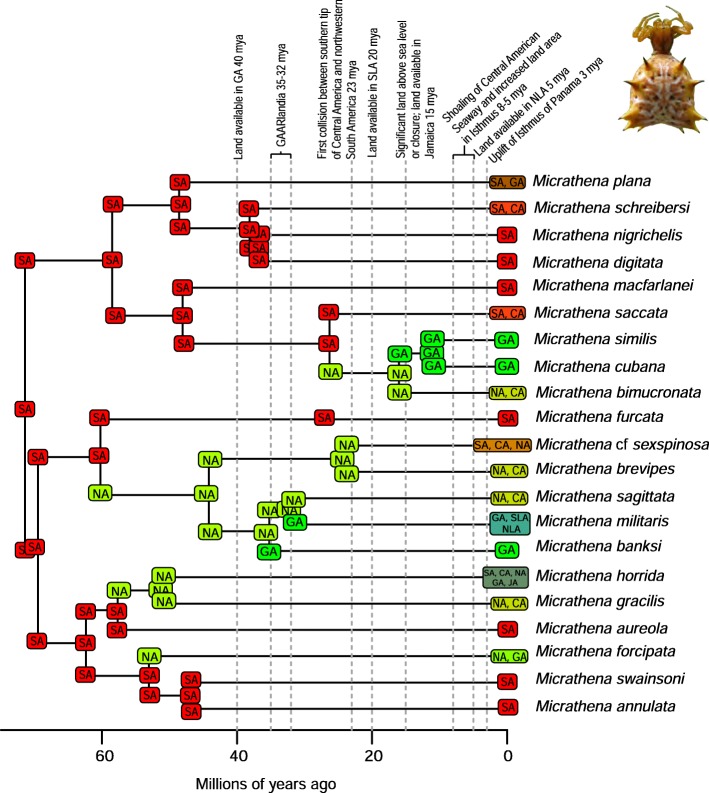
Phylogeny and ancestral range estimation for *Micrathena* spiders. BioGeoBEARS phylogram corresponding to the DIVALIKE + J B2a model (Table 1). The tree shows the single most probable geographical range at each node pre- and post-split. Colors correspond to Fig. 1. SA = South America; CA = Central America; NA = North America; GA = Greater Antilles; JA = Jamaica; SLA = Southern Lesser Antilles; NLA = Northern Lesser Antilles. Photo by S. Crews

## Discussion

Our analyses indicate some shared patterns and processes of dispersal to and subsequent diversification on islands in the Caribbean biodiversity hotspot, including dispersal across the IoP/CAS (Table 2). Below we discuss some of the major themes surrounding the evolution and biogeography in the Caribbean. These data provide a baseline for indicating the types of questions that can be confidently answered given the available data and highlight where knowledge is lacking and can be improved upon in future research. We base our discussion on the phylogeny that shows the single most probable ancestral range for each taxon; for trees that show the probabilities for each possible range, see Additional file 1.

### Origins of Caribbean taxa

South America is the most common place of origin for the Caribbean fauna examined, followed by Central America and North America (Table 2). This is congruent with the generalized track analysis and cladistic biogeography of Morrone [61] and Echeverry and Morrone [62] for many arthropod (and other) taxa. There does not seem to be a shared evolutionary history, ecology, or life history among groups that share a point of origin, and this does not appear to coincide with the timing of dispersal or age of the group in question. Some taxa appear to have dispersed to the islands from more than one area of the mainland, including a relatively young group of good dispersers (*Drosophila*) [63], and a relatively old group of poor dispersers (*Micrathena*) [26] (Figs. 9, 10, 12). These results are congruent with other literature on Caribbean arthropods; for example, beetles are also thought to have dispersed to the islands from both Central America and South America [40, 64], and ants [65] and auchenorrhynchous homoptera [66] are thought to have dispersed to the Caribbean from both Central America and North America.

### Number of dispersal events to Caribbean islands

We find that all but the poorest dispersers have arrived in the Caribbean multiple times (Table 2). The primary predictor of the number of dispersal events into the Caribbean appears to be dispersal ability, including mode of locomotion, as well as adaptability and ecological plasticity, which are important for establishment after dispersal. However, extinction events could also produce patterns of multiple dispersal events. It should be noted that the plot of most-probable areas is not the same as event counts [67]. Simple counts give some idea of event number but do not consider events occurring along a branch; for better estimates, biogeographic stochastic mapping is required.

Among flying insects, there is a general pattern of multiple dispersal events. *Drosophila* fruit flies, *Papilio* butterflies, and *Nasutitermes* termites each dispersed at least 4 times (Figs. 5, 8, 9 and 10), and *Heterotermes* termites and *Calisto* butterflies dispersed twice (Figs. 4, 6, 7). *Drosophila* is thought to be a good disperser [63], and the groups examined here are thought to have dispersed relatively recently (~ 4 my) to the Caribbean, perhaps indicating that dispersal ability has played a role in the number of dispersal events to the islands. *Papilio*’s dispersal abilities have been noted by Lewis et al. [52] who also found several separate dispersal events rather than a single dispersal followed by diversification (Fig. 8). In contrast, *Calisto* butterfly species have only dispersed one or two times (depending on the outgroup used) to the Greater Antilles from South America (Figs. 6, 7). Among the termites, *Heterotermes* dispersed at least 3 times from South America and North America (Fig. 4). However, according to our results, *Nasutitermes* is relatively new in the Caribbean (~ 15 my) but appears to be a good disperser (Fig. 5). In at least one species (*N. corniger*), Scheffrahn et al. [68] noted that kings and queens are quite good flyers and can adapt to many different types of habitats, from urban to more natural, and are good at taking over and defending territories.

**Fig. 13:**
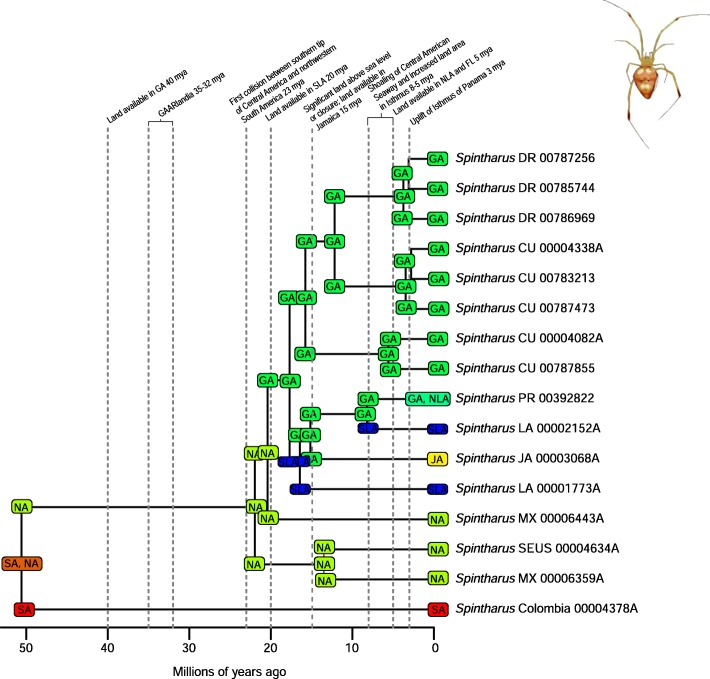
Phylogeny and ancestral range estimation for *Spintharus* spiders. BioGeoBEARS phylogram corresponding to the DIVALIKE + J A1b model (Table 1). The tree shows the single most probable geographical range at each node pre- and post-split. Colors correspond to Fig. 1. SA = South America; CA = Central America; NA = North America; GA = Greater Antilles; JA = Jamaica; SLA = Southern Lesser Antilles; NLA = Northern Lesser Antilles. Photo by Judy Gallagher

**Fig. 14:**
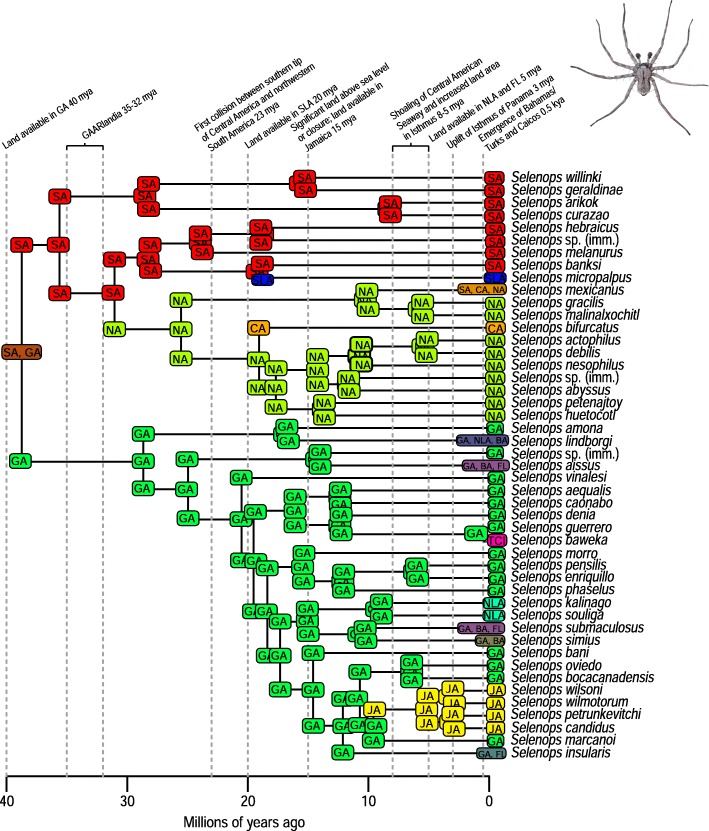
Phylogeny and ancestral range estimation for *Selenops* spiders. BioGeoBEARS phylogram corresponding to the DIVALIKE + J B1a model (Table 1). The tree shows the single most probable geographical range at each node pre- and post-split. Colors correspond to Fig. 1. SA = South America; NA = North America; CA = Central America; GA = Greater Antilles; JA = Jamaica; SLA = Southern Lesser Antilles; NLA = Northern Lesser Antilles; FL = Florida; TCI = Turks and Caicos Islands; BA = Bahamas. Photo by S. Crews

*Selenops* spiders have dispersed to Caribbean islands only twice, from South America to the Lesser Antilles for one event, and to the Greater Antilles for the second event, although the point of origin remains unclear (Fig. 14). Within *Centruroides* scorpions, there are also two dispersal events, one from South America and one from North America (Fig. 11). Both *Selenops* spiders and *Centruroides* scorpions likely have a similar ecology as nocturnal somewhat stationary sit-and-wait predatory arachnids. *Spintharus* spiders have seemingly only dispersed to the Caribbean once, and although this species is thought to be a poor disperser [51], it does appear capable of dispersal to proximal islands (Fig. 13).

Somewhat puzzling are *Micrathena* spiders, considered to be a poor dispersersing taxon [26], they have dispersed to the Caribbean multiple times (6 in our analyses) (Fig. 12). One possible explanation is, as our analyses indicate, that they are a rather old group which would allow for more time in which dispersal events could occur. Missing taxa from the mainland could also produce a pattern of multiple dispersal events. Nevertheless, other studies with more taxa sampled also indicated multiple dispersal events [69], leading us to conclude that this taxon is in fact a rather good disperser like many other orb-weaving spiders.

Szalanski et al. [48] suggested that the present distribution of *Heterotermes* in the Caribbean is likely due to overwater dispersal, however he emphasized that anthropogenic introductions couldn’t be ruled out. In our analyses, it does appear that most species lineages are at least a few million years old (Fig. 4), but that some of the intraspecific distributions may be anthropogenically caused. Human-mediated dispersal, even intraspecific dispersal within the native range, could have a significant impact on biogeographic results. In some cases, this may be a very evident pattern which emerges from the data, however, there may be examples such as this where it is cryptic and confounds the conclusions that can be drawn about the taxon.

### Islands as sources of diversity

Islands are not usually thought of as sources for diversity, but rather sinks [70, 71]. Recently, however, more examples of dispersal from an island source in the Caribbean has become more well-known, particularly in *Anolis* lizards, including from a large island to smaller islands or even from islands to the mainland [72, 73]. Dispersal from the islands to the mainland is also seen in weevils [74] and orioles [75]. One reason we may not be as aware of this phenomenon is because mainland sampling from Mexico, Central America, and South America is often poor and therefore unable to provide the phylogenetic resolution required to detect such events; even excluding a single species could change the results.

There are many examples from our analyses of dispersal from Cuba to the Bahamas, perhaps due to these land areas being particularly close to one another during times of low sea level. Although we only used a single specimen of *Nasutitermes ripperti* from the Bahamas in our analyses, this species is also found in Cuba, so perhaps dispersed from Cuba to the Bahamas. In *Calisto* butterflies, dispersal from Cuba to the Bahamas has occurred at least twice (Fig. 13). In *Selenops* spiders and *Centruroides* scorpions, taxa are shared between Cuba and the Bahamas and presumably dispersed from Cuba to the Bahamas (Figs. 11, 13, 14). Similarly, the large island of Hispaniola is a source of fauna for the small Turks and Caicos Islands in one *Heterotermes* termite lineage, *Selenops* spiders, and *Centruroides* scorpions (Figs. 4, 11, 13, 14).

Contrarily, there are instances of smaller islands as sources of taxa for larger islands, or even the mainland. *Drosophila* is unique in that there seems to have been a small species radiation in the Southern Lesser Antilles (Figs. 9, 10), and from this, two dispersal events occurred to the Greater Antilles (*D. ornatipennis* and *D. dunni*). Additionally, it appears that one lineage (*D. acutilabella*) emerged from a dispersal event to the Greater Antilles from Jamaica. Our analysis of *Heterotermes* indicates that one species (*H. tenuis*) from Guadeloupe island is basal to the South American lineage, with the caveats that sampling across the range of the species is poor, and these data are from a single gene (Fig. 4). In *Platythyrea* ants, there have been at least 2 dispersal events from the Greater Antilles to North America (Fig. 3).

Notably, the two butterfly species we examined show contrasting patterns of dispersal. *Papilio* are good dispersers and don’t seem to be affected by allopatric speciation or within-island vicariance, so whereas there are many species on the islands, ranges are broad and large species radiations do not appear to have occurred (Fig. 8). *Calisto* butterflies, however, are small and poor fliers. These butterflies show no evidence of dispersal from islands to the mainland and very few examples of dispersal from one island to the next; however, they are very diverse within islands likely due to being habitat specialists as well as poor dispersers (Figs. 6, 7) [27]. For Neotropical butterflies, Condamine et al. [76] emphasized the importance of islands as sources of diversity for the mainland. In *Papilio* butterflies there have been multiple dispersal events from the Greater Antilles to North America or to South America. This result differs from those of Lewis et al. [52] who found several “reverse” dispersal events, or events where the taxon is returning to the ancestral range of origin (dispersal from islands to the mainland); they found the entire clade of *Heraclides* to have a Caribbean origin whereas our analyses reveal a South American origin. Nevertheless, both analyses found that islands can be a source of diversity for other islands or even the mainland. Lewis et al. [52] attributed this in part to *Papilio* being big, strong fliers that favor edge habitats.

### Founder event dispersal

The ability of BioGeoBEARS to incorporate “jump” dispersal into models has been advantageous in biogeographic studies (but see [58]). The program allows for models that emphasize the importance of founder event dispersal in island taxa [59]. In our analyses, models incorporating jump dispersal were favored in 8/12 analyses and were more common in taxa with older origins in the Caribbean (pre-35 my) (Table 2). However, in selenopid spiders, it was favored in the analysis with the younger tree, but not the older tree (see Additional file 1). The only analyses where a model incorporating jump dispersal was not favored were of ants and termites—organisms that only fly for a part of their adult life. Our results indicate that jump dispersal plays a role for both good dispersers, like *Papilio*, and poor dispersers, like spiders and scorpions. Founder event dispersal was always the favored model of dispersal for spiders and animals that fly during their entire adult life stage (*Calisto*, *Papilio*, and *Drosophila*). This finding corroborates results from Matos-Maraví et al. [27] for *Calisto* butterflies and Zhang et al. [74] for weevils.

### Distance between areas of dispersal

To understand the influence of distance between neighboring landmasses (islands or continents) on dispersal probability we used manual dispersal multiplier (mdm) matrices in BioGeoBEARS analyses. Despite incorporating jump dispersal into our models, a model in which dispersal was very difficult (mdm values set to 0.01) was never favored (Table 2). We did find that in most taxa, distance-dependent models were favored (*Platythyrea* ants, *Heterotermes* termites, *Papilio* butterflies, centruroidine scorpions, *Micrathena* and *Selenops* spiders). For *Calisto* butterflies, distance did not affect dispersal when *Euptychia* was used as the sister taxon but did affect dispersal when other taxa were used. In *Drosophila*, two models were favored, and in one, distance affected dispersal, but in the other it did not.

Geologically, the Lesser Antilles islands can be divided into volcanic islands and two limestone volcanic arcs with the Southern Lesser Antilles older than the Northern Lesser Antilles (Additional file 1: Table S1). There were many episodes of gradual emergence, even within single islands, so a strict northern vs. southern boundary is elusive. These islands are younger and smaller than the Greater Antilles and have never been connected to the mainland, lowering overall dispersal probability. Previous biogeographic studies have recovered a clear pattern of a genetic split between organisms, congruent with Northern/Southern Lesser Antilles geology. This faunal-island split isn’t always between sister taxa, such as in *Anolis* lizards, *Selenops* spiders, and *Centruroides* scorpions where it appears there were two dispersal events: one from the Greater Antilles south to the Northern Lesser Antilles and one from South America north to the Southern Lesser Antilles, but without any discreet faunal break between islands. In this and a previous analysis [41], selenopid spider distribution agrees with the geologic data in that dispersal from South America to the older islands occurred sometime after 15 mya, whereas taxa didn’t disperse to the Northern Lesser Antilles until after 8 mya and are instead derived from the Greater Antillean fauna. The precise location of the split is also highly variable; for example, in *Anolis* lizards [77], it occurs between Martinique and Dominica, however it occurs further south between St. Vincent and St. Lucia [46] in lygaeid bugs [56], carabid beetles [40], *Eleutherodactylus* frogs [78], and bananaquits [79]. The location of the split recovered in *Selenops* spiders occurs between Dominica and Les Saintes, Guadeloupe. In the termite *Heterotermes tenuis,* we recovered some population structure between northern (Guadeloupe) and more southern islands (St. Lucia, St. Vincent, Grenada, Trinidad) [48]. For *Dryas iulia* butterflies [46] there have been multiple splits inferred, between both Martinique/Dominica and St. Vincent/St. Lucia. Multiple splits were also recovered in this study in *Spintharus* spiders: one between St. Kitts and Nevis, and another between Dominica and more southern islands.

### Dispersal across the Isthmus of Panama/Central American Seaway

It should be noted that we are not aiming to disprove or provide evidence for a geologic hypothesis using biologic data, nor are we suggesting that our results indicate that the Central American Seaway had completely receded > 3 mya. The taxa we have examined do not require a land connection to be able to disperse and diversify across aquatic barriers. Most of our results indicate that the favored biogeographic model includes a single dispersal event between North and South America that occurred prior to 3 mya (Table 2). This result was also found for Hercules beetles [80], where it was suggested that the beetles could fly over water for short distances or that larvae could raft in downed wood. Taxa in which older dispersals (23 mya) were favored are centruroidine scorpions (Fig. 11), *Micrathena* spiders (Fig. 12), and *Selenops* spiders (Fig. 14). *Selenops* spiders and centruroidine scorpions are known from Chiapas and Dominican amber, indicating they have been present in southern North America and the Caribbean for at least 20 my.

The only taxon with a favored model of earliest dispersal at 15 my is *Platythyrea* ants (Table 2; Fig. 3). Seal et al. [53] mention that the ants probably disperse by walking over land, so perhaps they dispersed via a land connection or floated on flotsam. For some taxa, the models couldn’t be differentiated to determine a favored one. This was true in the analysis of *Calisto* butterflies, where 5 models were equiprobable (Table 2). In *Nasutitermes* and *Papilio*, both 15 and 8 my were favored (Table 2, Figs. 5, 8).

Several taxa were recovered with a favored model of 8 my or younger: *Drosophila* fruit flies, *Spintharus* spiders, and *Heterotermes* termites (Figs. 4, 9, 10, 13). Other studies have determined dispersal across the IoP/CAS to have occurred prior to 3 my, including those of butterflies [76] and *Loxosceles* spiders [81], although in the latter, the authors suggest dispersal occurred via a land bridge, which according to geologic data is not possible [18]. Bagley and Johnson [17] suggest that earlier dispersals were bidirectional and could have occurred via island hopping. Having better Central American sampling where possible, especially in taxa where we know there are species missing from the analyses, would certainly improve the level of uncertainty.

### The role of GAARlandia in shaping Caribbean arthropod biodiversity

Several of the datasets we analyzed were younger than 32 mya, precluding GAARlandia as a transport path from South America to the Greater Antilles for these taxa. GAARlandia also did not play a role in the dispersal of *Heterotermes* termites, *Selenops* spiders, or *Spintharus* spiders (Figs. 4, 13, 14). In many cases our findings contrast those of previous research, although these studies lacked thorough sampling for parts of the distribution [25, 51] or hypothesis testing was not conducted [51]. For example, Dziki et al. [51] suggested that GAARlandia played a role in the dispersal of *Spintharus* from South America to the Greater Antilles. However, this was based on the coincidental timing of the diversification of a Caribbean clade rather than hypothesis testing. Our results, based on the relative AICc weights of the BioGeoBEARS analyses, favored a dispersal model without GAARlandia and also recovered a later date of dispersal into the Caribbean (~ 20 my). The results we obtained for *Selenops* spiders contradict those of Crews and Gillespie [25] in which similar hypothesis testing methods using Lagrange [82, 83] (which only uses the DEC model) found that models including GAARlandia were supported. Ancestral range estimation (Fig. 3) suggests that *Selenops* was in the Greater Antilles shortly after 40 my, before GAARlandia is hypothesized to have existed, and the model favored includes the jump dispersal parameter (Fig. 14, Table 2). This difference could be due to better dating methods now available, the use of different geologic dates/time slices, differences in the programs Lagrange and BioGeoBEARS, including the incorporation of jump dispersal, or the addition of more taxa/data, indicating how missing taxa affect biogeographic conclusions. For *Micrathena* spiders, the favored model indicated that GAARlandia played a role in dispersal to the Greater Antilles (Fig. 12). This is slightly confounding given the ancestral range estimation. Although there was dispersal to the Greater Antilles around 35–32 mya, these taxa came from North American ancestors. These findings contrast the results of McHugh et al. [26], and more sampling is required to explain these results. In centruroidine scorpions, two models had equal relative probabilities, one that included GAARlandia and another that did not (Fig. 11, Table 2). Esposito and Prendini [84], using Lagrange, previously found that the best fitting model was a distance-dependent dispersal model that included GAARlandia. According to the ancestral range estimation (Fig. 11), the timing of *Heteroctenus* scorpions entering the Caribbean is nearly simultaneous with the proposed timing of GAARlandia; however, the ancestral range estimation indicates a North American origin (although with some uncertainty), contradicting the results of Esposito and Prendini [84], who recovered a South American + Greater Antilles ancestral range using RASP [85, 86]. In *Calisto* butterflies, the results depend on whether *Euptychia* is the sister taxon or if other outgroups are used, emphasizing the importance of outgroup selection. For the former, distance-independent dispersal without GAARlandia was chosen as the favored model (Fig. 6). For the latter, distance-dependent dispersal with GAARlandia was the favored model (Fig. 7).

### Species-area relationships and endemism

MacArthur and Wilson [70] suggested that island area may be responsible for lineage diversity or species numbers on islands. We know that the way in which island area is measured, habitat diversity, island age, and distance from the mainland are also important determiners [8, 87–90]. Generally, for all taxa examined, the Greater Antilles harbor more species than the Lesser Antilles, and the larger Greater Antilles islands, like Cuba and Hispaniola, typically have more species than the smaller islands of Puerto Rico and Jamaica. This expected pattern of Cuba > Hispaniola > Jamaica > Puerto Rico generally holds for the bug family Lyagaeidae [56], pseudoscorpions [91], scaritine beetles [92], *Centruroides* scorpions (Fig. 11), *Micrathena* spiders (Fig. 12), and *Papilio* butterflies (Fig. 8). However, in many taxa there are more species in Hispaniola than Cuba, such as in *Platynus* carabid beetles and *Amphiacusta* crickets [40, 49], the latter with 18 species in the Lesser Antilles. In *Calisto* butterflies, Hispaniola has nearly double the species of Cuba, and there are 2 species in the Bahamas but only 1 each in Puerto Rico and Jamaica. Grimaldi [63] suggested that there may be less endemism in fruit flies because they are good dispersers whose ranges usually cover large areas. This appears to be true in the cardini and dunni groups examined here, which have only a few species in the Greater Antilles and few single island endemics in the Lesser Antilles (Figs. 9, 10). For selenopid spiders, there are more endemics in Hispaniola than in Cuba (Fig. 12), although both islands harbor the same total number of species. Reasons for discrepancies from the typical pattern are worth a second look, and in the case of Cuba vs. Hispaniola could relate to differences in the toplogical complexity.

### Lineage age and lineage radiations

Older lineages found on the Greater Antilles are generally ancestral to those of the Lesser Antilles; and Cuba, Hispaniola, or Puerto Rico lineages diverge earlier than Jamaican lineages. Species occurring in Cuba are the most basal for *Selenops* and the Caribbean endemic clade of *Centruroides* scorpions (Figs. 11, 14). Hispaniolan lineages are basal in *Platythyrea* ants and *Calisto* butterflies when *Euptychia* is used as a sister group; alternatively, Puerto Rican lineages are basal, which is also found in weevils [74] (Figs. 3, 6). Jamaican lineages are basal in *Argiope* spiders [93], however, it is possible that *Argiope butchko* is not a valid species but another lineage of *Argiope argentata*, in which case Cuban lineages would be basal. If we accept the proposal of Agnarsson et al. [93], then *A. argentata* has managed to disperse throughout the Caribbean to the exception of Cuba. Although competition could perhaps prevent *A. argentata* from becoming established in Cuba, multiple species of *Argiope* can be found in sympatry all over the world (e.g., [94]). *Argiope butchko* was described based solely on a few Cox1 differences and appears to show no morphological differences from *A. argentata*).

Lineage radiations have occurred in Cuba and Hispaniola in the butterfly species *Calisto* and *Heteroctenus* scorpion species, and all are single island endemics (Figs. 6, 7, 11). In species from the Caribbean clade of *Centruroides* scorpions, most exhibit patterns of within-island diversification or are single island endemics (Fig. 11). Considering that *Calisto* and scorpions are poor dispersers, the pattern of a single dispersal event followed by within-island radiation is expected. Conversely, *Papilio*, a good disperser, has not diversified on islands. *Selenops* spiders have undergone multiple lineage radiations in Hispaniola and Jamaica, and likely in Cuba, but without improved sampling it is unclear how many.

### Island monophyly

Some groups exhibit patterns of dispersing to an island only once, whereas others have dispersed to a single island multiple times. Sampling is obviously important when examining island monophyly because missing species could cause false negatives as well as false positives. There does not appear to be island monophyly for the majority of taxa, including *Papilio* butterflies (Fig. 8), *Drosophila* flies (Figs. 9 and 10), *Micrathena* (Fig. 12) and *Selenops* spiders (Fig. 14), *Platythyrea* ants (Fig. 3), termites (Figs. 4 and 5), or weevils [74]. In the analysis of *Calisto* with *Euptychia* as the sister taxon, the butterflies have dispersed to Cuba at least 3 times from Hispaniola, and to Hispaniola at least twice, but to Jamaica and Puerto Rico only once (Fig. 6). However, in the analysis without *Euptychia* as the sister taxon, the butterflies dispersed to Cuba and Hispaniola only once each (Fig. 7). In *Spintharus* spiders there appears to be 1 Jamaican lineage, 1 Hispaniolan lineage, and 1 Puerto Rican lineage, but 2 Cuban lineages (Fig. 13). In *Argiope argentata*, lineages on Hispaniola and Jamaica are monophyletic [93]. Among scorpions, taxa are almost always monophyletic on islands, with the exception of 2 Cuban lineages of *Centruroides*.

Although Jamaica is considered one of the Greater Antilles, it has a rather different geologic history than Cuba, Hispaniola, and Puerto Rico. The island began emerging 40–30 mya and is thought to have had continuous available land since 15 mya, whereas the other Greater Antilles may have had continuous available land for much longer (~ 40 my). It is plausible that the Blue Mountains Block of eastern Jamaica has been above sea level for much longer and/or the part of western Jamaica that was attached to the Nicaraguan Rise of Central America was never submerged. Jamaica appears to have had a unique biogeographic history compared to the other Greater Antilles [95]. Of taxa included in this study, there are Jamaican endemic lineages in *Calisto* (1 species), *Papilio* (2 species), fruit flies (2 species), *Centruroides* scorpions (clade of 2 endemic species), *Micrathena* (1 species), *Argiope* (clade), *Spintharus* (clade), and Selenopid spiders (clade of 4 endemic species).

## Conclusions

Terrestrial arthropods represent an immense wealth of biodiversity, dispersal abilities, life history traits, and ecologies. Their species assemblages in the Caribbean region underpin the importance of the Caribbean as both a major gateway for faunal exchange between the continents of the Western Hemisphere as well as a hotspot of endemic biodiversity and diversification. Our results indicate several biogeographic generalizations for terrestrial arthropods: South America is the predominant origin of the Caribbean arthropod fauna, and GAARlandia may have played an important role in aiding dispersal from South America to the Greater Antilles for some taxa but not universally. Founder (“jump” dispersal) events explain the majority of dispersal into the Caribbean, and most dispersal events occurred from the mainland to the islands and subsequent island hopping; this holds true for both good and poor dispersers. Distance is an important predictor of dispersal between landmasses, and there is evidence of islands as sources of diversity for the mainland in some taxa. Dispersal across what is now the Isthmus of Panama generally occurred prior to closure of the Central American Seaway 3 mya. The Greater Antilles harbor more species than the Lesser Antilles, and the larger Greater Antilles islands, Cuba and Hispaniola, typically have more species than the smaller islands of Puerto Rico and Jamaica. Among the Caribbean endemic taxa, basal taxa are most often distributed in the Greater Antilles, with the basal-most being Cuba, and derived taxa are most often distributed in the Lesser Antilles. Jamaican taxa are usually younger, endemic, and monophyletic for the island, whereas this is not the case for the other Greater Antillean islands.

The data presented herein provide a good baseline for understanding the biogeography of terrestrial arthropods in the Caribbean through explicit hypothesis testing. These methods allow for reproducible science and an improvement of our understanding of geologically complex regions. As genomic data continue to become more commonplace, genetic data from older museum specimens can be obtained, filling in gaps and helping resolve phylogenies and improve dating inferences. We hope that this paper will serve as a guide to help researchers determine what sorts of questions they can ask and answer with the data available to them.

## Methods

### Selection of datasets

We selected datasets using the following criteria: 1) *Availability of a phylogenetic hypothesis.* Because we are not attempting to represent ourselves as taxonomic experts for all terrestrial arthropods, we required a published phylogeny, corresponding datasets, and information that could be used to build time calibrated trees. Sometimes the datasets and calibration information were available in the publications’ supplementary materials, but sometimes only parts of datasets were available, or datasets did not correspond to the published trees, in which case we began the analyses from scratch. 2) *Mostly complete sampling of species and distributions.* As discussed in the introduction, the number of species of certain taxa and their distributions are often unknown for terrestrial arthropods. However, in some taxa, we know the true diversity, or at least that there are many unsampled lineages. We chose to omit these datasets from our analyses at this time and hope that they will be subjected to explicit hypothesis testing when more data become available. Datasets we were unable to use because of too little Caribbean or mainland sampling or too many missing taxa were *Newportia* centipedes [96], onychophora [97], *Loxosceles* spiders [81], and *Amphiacusta* crickets [49]. 3) *Genetic differentiation sufficient for phylogenetic analysis.* In the case of *Argiope argentata* spiders [93] and *Dryas iulia* and *Heliconius charithonia* butterflies [46], the taxa lacked enough genetic differentiation to get at the questions we are asking.

Using these criteria, we were left with 10 datasets, some better than others for addressing the hypotheses we wished to test. Our analyses consist of *Platythyrea* ants, *Heterotermes* and *Nasutitermes* termites, butterflies from the genera *Calisto* and *Papilio*, *Drosophila* flies, centruroidine scorpions, and *Micrathena*, *Spintharus*, and *Selenops* spiders. These taxa provide a good representation of sedentary and more vagile taxa with various life histories. When possible, molecular data were supplemented with new data from GenBank (Additional file 1: Tables S5–S15), including both markers and species not available in previous publications. In some cases, the addition of taxa for better calibration precluded the use of certain markers – our strategy was to maximize the sampling effort, including geographic, taxonomic, and phylogenetic coverage. New molecular sequence data were collected only in the case of *Selenops* spiders.

### Phylogenetic and dating analysis

For each dataset (*Platythyrea* ants, *Heterotermes* termites, *Nasutitermes* termites, *Calisto* butterflies, *Papilio* butterflies, *Drosophila* fruit flies, centruroidine scorpions, *Micrathena* spiders, *Spintharus* spiders, and *Selenops* spiders) a phylogeny was estimated in MrBayes [98] (details of each analysis can be found in Additional file 1). Missing data were treated as “?”, and the BIC was used to select the best partitioning scheme and models in PartitionFinder2 [99] and jModelTest2 [100, 101]. Additionally, all data blocks were separated by gene (for non-coding genes) and by codon (for coding genes) for the input files. Further information about each dataset (e.g., genes used, partitioning schemes, models of molecular evolution, generations) can be found Additional file 1. Analyses were run with the default setting of 2 analyses with 4 chains, sampling every 1000 generations. Clades with support values ≥0.95 are considered well-supported. Tracer v. 1.6 [102] was used to examine ESS values to ensure they were > 200 and to examine stationarity plots. All trees were viewed in FigTree v1.4.3 [103]. Each phylogeny was compared to the published phylogeny, then used as a topological constraint for the dating analysis in BEAST 2 [104]. If our results were inconsistent with those previously published or certain relationships were unresolvable, multiple possibilities were tested.

BEAST 2 input files were constructed using BEAUti. Constrained MrBayes molecular phylogenies were dated in BEAST 2 using either fossil calibrations or a molecular clock rate. For information on the dating parameters, please refer to the detailed methods for each taxon in Additional file 1. Burn-in for Tracer 1.6 analyses was 10%, and for trees in TreeAnnotator, it was set to 25% unless otherwise noted. All MrBayes and BEAST 2 analyses were run using XSEDE [105] on the CIPRES Science Gateway [106]. The .xml files and output trees used for subsequent BioGeoBEARS analyses are available on figshare.

### Biogeographic model testing

We conducted biogeographic hypothesis testing and ancestral range reconstruction using the BEAST 2 time-calibrated phylogenies in BioGeoBEARS [107]. The models used (Table 1) to test the biogeographic hypotheses generally follow those of Crews and Gillespie [25] and consider GAARlandia, the various proposed closure dates of the IoP/CAS, and various multipliers of “dispersal ability” in shaping biogeographic histories. The most basic model considered the probability of dispersal without GAARlandia vs. a model that considers GAARlandia. Manual dispersal multiplier matrices were employed to further model the effect of distance and dispersal ability [42] as follows:

- *Distance dependent dispersal*. The probability of reaching a land area decreased as distance between land areas increased. If there was a land connection available during a particular time period, the manual dispersal multiplier (mdm) value was increased (Note: mdm values were only set to 1 in very closely adjacent areas).
- *Distance independent dispersal*. Distance did not play a role in dispersal and establishment probability (presence absence of landmasses only). If there was a hypothesized or known land connection, mdm values were set to 1 (land) and with no connection, set to 0.5. During the proposed timing of GAARlandia, the probability of dispersal from South America to the Greater Antilles increased from 0.5 to 1.
- Vicariance only model. A *very* low probability of dispersal. Land connections remained set to 1, but overwater dispersal was lowered to 0.01. The mdm values from South America to the Greater Antilles during the proposed timing of GAARlandia were increased from 0.01 to 1. When there was no land available for a designated landmass, probabilities were set to 0.0000001 [67].

**Table 1:** Biogeographic models analyzed in BioGeoBEARS. Each dispersal or vicariance scenario was tested using the six models available in BioGeoBEARS (DEC, DEC+J, DIVALIKE, DIVALIKE+J, BAYAREALIKE, BAYAREALIKE+J)

Time Period of Model	Geologic Events Reflected in Model	MODELS 1: Without GAARlandia | 2: With GAARlandia		
		a: Dispersal probability decreases as distance increases	b: Distance does not affect dispersal probability	c: Probability of overwater dispersal is very low
A: 3 my – present	Complete closure of the Central American Seaway and uplift of the Isthmus of Panama [18].	A1a | A2a	A1b | A2b	A1c | A2c
B: 23 my – present	First collision between southern tip of Central America and northwestern South America [19].	B1a | B2a	B1b | B2b	B1c | B2c
C: 15 my – present	Significant land above sea level or complete closure of the Isthmus of Panama [19, 20].	C1a | C2a	C1b | C2b	C1c | C2c
D: 8 my – present	Shoaling of the Central American Seaway from 12 to 7 my [21]; land bridge nearly complete by 10 my [32]. * We chose to use this intermediate period to account for the gradual closure of the Central American Seaway and its uncertainty after 15 my and before 3 my.	D1a | D2a	D1b | D2b	D1c | D2c
E: 23–15 my AND 3 my – present	This model emulates dispersal possibilities during two periods: that of the first contact of South America and Central America, followed by the full closure of the Central American Seaway.	E1a | E2a	E1b | E2b	E1c | E2c
F: 15–8 my AND 3 my – present	This emulates dispersal possibilities during two periods: during a time when there may have been a significant amount of land, followed by the full closure of the Central American Seaway.	F1a | F2a	F1b | F2b	F1c | F2c
G: 8–5 my AND 3 my – present	This emulates dispersal possibilities during two periods: a time when there was shoaling of the Central American Seaway and an increase in land area of the Isthmus of Panama, followed by the full closure of the Central American Seaway.	G1a | G2a	G1b | G2b	G1c | G2c

Finally, we considered various geologic times of taxa moving across the IoP. (See Table 1 and Fig. 2 for details and citations.) All data were analyzed using all models available in the BioGeoBEARS package (DEC, DEC + J, DIVALIKE, DIVALIKE+J, BAYAREALIKE, BAYAREALIKE+J) [107–109]. This resulted in a maximum of 252 models per dataset. The best model was evaluated using the AICc weights [59].

### Model limitations

The Caribbean islands have moved and increased in area and connectedness through time. We acknowledge that these processes are gradual, and geologic dates may not be exact. We’ve therefore elected to examine dispersal vs. vicariance using large generalized areas for both geologically-grouped islands and continents (e.g., “Northern Lesser Antilles” vs. each individual island, “Greater Antilles” vs. each individual island) in spite of potential criticisms of oversimplification (e.g., [110] of [25]). We’ve opted to employ a strategy that avoids over-parameterization while utilizing the data available. We encourage other authors to use the generalized biogeoraphic models we have provided as input files on figshare as a baseline – using different dispersal probabilities, different time periods, different areas, etc. – to test their own hypotheses.

For the time periods and geographic areas used for each analysis, see Tables 1 and 2. We relied on authors and experts for taxa distribution information. While it is possible that taxa may have broader or even narrower distributions, this potentially would not have a large effect on the results because we used broader land areas as our geographic areas for analyses. Geologic dates based on the current published literature for the first appearance of geographic areas guided our manual dispersal multiplier values and are provided in Table 1 and Additional file 1: Table S1.

**Table 2:**
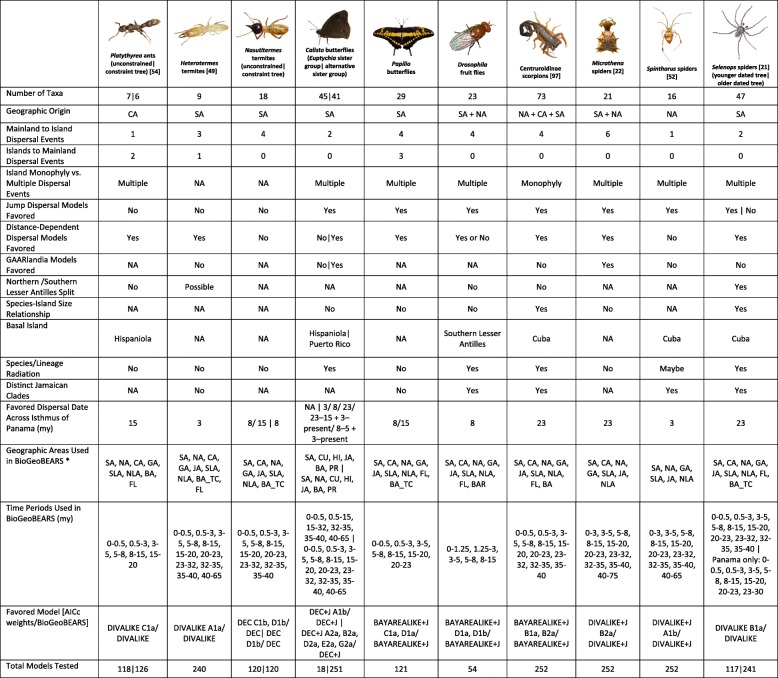
Major biogeographic conclusions and summary of analyses for terrestrial arthropod taxa in the Caribbean biodiversity hotpot. (SA = South America, NA = North America, CA = Central America, GA = Greater Antilles, SLA = Southern Lesser Antilles, NLA = Northern Lesser Antilles, BA = Bahamas and/or Turks and Caicos Islands, FL = Florida, CU = Cuba, HI = Hispaniola, JA=Jamaica, PR = Puerto Rico, BAR=Barbados)

## Supplementary information

**Additional file 1: Table S1.** Geologic dates used in BioGeoBEARS analyses. **Table S2.** Partitions and models used for each dataset. **Table S3.** Summary of untested models for datasets from older taxa. **Table S4.** Summary of untested models for datasets from younger taxa. **Figure S1.** Ancestral range estimation for *Platythyrea* ants. **Table S5.** Sequences for *Platythyrea* analyses. **Table S6.** Bayes Factors from *Platythyrea* analysis. **Figure S2.** Phylogeny and ancestral range estimation for *Heterotermes* termites. **Table S7.** Sequences for *Heterotermes* analyses. **Figure S3.** Phylogeny and ancestral range estimation for *Nasutitermes* termites. **Table S8.** Sequences for *Nasutitermes* analyses. **Figure S4.** Phylogeny and ancestral range estimation for *Calisto* butterflies (*Euptychia* as the outgroup). **Figure S5.** Ancestral range estimation for *Calisto* butterflies (*Euptychia* is not the sister taxon). **Figure S6.** Map of the geographical areas used in the *Calisto* BioGeoBEARS analyses. **Table S9.** *Calisto* butterfly sample information. **Figure S7.** Ancestral range estimation for *Papilio* butterflies. **Table S10.** *Papilio* butterfly sample information **Figure S8.** Ancestral range estimation for *Drosophila* flies. **Table S11.** Sequences for *Drosophila* analyses. **Figure S9.** Ancestral range estimation for centruroidine scorpions. **Table S12.** Sequences for centruroidine scorpion analyses. **Figure S10.** Phylogeny and ancestral range estimation for *Micrathena* spiders. **Table S13.** Sequences for *Micrathena* analyses. **Figure S11.** Ancestral range estimation for *Spintharus* spiders. **Table S14.** Sequences for *Spintharus* analyses. **Figure S12.** Ancestral range estimation for *Selenops* spiders using more recent dated phylogenies. **Figure S13.** Ancestral range estimation for *Selenops* spiders using more recent dated phylogenies. **Figure S14.** Selenopidae tree from the Bayesian analysis using no redundant haplotypes. **Figure S15.** Selenopidae tree from the RAxML analysis using no redundant haplotypes. **Table S15.** Sequences for *Selenops* analyses. **Table S16.** Primers and PCR protocols used for amplification of DNA from *Selenops* species [111–213].

## Data Availability

All data generated or analyzed during this study are included in the additional files, are available on GenBank, or are available as a downloadable package at https://ndownloader.figshare.com/files/18548144.

## Abbreviations

BA: Bahamas and/or Turks and Caicos Islands
BAR: Barbados
CA: Central America
CAS: Central American Seaway
CU: Cuba
FL: Florida
GA: Greater Antilles
GAARlandia: Greater Antilles Aves Ridge
HI: Hispaniola
IoP: Isthmus of Panama
JA: Jamaica
my: Million years
NA: Non-applicable
NA: North America
NLA: Northern Lesser Antilles
PR: Puerto Rico
SA: South America
SLA: Southern Lesser Antilles
