# Supplementary material for: Towards a synthesis of the Caribbean biogeography of terrestrial arthropods
Source: BMC Evol Biol. 2020 Jan 24;20:12. doi: 10.1186/s12862-019-1576-z (PMC6979080; doi:10.1186/s12862-019-1576-z)
Supplement: Supplementary file 1 — Additional file 1: Table S1. Geologic dates used in BioGeoBEARS analyses. Table S2. Partitions and models used for each dataset. Table S3. Summary of untested models for datasets from older taxa. Table S4. Summary of untested models for datasets from younger taxa. Figure S1. Ancestral range estimation for Platythyrea ants. Table S5. Sequences for Platythyrea analyses. Table S6. Bayes Factors from Platythyrea analysis. Figure S2. Phylogeny and ancestral range estimation for Heterotermes termites. Table S7. Sequences for Heterotermes analyses. Figure S3. Phylogeny and ancestral range estimation for Nasutitermes termites. Table S8. Sequences for Nasutitermes analyses. Figure S4. Phylogeny and ancestral range estimation for Calisto butterflies (Euptychia as the outgroup). Figure S5. Ancestral range estimation for Calisto butterflies (Euptychia is not the sister taxon). Figure S6. Map of the geographical areas used in the Calisto BioGeoBEARS analyses. Table S9. Calisto butterfly sample information. Figure S7. Ancestral range estimation for Papilio butterflies. Table S10. Papilio butterfly sample information Figure S8. Ancestral range estimation for Drosophila flies. Table S11. Sequences for Drosophila analyses. Figure S9. Ancestral range estimation for centruroidine scorpions. Table S12. Sequences for centruroidine scorpion analyses. Figure S10. Phylogeny and ancestral range estimation for Micrathena spiders. Table S13. Sequences for Micrathena analyses. Figure S11. Ancestral range estimation for Spintharus spiders. Table S14. Sequences for Spintharus analyses. Figure S12. Ancestral range estimation for Selenops spiders using more recent dated phylogenies. Figure S13. Ancestral range estimation for Selenops spiders using more recent dated phylogenies. Figure S14. Selenopidae tree from the Bayesian analysis using no redundant haplotypes. Figure S15. Selenopidae tree from the RAxML analysis using no redundant haplotypes. Table S15. Sequences for Selenops analyses. Table S1 [file 12862_2019_1576_MOESM1_ESM.docx]

**S1 Table. Geologic dates used in BioGeoBEARS Analyses.** These dates determined our dispersal multiplier matrices and areas allowed matrices in BioGeoBEARS. Note: Aruba, Bonaire, Curaçao, and Trinidad and Tobago were treated as part of the South American continent. Additionally, for these analyses, we consider Central America to be northwestern Panama, Costa Rica, Nicaragua, and southeastern Honduras. North America is considered northern Honduras (The Chortis Block), northward, and North America excludes Florida which is treated as its own geologic entity. The former is due to the Chortis and Maya Blocks sharing a similar history for the time periods that were examined in this paper [9].

| Area | Time | Reference |
| --- | --- | --- |
| Greater Antilles (here refers to Cuba, Hispaniola, Puerto Rico and the Virgin Islands) | No land available before 40 mya | [11, 12, 150] |
| Southern Lesser Antilles (islands from Dominica South) | No land available before 20 mya | [11, 151, 152, 153] |
| Jamaica | No land available before 15 mya* | [11, 154] |
| Northern Lesser Antilles (islands from Guadeloupe north) | No land available before 5 mya | [11, 151, 152, 153] |
| Florida | No land available before 5 mya | [155, 156] |
| Barbados** | No land available before 1.25 mya | [157] |
| Bahamas and Turks and Caicos Islands | No land available before 500 kya | [151, 158, 159, 160] |

*Although it’s possible a portion of western Jamaica was connected to the Chortis Block, it was likely inundated after it was transferred to Jamaica; it is also possible that part of eastern Jamaica (a portion of the Blue Mountains Block) was above sea level, but the evidence is scant at this time.

**Barbados likely underwent 3 stages of emergence and submergence, first emerging 15 mya, then submerged between 8 and 2 mya, then emerging again anywhere from 350-700 kya, though it is unclear whether it was fully submerged after 2 mya. We have chosen to use a median age, 1.25 my.

**S2 Table.** **Information about datasets, partitions, models, and analyses.** The partitioning scheme was used in both MrBayes and BEAST 2 analyses, whereas the models were used only in MrBayes; models used in BEAST 2 are discussed in the text.

| Taxon | Gene(s) | Base pairs | Partitioning Scheme/Models from PartitionFinder2 using the BIC | % missing  (mean, median) | Post-burnin Bayes gens | Post-burnin BEAST gens |
| --- | --- | --- | --- | --- | --- | --- |
| *Platythyrea* ants | COI | 805 | 1. COI pos 1 – GTR + G  2. COI pos 2 - GTR + G  3. COI pos 3 - GTR + G | 1.85, 2.33 | 15 million | 90 million |
| *Heterotermes* termites | 16S | 456 | HKY + G | 5.2, 0 | 22.5 million | 90 million |
| *Nasutitermes* termites | 16S, COII | 1504 | 1. 16S – GTR + G  2. COII pos 1 – GTR + G  3. COII pos 2 – GTR +I + G  4. COII pos 3 – HKY + G | 31.7, 16.1 | 37.5 million | 90 million |
| *Calisto* butterflies | COI, CAD, EF1α, GAPDH, RPS5, WINGLESS | 5297 | 1. CAD pos 3, GAPDH pos 3 - HKY + G  2. CAD pos 1, EF1α pos 1, GAPDH pos 1, RPS5 pos 1, wg pos 1 - GTR +I + G  3. CAD pos 2, COI pos 2, GAPDH pos 2, RPS5 pos 2 - HKY + I + G  4. COI pos 3 – GTR + G  5. COI pos 1 - GTR +I + G  6. EF1α pos 3, RPS5 pos 3 – SYM + I + G  7. EF1α pos 2, wg pos 2 – JC  8. wg pos 3 – GTR + G | 39, 40 | 75 million | See [41] |
| *Papilio* butterflies | COI, COII, EF1α; morphology included in BEAST 2 analysis only | 3542 + 133 morphological characters | 1. COI pos 2, COII pos 2 – F81 + I + G  2. COI pos 3, COII pos 3 – GTR + I + G  3. COI pos 1, tRNA, COII pos 1– GTR + I + G  4. EFIα pos 3 – GTR + G  5. EFIα pos 1, EFIα pos 2 – K80 + I | 27, 20.5 | 75 million | 90 million |
| *Drosophila* flies | COI, COII, 16S-ND1, adh, amd | 4625 | 1. 16S, ND1 pos 1, leu tRNA – GTR +I + G  2. ND1 pos 2, amd pos 2 – K80 + I  3. COII pos 1, COI pos 3, ND1 pos 3 – HKY + G  4. COII pos 2, COI pos 1 – GTR + I  5. COII pos 3, COI pos 2 – F81 + 1  6. adh pos 1, amd pos 3 – GTR + G  7. adh pos 2, adh pos 3 – HKY + I + G  8. amd pos 1 – GTR + G | 24, 22.2 | 75 million | 67.5 million |
| Centruroidinae scorpions | 18S, 16S, 12S, 28S, COI | 4200 | See Esposito and Prendini, 2019 | See Esposito and Prendini, 2019 | See Esposito and Prendini, 2019 | 62.5 million |
| *Micrathena* spiders | COI, 16S, ITS | 2026 | 1. 16S – GTR + I + G  2. ITS – K80 + G  3. COI pos 1 – GTR + I + G  4. COI pos 2 – GTR + I + G  5. COI pos 3 – GTR + G | 26.1, 14.9 | 75 million | 45 million |
| *Spintharus flavidus* spiders | COI, 16S, ITS | 1572 | 1. COI pos 1 – HKY + G  2. COI pos 2 – F81 + G  3. COI pos 3 – GTR + G  4. 16S – GTR + I + G  5. ITS2 0 GTR + G | 26.8, 7.8 | 75 million | 90 million |
| *Selenops* spiders | COI, 16S-ND1, H3 | 2120 | 1. 16S, leu tRNA – GTR + I + G  2. ND1 pos 1 = GTR + G  3. COI pos 1, CO1 pos 2, ND1 pos 2 = GTR + I + G  4. ND1 pos 3 – GTR + G  5. COI pos3 – GTR + I + G  6. H3 pos 1 – JC + I  7. H3 pos 2 – HKY + G  8. H3 pos 3 – SYM + I + G | 17.9, 18.3 | 61,081, 500 | 90 million |

**S3 Table.** Summary of models that would not run (see text) in BioGeoBEARS with taxa old enough (root node >35 my) to test if dispersal was affected by the GAARlandia landspan.

| Taxon | Models that wouldn’t run |
| --- | --- |
| *Heterotermes* termites | DIVALIKE E1a, E1b, E1c, E2a, E2b, E2c, F1a, F1b, F1c, F2a, F2b, F2c |
| *Calisto* butterflies with *Euptychia* as sister taxon (No tests for dispersal across IoP/CAS because no North American or Central American taxa included) | only tested 18 scenarios because of distribution |
| *Calisto* butterflies without *Euptychia* as sister taxon | DEC D1b |
| *Selenops* spiders | DIVALIKE E1a, E1b, E1c, E2a, E2b, E2c, F1a, F1c and DIVALIKE + J E1b, F2a, F2c |

**S4 Table.** Summary of models that would not run in BioGeoBEARS in which taxa are too young (root node < 32 my) to test the role of GAARlandia in dispersal to the Caribbean.

| Taxon | Models that wouldn’t run |
| --- | --- |
| *Platythyrea* ants* | DIVALIKE D1c, BAYAREALIKE D1c, DIVALIKE + J E1a, E1b, E1c, G1a, G1b, G1c |
| *Nasutitermes* termites - analyses using unconstrained tree (see Materials and Methods) | DIVALIKE E1a, E1b, E1c, F1a, F1b, F1c |
| *Nasutitermes* termites – analyses using constrained tree (see Materials and Methods) | DIVALIKE E1a, E1b, E1c, F1a, F1b, F1c |
| *Papilio* butterflies | DIVALIKE E1a, E1b, E1c, F1b, F1c, |
| *Drosophila* fruit flies** | Could only test A, D, G due to young age of taxa examined |
| *Selenops* spiders*** | DIVALIKE E1a, E1b, E1c, f1a, F1b, F1c, DIVALIKE + J E1b, F1b  BAYAREALIKE F1b |

* there were little data available, some missing taxa, and we used bPTP, making an intraspecific analysis interspecific; the second analysis has even fewer taxa than the first.

**Used multiple trees (4) as input for BioGeoBEARS analyses due to instability of some nodes; nearly the same results were obtained for all three analyses.

***Used multiple trees (3) as input for BioGeoBEARS analyses due to instability of some nodes; obtained the same results for all analyses.

**Details of *Platythyrea* Ants Analyses**

Methods

Sequences were downloaded from GenBank (S5 Table). Only Cox1 was used to be able to include outgroups thought to improve the molecular dating calibration. Data were trimmed and aligned by eye using Cox1 codons and checked in Mesquite [110] to ensure there were no stop codons. PartitionFinder2 [104] was used to determine the best partitioning scheme and models of evolution. MrBayes [105] was used to generate a *Platythyrea* tree for use in bPTP [111] for species delimitation. (Note: we did not recover the same results as Seal et al. [54] for the *Platythyrea* species outside of *P. punctata*, however we used different taxa and fewer genes, and the relationships of the *P. punctata* haplotypes remained the same; therefore the differences are seen as inconsequential.) bPTP was run on a server (<http://species.h-its.org/>) using default parameters and was used to delimit lineages of *Platythyrea punctata* to be able to include these ants in our analyses.

Multiple BEAST 2 runs were performed, changing parameters until we felt we obtained reasonable dates based on previous analyses [54, 112] as well as high ESS values, checking the burn-in in Tracer v1.6 [108]. We conducted analyses using multiple outgroups and calibration constraints. We attempted using a molecular clock calibration in addition to fossil and empirical calibration points but found the clock calibration produced unreasonably old dates. The data were partitioned by codon position and BEAST Model Test (BMT) was used. Based on several trial runs, a strict clock model was used for codon position 1 and a relaxed clock log normal model was used for codon positions 2 and 3. We used a Yule model, following Seal et al. [54]. Calibration constraints were: 1) Apoidea, based on Brady et al. [113], consisting of *Apis mellifera* and *Chalybion zimmermani aztecum*, using a normal distribution prior with a mean of 140; 2) An amber fossil calibration as used in Seal et al. [54] consisting of *Platythyrea pilosula*, *P. punctata* BZ6, *P. punctata* HN14, *P. punctata* MX2, *P. punctata* WI9, and *P. strenua*, with a normal distribution prior and a mean of 17.5; 3) a root age of 155, with a sigma value of 15. The relationships of taxa were also constrained based on Schmidt [112] and Seal et al. [54]. Consensus trees were constructed using TreeAnnotator, selecting the maximum clade credibility tree with median heights.

For BioGeoBEARS analyses, outgroup taxa were pruned using Mesquite [110]. Originally, we used 7 taxa in the analyses, eliminating *Platythyrea bicuspus*, the hypothesized sister taxon to *P. prizo*, because it was from Madagascar. Additionally, our results showed *P. pilosula* rather than *P. strenua* as the sister taxon to *P. punctata*. We examined the Bayes Factors associated with these two trees using TreeAnnotator, and although it was higher for the first analysis, the difference was negligible (S6 Table), so we deferred to previous authors [54, 112], constraining *P. strenua* to be the sister taxon to *P. punctata*, and also pruning *P. prizo* from the final tree to be used in BioGeoBEARS.

Detailed Results (Fig 3, S1 Fig)

For *Platythyrea punctata* ants, bPTP [111] recovered 4 putative species from the Cox1 haplotypes that were used in subsequent BEAST 2 and BioGeoBEARS analyses. For this taxon, we only tested models concerning the uplift of the IoP/closure of the CAS (Table 1), given that the age of the root of the clade precluded a scenario including GAARlandia. The best result based on the relative model probabilities of the AICc weights is DIVALIKE C1a (Table 2). This model indicates that dispersal is affected by distance, and a model in which biotic exchange occurred across the IoP/CAS 15 mya is favored. The same model was also identified as the best for the BioGeoBEARS analysis of the unconstrained tree. Ancestral range estimation (Fig 3, S1 Fig) indicates an ancestral range of Central America or the Caribbean, with multiple “reverse” dispersal events to mainland North America. The results also indicate a single dispersal event to the Caribbean.

**S1 Fig. Phylogeny and ancestral range estimation for *Platythyrea* ants**.

BioGeoBEARS phylogram corresponding to the DIVALIKE C1a model (Table 1). Pie charts represent the probabilities of each possible geographic area before and after each split. Colors correspond to Figure 1. SA = South America; CA = Central America; NA = North America; GA = Greater Antilles; SLA = Southern Lesser Antilles; NLA = Northern Lesser Antilles; BA = Bahamas; FL = Florida. Ant photo by Judy Gallagher.­­­­

**S5 Table.** **Sequences for *Platythyrea* analyses.** These ant sequences and outgroups were downloaded from GenBank and used in the BEAST 2 and BioGeoBEARS analyses. All *Platythyrea* sequences were used in the Bayesian analyses, those taxa with an asterisk were used in the BEAST 2 analyses, and those with two asterisks were used in BioGeoBEARS analyses. In one BioGeoBEARS suite of analyses, *P. prizo* isolate 2 was omitted (see text). Distributions used in BioGeoBEARS analyses are provided. (SA=South America; CA=Central America; NA=North America; GA=Greater Antilles; Ja=Jamaica; SLA=Southern Lesser Antilles; NLA=Northern Lesser Antilles; BA=Bahamas and Turks and Caicos Islands; FL=Florida).

­­­
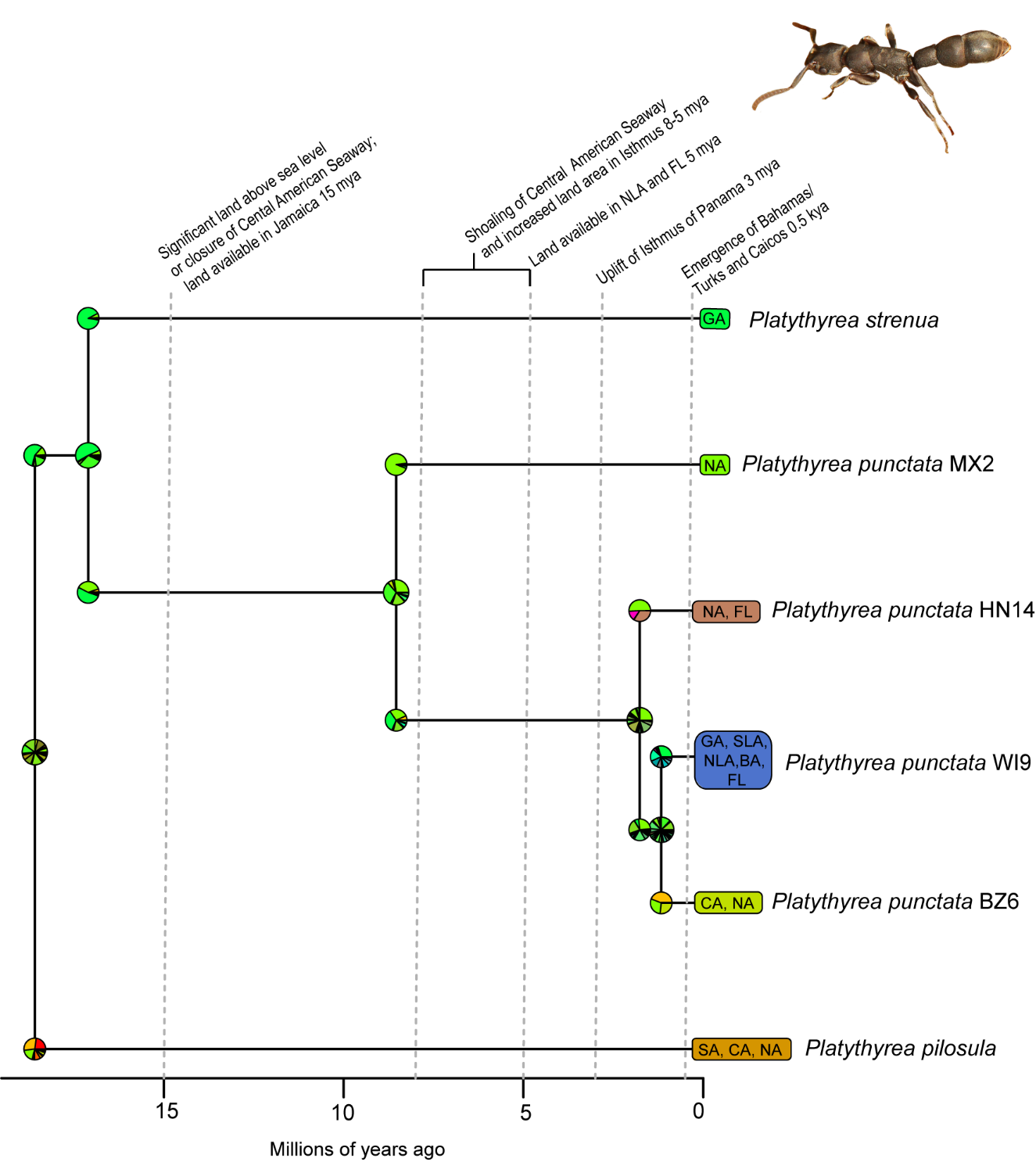


| Species | GenBank Number | Additional File Reference |
| --- | --- | --- |
| *Platythyrea arthuri* voucher CASENT0107704-D01* | DQ176069 | [161] |
| *Platythyrea bicuspus* isolate 1* | HQ440137 | [54] |
| *Platythyrea bicuspus* isolate 2 | HQ440138 | [54] |
| *Platythyrea conradti** | HQ440139 | [54] |
| *Platythyrea parallela** | HQ440140 | [54] |
| *Platythyrea pilosula* UNVERIFIED** ^(SA, CA, NA)^ | HQ440135 | [54] |
| *Platythyrea prizo* isolate 1 | HQ440133 | [54] |
| *Platythyrea prizo* isolate 2** ^(SA, CA, NA)^ | HQ440134 | [54] |
| *Platythyrea punctata* haplotype BZ-1 | HQ440150 | [54] |
| *Platythyrea punctata* haplotype BZ-2 | HQ440151 | [54] |
| *Platythyrea punctata* haplotype BZ-3 | HQ440152 | [54] |
| *Platythyrea punctata* haplotype BZ-4 | HQ440153 | [54] |
| *Platythyrea punctata* haplotype BZ-5 | HQ440154 | [54] |
| *Platythyrea punctata* haplotype BZ-6** ^(CA, NA)^ | HQ440155 | [54] |
| *Platythyrea punctata* haplotype CR1 | HQ440173 | [54] |
| *Platythyrea punctata* haplotype HN-1 | HQ440161 | [54] |
| *Platythyrea punctata* haplotype HN-2 | HQ440162 | [54] |
| *Platythyrea punctata* haplotype HN-3 | HQ440163 | [54] |
| *Platythyrea punctata* haplotype HN-4 | HQ440164 | [54] |
| *Platythyrea punctata* haplotype HN-5 | HQ440132 | [54] |
| *Platythyrea punctata* haplotype HN-6 | HQ440166 | [54] |
| *Platythyrea punctata* haplotype HN-7 | HQ440167 | [54] |
| *Platythyrea punctata* haplotype HN-8 | HQ440168 | [54] |
| *Platythyrea punctata* haplotype HN-14** ^(NA, FL)^ | HQ440170 | [54] |
| *Platythyrea punctata* haplotype MX-1 | HQ440171 | [54] |
| *Platythyrea punctata* haplotype MX-2** ^(NA)^ | HQ440172 | [54] |
| *Platythyrea punctata* haplotype TX-1 | HQ440156 | [54] |
| *Platythyrea punctata* haplotype TX-2 | HQ440157 | [54] |
| *Platythyrea punctata* haplotype TX-3 | HQ440158 | [54] |
| *Platythyrea punctata* haplotype TX-4 | HQ440159 | [54] |
| *Platythyrea punctata* haplotype TX-5 | HQ440160 | [54] |
| *Platythyrea punctata* haplotype WI-1 | HQ440142 | [54] |
| *Platythyrea punctata* haplotype WI-2 | HQ440141 | [54] |
| *Platythyrea punctata* haplotype WI-3 | HQ440143 | [54] |
| *Platythyrea punctata* haplotype WI-4 | HQ440144 | [54] |
| *Platythyrea punctata* haplotype WI-5 | HQ440145 | [54] |
| *Platythyrea punctata* haplotype WI-6 | HQ440146 | [54] |
| *Platythyrea punctata* haplotype WI-7 | HQ440147 | [54] |
| *Platythyrea punctata* haplotype WI-8 | HQ440148 | [54] |
| *Platythyrea punctata* haplotype WI-9** ^(GA, SLA, NLA, FL, BA)^ | HQ440149 | [54] |
| *Platythyrea punctata* voucher RA0250 | DQ353357 | [162] |
| *Platythyrea strenua*** ^(GA)^ | EU155441 | [163] |
| *Apis mellifera macedonica* isolate maced3* | AY114474 | [164] |
| *Chalybion zimmermanni aztecum** | JF927335 | [165] |
| *Paraponera clavata* CS0328* | DQ353276 | [162] |
| *Tatuidris tatusia* voucher RA0399* | DQ353314 | [162] |
| *Cephalonomia stephanoderis** | GQ374632 | [166] |

**S6 Table.** **Bayes Factors from *Platythyrea* analysis.** Models compared by marginal likelihood (S.E. estimated from bootstrap replicates) in Tree Annotator v1.6. Differences between log marginal likelihoods (specifically, log Bayes factors) are reported. Positive values indicate a better relative model fit of the row’s model compared to the column’s model.

| Analysis | Ln P(data \| model) | S.E. | *P. pilosula* + *P. punctata* | *P. strenua* + *P. punctata* constraint |
| --- | --- | --- | --- | --- |
| *Platythyrea pilosula* + *Platythyrea punctata* | -5240.112 | +/- 0.141 | - | 0.83 |
| *Platythyrea strenua + Platythyrea punctata* constraint | -5240.942 | +/- 0.156 | -0.83 | - |

**Details of *Heterotermes* Termites Analyses**

Methods

Sequences were downloaded from GenBank (S7 Table). The 16S gene was used in order to include the most ingroup and outgroup taxa. Data were trimmed in Mesquite [110] and aligned in MAFFT vs. 7 [114] on a server (<http://mafft.cbrc.jp/alignment/software/>) using default settings. jModelTest2 [106, 107] was used to determine the best model of evolution using the BIC. MrBayes [105] was used to generate a tree to compare with BEAST 2 results to ensure the dating results were not derived from getting stuck on local optima. Tracer v1.6 [108] was used to examine convergence of the Bayesian analysis.

BEAST 2 ru­ns were performed [97], and Tracer v1.6 was used to examine plots and ESS values to assess runs. We used BMT, a Relaxed Clock Log Normal model, and a Yule model of speciation. The same partitioning scheme was used in these analyses as in the MrBayes analysis. We used outgroup taxa based on Ware et al. [115] to aid in tree calibration. Calibration constraints were: 1) a Dictyoptera Root Node, using a normal distribution with a mean of 250, and a sigma of 30; 2) Termitidae, which includes *Heterotermes*, *Nasutitermes*, *Cyrilliotermes*, *Macrotermes*, and *Silvestretermes*, using a uniform prior, with a lower bound of 18 and an upper bound of 140; 3) Neoisoptera, which includes, *Heterotermes*, *Nasutitermes*, *Cyrilliotermes*, *Dolichorhinotermes*, *Macrotermes*, *Rhinotermes*, and *Silvestretermes*, using a uniform prior, with a lower bound of 100 and an upper bound of 140; 4) *Kalotermes*, including *Kalotermes flavicollis* and *Cryptotermes*, using a log normal prior, M= 20, S=0.75, Offset=100; 5) Isoptera, including everything except the roaches and mantids, with a log normal prior, M=60, S=0.5, Offset =140; 6) Euisoptera, which includes everything except the roaches, mantids and *Mastotermes*, with a log normal prior, M=20, S=0.9, Offset=140. Consensus trees were produced using TreeAnnotator, selecting the maximum clade credibility tree with median heights to use in subsequent analyses. Outgroup taxa were pruned in Mesquite, leaving only *Heterotermes* species for BioGeoBEARS analyses. Because the *Heterotermes* root age was older than the proposed existence of GAARlandia, we tested all 252 models.

Results (S2 Fig, Fig 4)

Although there were some differences between the Szalanski et al. [49] phylogeny and our *Heterotermes* phylogeny, no differences were well-supported. Some discrepancies are expected given that the former was analyzed with parsimony, and we analyzed our tree using Bayesian criteria. For subsequent analyses, we relied on the trees produced by MrBayes and BEAST 2, as these were congruent with one another. The best model based on the relative probabilities from the AICc weights is DIVALIKE A1a (Tables 1, 2), or a DIVALIKE model with the following constraints: distance between land areas affects dispersal, GAARlandia did not play a role in dispersal from South America to the Greater Antilles, and dispersal across the IoP/CAS occurred from 3 mya. Ancestral range estimation (S2 Fig, Fig 4) indicates 3 separate dispersals into the Caribbean, 2 from South America (*Heterotermes convexinotatus* and *H. tenuis*) and 1 from North America.

**S2 Fig. Phylogeny and ancestral range estimation for *Heterotermes* termites.**

BioGeoBEARS phylogram corresponding to the DIVALIKE A1a model (Table 1). Pie charts in the tree represent the probabilities of each possible geographical area before and after each split. Colors correspond to Figure 1. SA = South America; CA = Central America; NA = North America; GA = Greater Antilles; JA = Jamaica; SLA = Southern Lesser Antilles; NLA = Northern Lesser Antilles; TCI = Turks and Caicos Islands; FL = Florida. *Heterotermes* photo provided by Rudolf Scheffrahn.


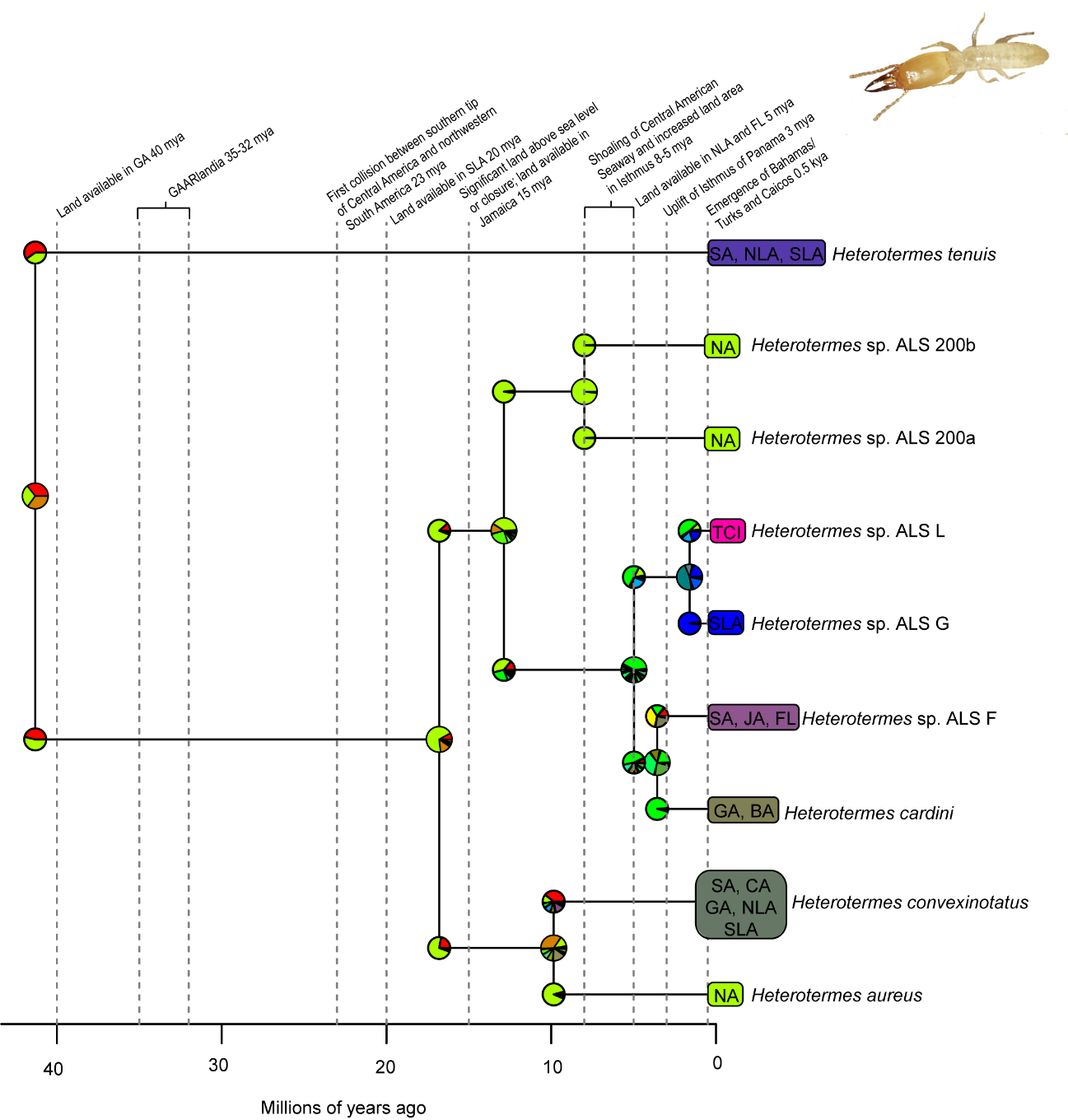


**S7 Table.** **Sequences for *Heterotermes* analyses.** These termite sequences and outgroups were downloaded from GenBank and used in the BEAST 2 and BioGeoBEARS analyses. All *Heterotermes* sequences were used in the Bayesian analyses along with *Nasutitermes costalis* and *Kalotermes flavicollis* as ougtroups, and those taxa with at least one asterisk were used in the BEAST 2 analyses, and those with two asterisks were also used in BioGeoBEARS analyses. Distributions considered for each species used in the BioGeoBEARS analysis are provided. (SA=South America; CA=Central America; NA=North America; GA=Greater Antilles; JA=Jamaica; SLA=Southern Lesser Antilles; NLA=Northern Lesser Antilles; BA=Bahamas and Turks and Caicos Islands; FL=Florida)

| Species | GenBank Number | Additional File Reference |
| --- | --- | --- |
| *Heterotermes aureus*** ^(NA)^ | AY380299 | [49] |
| *Heterotermes cardini* from Bahamas, Great Exuma | AY380256 | [49] |
| *Heterotermes cardini* from Bahamas, South Andros | AY380257 | [49] |
| *Heterotermes cardini* from Bahamas, Long Island | AY380258 | [49] |
| *Heterotermes cardini* from Bahamas, Great Exuma** ^(GA, BA)^ | AY380259 | [49] |
| *Heterotermes cardini* from Bahamas, Cat Island | AY380260 | [49] |
| *Heterotermes cardini* from Bahamas, Crooked Island | AY380261 | [49] |
| *Heterotermes cardini* from Bahamas, Cat Island | AY380262 | [49] |
| *Heterotermes cardini* from Bahamas, North Andros | AY380263 | [49] |
| *Heterotermes cardini* from Bahamas, North Cat Cay | AY380264 | [49] |
| *Heterotermes cardini* from Bahamas, Eleuthera | AY380265 | [49] |
| *Heterotermes cardini* from Bahamas, Acklins Island | AY380266 | [49] |
| *Heterotermes cardini* from Bahamas, Exuma, Barreterre | AY380267 | [49] |
| *Heterotermes cardini* from Bahamas, Little Exuma | AY380268 | [49] |
| *Heterotermes cardini* from Bahamas, North Bimini | AY380269 | [49] |
| *Heterotermes cardini* from Bahamas, South Bimini | AY380270 | [49] |
| *Heterotermes cardini* from Bahamas, New Providence | AY380271 | [49] |
| *Heterotermes cardini* from Cuba | AY380272 | [49] |
| *Heterotermes cardini* from Bahamas, San Salvador | AY380273 | [49] |
| *Heterotermes cardini* from Bahamas, Rum Cay | AY380274 | [49] |
| *Heterotermes cardini* from Bahamas, Mangrove Cay | AY380275 | [49] |
| *Heterotermes convexinotatus* from Fajardo, Puerto Rico** ^(SA, CA, GA, SLA, NLA)^ | AY695113 | [167] |
| *Heterotermes convexinotatus* from Puerto Nuevo, Puerto Rico | AY695120 | [167] |
| *Heterotermes convexinotatus* from Vega Alta, Puerto Rico | AY695121 | [167] |
| *Heterotermes convexinotatus* from Tres Hermanos, Puerto Rico | AY695123 | [167] |
| *Heterotermes convexinotatus* from east of Combate Beach, Puerto Rico | AY695125 | [167] |
| *Heterotermes convexinotatus* from west of Manuabo, Puerto Rico | AY695129 | [167] |
| *Heterotermes convexinotatus* from Punta Salinas, Puerto Rico | AY695131 | [167] |
| *Heterotermes convexinotatus* from Antigua and Barbuda | AY380277 | [49] |
| *Heterotermes convexinotatus* from St. Maarten | AY380278 | [49] |
| *Heterotermes convexinotatus* from Bani, Dominican Republic | AY380279 | [49] |
| *Heterotermes convexinotatus* from St. Kitts and Nevis | AY380280 | [49] |
| *Heterotermes convexinotatus* from St. Eustatius | AY380281 | [49] |
| *Heterotermes convexinotatus* from Barbados | AY380282 | [49] |
| *Heterotermes convexinotatus* from Santo Domingo, Dominican Republic | AY380283 | [49] |
| *Heterotermes convexinotatus* from Martinique | AY380284 | [49] |
| *Heterotermes convexinotatus* from Aruba | AY380285 | [49] |
| *Heterotermes convexinotatus* from Curaçao | AY380286 | [49] |
| *Heterotermes convexinotatus* from Vieques, Puerto Rico | AY380287 | [49] |
| *Heterotermes convexinotatus* from Culebra, Puerto Rico | AY380288 | [49] |
| *Heterotermes convexinotatus* from St. Croix, Virgin Islands | AY380289 | [49] |
| *Heterotermes convexinotatus* from St. Thomas, Virgin Islands | AY380290 | [49] |
| *Heterotermes convexinotatus* from Buck Island, Virgin Islands | AY380291 | [49] |
| *Heterotermes convexinotatus* from Trinidad and Tobago | AY380292 | [49] |
| *Heterotermes convexinotatus* from Indian Castle, St. Kitts and Nevis | AY380293 | [49] |
| *Heterotermes convexinotatus* from St. John, Virgin Islands | AY380294 | [49] |
| *Heterotermes convexinotatus* from Humacao, Puerto Rico | AY380295 | [49] |
| *Heterotermes convexinotatus* from Anguilla | AY380296 | [49] |
| *Heterotermes convexinotatus* from Great Inagua, Bahamas | AY380297 | [49] |
| *Heterotermes convexinotatus* | AY380298 | [49] |
| *Heterotermes tenuis* | HQ683708 | [168] |
| *Heterotermes tenuis* | AY553179 | [169] |
| *Heterotermes tenuis* from Grenada** ^(SA, SLA, NLA)^ | AY695131 | [49] |
| *Heterotermes tenuis* from St. Vincent | AY380239 | [49] |
| *Heterotermes tenuis* from St. Lucia | AY380240 | [49] |
| *Heterotermes tenuis* from Trinidad and Tobago | AY380241 | [49] |
| *Heterotermes tenuis* from Trinidad and Tobago | AY380242 | [49] |
| *Heterotermes tenuis* from Trinidad and Tobago | AY380243 | [49] |
| *Heterotermes tenuis* from Trinidad and Tobago | AY380244 | [49] |
| *Heterotermes tenuis* from Guadeloupe | AY380245 | [49] |
| *Heterotermes tenuis* from Guadeloupe | AY380246 | [49] |
| *Heterotermes tenuis* from Guadeloupe | AY380247 | [49] |
| *Heterotermes tenuis* | AY380248 | [49] |
| *Heterotermes* sp. ALS-D from Cayman Islands | AY380249 | [49] |
| *Heterotermes* sp. ALS-D from Bonaire | AY380250 | [49] |
| *Heterotermes* sp. ALS-E from Mahoe Bay, Jamaica | AY380252 | [49] |
| *Heterotermes* sp. ALS-E from Negril, Jamaica | AY380253 | [49] |
| *Heterotermes* sp. ALS-F from Dade Co., Florida** ^(SA, JA, FL)^ | AY380254 | [49] |
| *Heterotermes* ALS-G, St. Barthelemy** ^(NLA)^ | AY380255 | [49] |
| *Heterotermes* ALS-L, Turks and Caicos Islands, Grand Turk** ^(BA)^ | AY380276 | [49] |
| *Heterotermes* sp. ALS-2003a Veracruz, Mexico** ^(NA)^ | AY380300 | [49] |
| *Heterotermes* sp. ALS-2003b Xunatunich Ruins, Belize** ^(NA)^ | AY380301 | [49] |
| *Cryptotermes colombianus* isolate COPT056* | KX267099 | [170] |
| *Cyrilliotermes* sp. VR-2013* | KF724764 | [117] |
| *Dolichorhinotermes* sp. ‘Manaus’* | AF262582 | [169] |
| *Kalotermes flavicollis** | AY486437 | [49] |
| *Macrotermes subhyalinus* isolate MS3* | JQ429115 | [171] |
| *Mastotermes darwiniensis* voucher ZMUC: JD-861* | JN615266 | [172] |
| *Microhodotermes viator* voucher BYU IGC IS17* | EU253741 | [173] |
| *Nasutitermes costalis* Broward Co., FL* | AY486438 | [49] |
| *Rhinotermes marginalis* voucher BYU IGC IS61* | EU253754 | [173] |
| *Rhinotermes* sp. ‘Manaus’* | AF262581 | [169] |
| *Silvestretermes* sp. VR-2013* | KF724763 | [117] |
| *Stolotermes ruficeps* strain SR1* | KF840412 | [174] |
| *Cryptocercus kyebangensis** | KF855853 | [172] |
| *Periplaneta americana** | U17806 | [175] |
| *Periplaneta australasiae* voucher ZMUC: JD-883* | JN615292 | [172] |
| *Periplaneta fuliginosa** | PFU17812 | [175] |
| *Cheddikulama straminea* voucher YDWC79* | KT316263 | [176] |
| *Heterochaetula tricolor* voucher YDWC101* | KT316261 | [176] |
| *Heterochaetula* sp. CL-2015* | KT316262 | [176] |

**Details of *Nasutitermes* Termites Analysis**

Methods

Sequences were downloaded from GenBank (S8 Table). The genes 16S and CoxII allowed us to maximize both taxonomic and molecular sampling. The data were concatenated in SequenceMatrix v1.8 [116]. The 16S data were aligned in MAFFT [114] on a server (<http://mafft.cbrc.jp/alignment/software/>) using default settings. MrBayes [105] was used to generate a tree to compare to the results of previous studies [68, 117] as well as to the BEAST 2 results. Tracer v1.6 was used to examine convergence of both the Bayesian and BEAST 2 analyses.

The BEAST 2 analysis was partitioned the same as the MrBayes analyses. We used BMT, a relaxed clock log normal model, and a Yule model of speciation. However, two of the partitions (1 and 4) had low ESS values for BMT, so we used HKY + G for these two partitions, which increased the ESS values >200. We used outgroup taxa based on Ware et al. [115] to aid in node calibration. Calibration constraints were: 1) Dictyoptera Root Node, with a normal distribution, a mean of 250 and sigma 30. 2) Termitidae, which includes *Heterotermes*, *Nasutitermes*, *Cyrilliotermes*, *Macrotermes*, and *Silvestretermes*, using a uniform prior, with a lower bound of 18 and an upper bound of 140. 3) Neoisoptera, which includes, *Heterotermes*, *Nasutitermes*, *Cyrilliotermes*, *Dolichorhinotermes*, *Macrotermes*, *Rhinotermes*, and *Silvestretermes*, using a uniform prior, with a lower bound of 100 and an upper bound of 140. 4) Isoptera, including everything except the roaches and mantids, with a log normal prior, M=60, S=0.5, Offset =140. 5) Euisoptera, which includes everything except the roaches, mantids and *Mastotermes*, with a log normal prior, M=20, S=0.9, Offset=140. Consensus trees were produced using TreeAnnotator, selecting the maximum clade credibility tree with median heights to use in subsequent analyses. Outgroup taxa were pruned in Mesquite, leaving only *Nasutitermes* for BioGeoBEARS analysis. Because the *Nasutitermes* root age was younger (~30 my) than the proposed existence of GAARlandia, we were only able to test hypotheses involving the closure of the CAS. Due to differences between the Bayesian tree (even when polytomies were resolved using SumTrees [118, 119]), the BEAST 2 tree, and the tree from Roy et al. [117], we ran a BEAST 2 analysis using constraints to produce a tree more similar (different taxa were used in both analyses, so we cannot produce the same tree) to Roy et al. [117] to use in an additional BioGeoBEARS analysis. This analysis was run exactly as the other, but also constraining these taxa: *N. acangussu*, *N. acajutlae*, *N. rippertii*, and *N. surinamensis*.

Results (S3 Fig, Fig 5)

*Nasutitermes* gene trees from our MrBayes analyses, BEAST 2 analyses, and Roy et al. [117] have similar, yet not identical, topologies. This is expected due to the different taxa and genes used in this study and that of Roy et al. [117]. It should be noted, however, that in all analyses *N. intermedius* is firmly nested within *Nasutitermes*, which is not congruent with Cuezzo et al.’s [144] transfer to the genus *Cortaritermes*. Because of the slight differences in the resultant trees, we constrained taxa in BEAST 2 to produce a tree closer to that of Roy et al. [117] and also conducted an additional suite of BioGeoBEARS analyses using the constrained phylogeny as our input tree. An analysis of the Bayes Factors performed in Tracer v1.6 [108] indicate that the Roy et al. [117] tree does not have a relative better fit than the tree we generated. For the BioGeoBEARS analyses using the unconstrained tree, two models were equally favored according to the AICc weights: DEC C1b and DEC D1b (Tables 1, 2). These models represent a DEC model, with the following constraints: distance between land areas has no effect on dispersal, and dispersal began across the IoP/CAS at either 15 or 8 mya, earlier than the closure of the seaway. Ancestral range estimation for C1a indicates either a Caribbean or South American origin. The BioGeoBEARS analyses using the Roy et al. [117] tree indicate that the best fit model was also DEC D1b (Tables 1,2). Ancestral range estimation indicates multiple dispersal events into the Caribbean from South America (S3 Fig, Fig 5).

**S3 Fig. Phylogeny and ancestral range estimation for *Nasutitermes* termites**.

BioGeoBEARS phylogram corresponding to the DEC D1b model (Table 1). Pie charts represent the probabilities of each possible geographical area before and after each split. Colors correspond to Figure 1. SA = South America; CA = Central America; NA = North America; GA = Greater Antilles; JA = Jamaica; SLA = Southern Lesser Antilles; NLA = Northern Lesser Antilles; BA = Bahamas. *Nasutitermes* photo by Bernard Dupont.


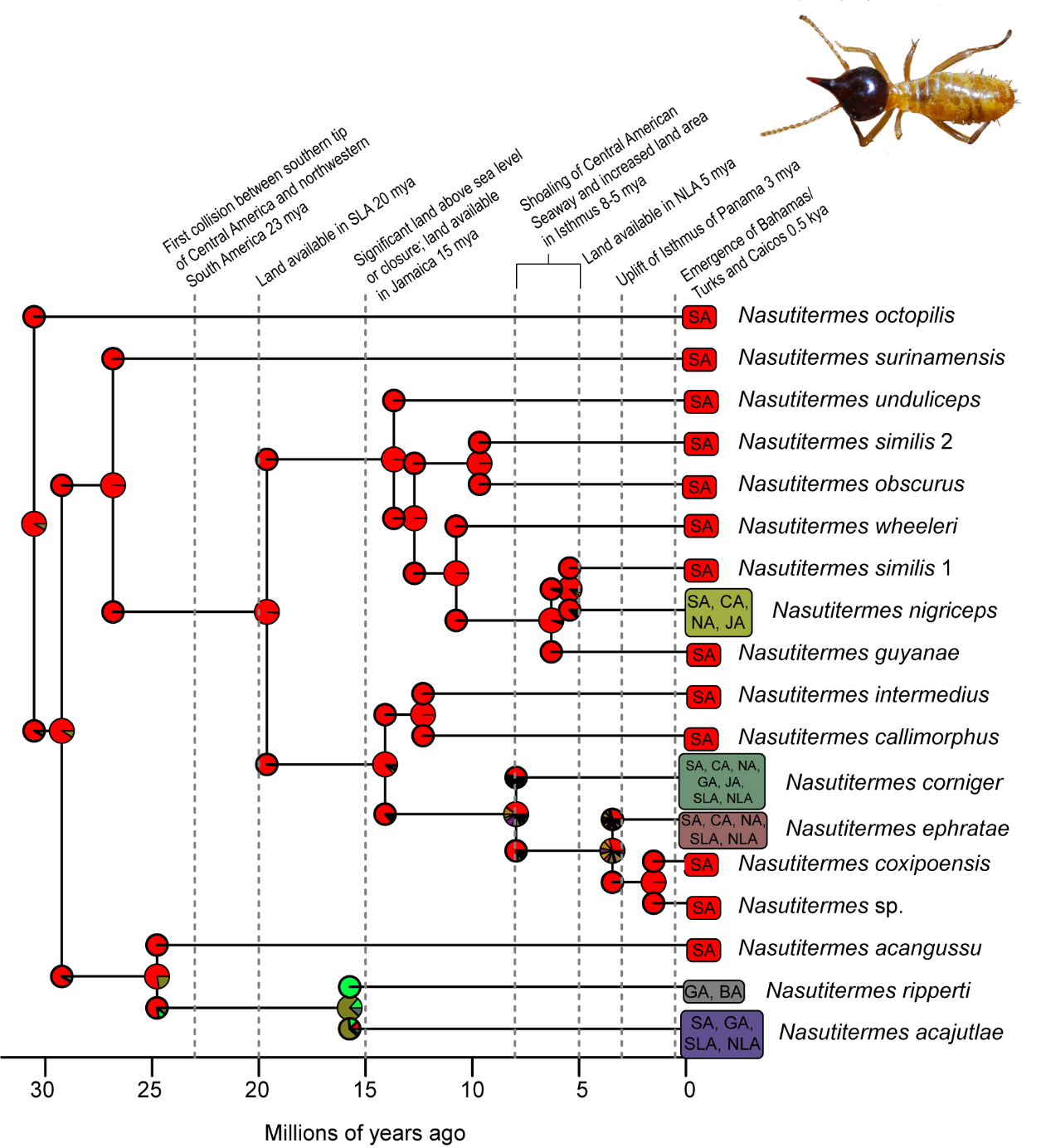


**S8 Table.** **Sequences for *Nasutitermes* analyses.** These termite sequences and outgroups were downloaded from GenBank and used in the BEAST 2 and BioGeoBEARS analyses. All *Nasutitermes* sequences were used in the Bayesian analyses along with ougtroups, and those taxa with at least one asterisk were used in the BEAST 2 analyses, and those with two asterisks were also used in BioGeoBEARS analyses. Distributions considered for each species used in the BioGeoBEARS analyses are provided. (SA=South America; CA=Central America; NA=North America; GA=Greater Antilles; Ja=Jamaica; SLA=Southern Lesser Antilles; NLA=Northern Lesser Antilles; BA=Bahamas and Turks and Caicos Islands; FL=Florida) Note: some outgroup sequences are chimeric (the 16S and CO2 sequences are not from the same individuals) to be able to include more data.

| Species | COII | 16S | Additional File Reference |
| --- | --- | --- | --- |
| *Nasutitermes acajutlae* isolate CAC1 Hap15.1 ** ^(SA, GA, SLA, NLA)^ | KC631019 | KF724760 | [117] |
| *Nasutitermes acajutlae* isolate IDS4 Hap15.2 | KC631020 | KF724761 | [117] |
| *Nasutitermes acajutlae* isolate IDS6 Hap15.3 | KC631021 | KF724762 | [117] |
| *Nasutitermes acangussu* isolate ELA1 Hap14.1 ** ^(SA)^ | KC631017 | KF724759 | [117] |
| *Nasutitermes acangussu* isolate PAT1 Hap14.2 | KC631018 | - | [117] |
| *Nasutitermes callimorphus* isolate NOU25 Hap4.1 ** ^(SA)^ | KC631001 | KF724742 | [117] |
| *Nasutitermes corniger* isolate AWA1 Hap1.1 ** ^(SA, CA, NA< GA, Ja, SLA, NLA)^ | KC630989 | KF724731 | [117] |
| *Nasutitermes corniger* isolate COU1 Hap1.2 | KC630990 | KF724732 | [117] |
| *Nasutitermes corniger* isolate ELA2 Hap1.3 | KC630991 | KF724733 | [117] |
| *Nasutitermes costalis* | DQ530632 | - | [177] |
| *Nasutitermes costalis* | - | AY486438 | [178] |
| *Nasutitermes costalis* from Dominica | - | AY623093 | [178] |
| *Nasutitermes costalis* from Dominican Republic | - | AY623100 | [178] |
| *Nasutitermes costalis* from Ecuador | - | AY623085 | [178] |
| *Nasutitermes costalis* from Guadeloupe | - | AY623099 | [178] |
| *Nasutitermes costalis* from Jamaica | - | AY623096 | [178] |
| *Nasutitermes costalis* from Mexico | - | AY623094 | [178] |
| *Nasutitermes costalis* Puerto Rico | - | AY623098 | [178] |
| *Nasutitermes costalis* from St. Kitts | - | AY623097 | [178] |
| *Nasutitermes costalis* from Suriname | - | AY623095 | [178] |
| *Nasutitermes coxiepoensis* isolate MDY1 Hap2b.1 ** ^(SA)^ | KC630994 | KF724737 | [117] |
| *Nasutitermes ephratae* isolate BEL4 Hap3.1 ** ^(SA, CA, NA, SLA, NLA)^ | KC630995 | KF724738 | [117] |
| *Nasutitermes ephratae* isolate NIS2 Hap3.2 | KC630996 | KF724739 | [117] |
| *Nasutitermes ephratae* isolate PAT2 Hap3.3 | KC630997 | KF724740 | [117] |
| *Nasutitermes ephratae* isolate PAT3 Hap3.4 | KC630998 | KF724741 | [117] |
| *Nasutitermes guayanae* isolate NOU6 Hap7.1 ** ^(SA)^ | KC631005 | KF724748 | [117] |
| *Nasutitermes intermedius* isolate BEL1 Hap5.1 ** ^(SA)^ | KC631002 | KF724743 | [117] |
| *Nasutitermes nigriceps* ** ^(SA, VA, NA, Ja)^ | AB037329 | AB037346 | [179] |
| *Nasutitermes obscurus* isolate NOU7 Hap9.1 ** ^(SA)^ | KC631008 | KF724754 | [117] |
| *Nasutitermes octopilis* isolate NOU27 Hap12.1 ** ^(SA)^ | KC631012 | KF724757 | [117] |
| *Nasutitermes octopilis* isolate NOU14 Hap12.2 | KC631013 | - | [117] |
| *Nasutitermes rippertii* ** ^(GA, BA)^ | - | AY623091 | [178] |
| *Nasutitermes similis* isolate BEL5 Hap6.1 ** ^(SA)^ | KC631003 | KF724744 | [117] |
| *Nasutitermes similis* isolate CAC3 Hap6.2 | KC631004 | KF724745 | [117] |
| *Nasutitermes similis* Hap6.3 | - | KF724746 | [117] |
| *Nasutitermes similis* Hap6.4 | - | KF724747 | [117] |
| *Nasutitermes similis* isolate NOU28 Hap8.1 ** ^(SA)^ | KC631006 | KF724749 | [117] |
| *Nasutitermes similis* NOU20 Hap8.2 | KC631007 | KF724750 | [117] |
| *Nasutitermes similis* Hap8.3 | - | KF724751 | [117] |
| *Nasutitermes similis* Hap8.4 | - | KF724752 | [117] |
| *Nasutitermes similis* Hap8.5 | - | KF724753 | [117] |
| *Nasutitermes surinamensis* isolate AWA2 Hap13.1 ** ^(SA)^ | KC631014 | KF724758 | [117] |
| *Nasutitermes surinamensis* isolate MAT3 Hap13.2 | KC631015 | - | [117] |
| *Nasutitermes surinamensis* isolate SAU2 Hap13.3 | KC631016 | - | [117] |
| *Nasutitermes unduliceps* isolate PAT8 Hap10.1 ** ^(SA)^ | KC631009 | KF724755 | [117] |
| *Nasutitermes* sp. Hap2a.1 ** ^(SA)^ | KC630992 | KF724735 | [117] |
| *Nasutitermes* sp. Hap2a.2 | KC630993 | KF724736 | [117] |
| *Nasutitermes wheeleri* isolate COU3 Hap11.1 ** ^(SA)^ | KC631010 | KF724756 | [117] |
| *Nasutitermes wheeleri* isolate NIS4 Hap11.2 | KF724756 | - | [117] |
| *Cryptotermes colombianus* isolate COPT056* | KU510330 | KX267099 | [170] |
| *Cyrilliotermes* sp. VR-2013* | KC631024 | KF724764 | [171] |
| *Dolichorhinotermes* sp. ‘Manaus’* | AF262601 | AF262582 | [169] |
| *Macrotermes subhyalinus* isolate MS3* | KT845958 | JQ429115 | [172, 180] |
| *Mastotermes darwiniensis* voucher ZMUC: JD-861* | FJ806882 | JN615266 | [172, 181] |
| *Microhodotermes viator* voucher BYU IGC IS17* | EU253876 | EU253741 | [173] |
| *Rhinotermes marginalis* voucher BYU IGC IS61* | EU253890 | EU253754 | [173] |
| *Rhinotermes* sp. ‘Manaus’* | AF262600 | AF262581 | [169] |
| *Silvestritermes* sp. VR-2013* | KC631023 | KF724763 | [117] |
| *Stolotermes ruficeps* strain SR1* | - | KF840412 | [174] |
| *Cryptocercus kyebangensis** | KF855905 | KF855853 | [172] |
| *Periplaneta americana** | EF363225 | U17806 | [175, 182] |
| *Periplaneta australasiae* voucher ZMUC: JD-883* | DQ442219 | JN615292 | [172. 183] |
| *Periplaneta fuliginosa** | DQ874312 | PFU17812 | [175, 183] |
| *Cheddikulama straminea* voucher YDWC79* | KT316267 | KT316263 | [176] |
| *Heterochaetula tricolor* voucher YDWC101* | - | KT316261 | [176] |
| *Heterochaetula* sp. CL-2015* | KT316266 | KT316262 | [176] |

**Details of *Calisto* Butterflies Analyses**

Methods

We downloaded the matrices available from Matos-Maraví et al. [23] for both the MrBayes and BEAST 2 analysis. PartitionFinder2 [104] was used to choose the partitioning scheme and models of evolution (S2, S9 Tables). In addition to the matrices available for download, trees were also available, including one Bayesian tree; however, this tree lacked support values and had fewer taxa than the published BEAST analysis, so we re-ran the MrBayes analysis to ensure we obtained the same results as Matos-Maraví et al. [23]. The authors conducted two BEAST analyses, one that included the outgroup *Euptychia* and one that did not, as the latter supposedly caused long branch attraction issues in a previous analysis [120]. Matos-Maraví et al. [23] did not corroborate this issue, but we decided to use the trees from both analyses for subsequent BioGeoBEARS analyses. There was some confusion regarding the placement of *Calisto nubila*. This species is always basal in the trees shown in Matos-Maraví et al. [23], but in the trees available on TreeBase that include *Euptychia*, it is not basal, but rather nested well-within the tree and *Calisto obscura* is basal instead. We assume the authors chose to use the tree in which *C. nubila* is basal based on the Bayesian analyses or for other reasons. We recovered this relationship as strongly supported in our Bayesian analysis and assume the authors are familiar with their taxon of choice, so we used a tree in which *C. nubila* is basal for the BioGeoBEARS analysis in which *Euptychia* is not the sister taxon to *Calisto*. Because *Calisto* and its sister taxa have a different distribution than the other taxa used in this paper, the areas allowed in BioGeoBEARS differed from our other analyses (S6 Fig).

In the BioGeoBEARS analysis in which *Euptychia* is sister to *Calisto*, taxa are only from South America (*Euptychia*), the Greater Antilles, and Bahamas, with no taxa from either North or Central America; therefore, we did not test any hypotheses regarding the CAS, but only those associated with GAARlandia. In the BioGeoBEARS analyses of the tree where *Euptychia* is not sister to *Calisto*, we tested all 252 models.

Results (S4, S5, S6 Figs, Figs 6, 7)

*Calisto* butterflies are only found in the Greater Antilles, so geographic areas for the BioGeoBEARS analyses were set up differently than for the other analyses (S6 Fig). For the *Calisto* dataset in which *Euptychia* is the sister taxon to *Calisto*, we tested 18 models that either included or did not include GAARlandia, were distance dependent or independent, or a “vicariance” only model, in addition to the 6 models in BioGeoBEARS. The best relative model according to the AICc values is DEC + J A1b and had the following constraints: dispersal from one land area to another is not affected by distance, with founder event dispersal playing a role, and without GAARlandia as a means of dispersal from South America to the Greater Antilles (Table 2). Ancestral range estimation (S4 Fig, Fig 6) indicates one or two dispersal events to the Caribbean from South America to Hispaniola and from there to other islands, including dispersal to Jamaica around 15 mya. (However, according to Matzke [67], the plot of most-probable states isn’t the same as event counts. Parsimoniously, there was a single dispersal event to Hispaniola, followed by diversification and additional dispersal to other islands. Simple counts give some idea but do not consider events occurring along a branch; for better estimates, biogeographic stochastic mapping would be required.) Additionally, there were 2 dispersal events to the Bahamas and only 1 to Jamaica. For the *Calisto* dataset in which *Euptychia* is not the sister taxon to *Calisto*, multiple DEC + J models were equiprobable (Table 2) – these were: A2a, B2a, D2a, E2a, and G2a. All support a DEC model, with founder event dispersal, and with the following constraints: dispersal from one landmass to another is affected by distance, and GAARlandia played a role in dispersal from South America to the Greater Antilles. The analyses cannot differentiate between the best model regarding dispersal time from North America to South America, which might be expected given that there are no Central American taxa that might help to differentiate between such models. Ancestral range estimation is only shown for A2a (S5 Fig, Fig 7) because all were similar, with the only differences occurring within the outgroup taxa, either indicating an ancestral range of South America or North America for the entire group. Ancestral range estimation indicates 2 dispersal events to the Caribbean, one from South America to Puerto Rico and the other from South America to Hispaniola, with subsequent diversification, dispersal to Cuba, the Bahamas, and Jamaica.

**S4 Fig. Phylogeny and ancestral range estimation for *Calisto* butterflies (*Euptychia* as the outgroup).**

BioGeoBEARS phylogram corresponding to the DEC + J A1b model (Table 1). Pie charts represent the probabilities of each possible geographical area before and after each split. Colors correspond to S6 Fig. SA = South America; CU = Cuba; JA = Jamaica; HI = Hispaniola; PR = Puerto Rico; BA = Bahamas. Photo by S. Crews.


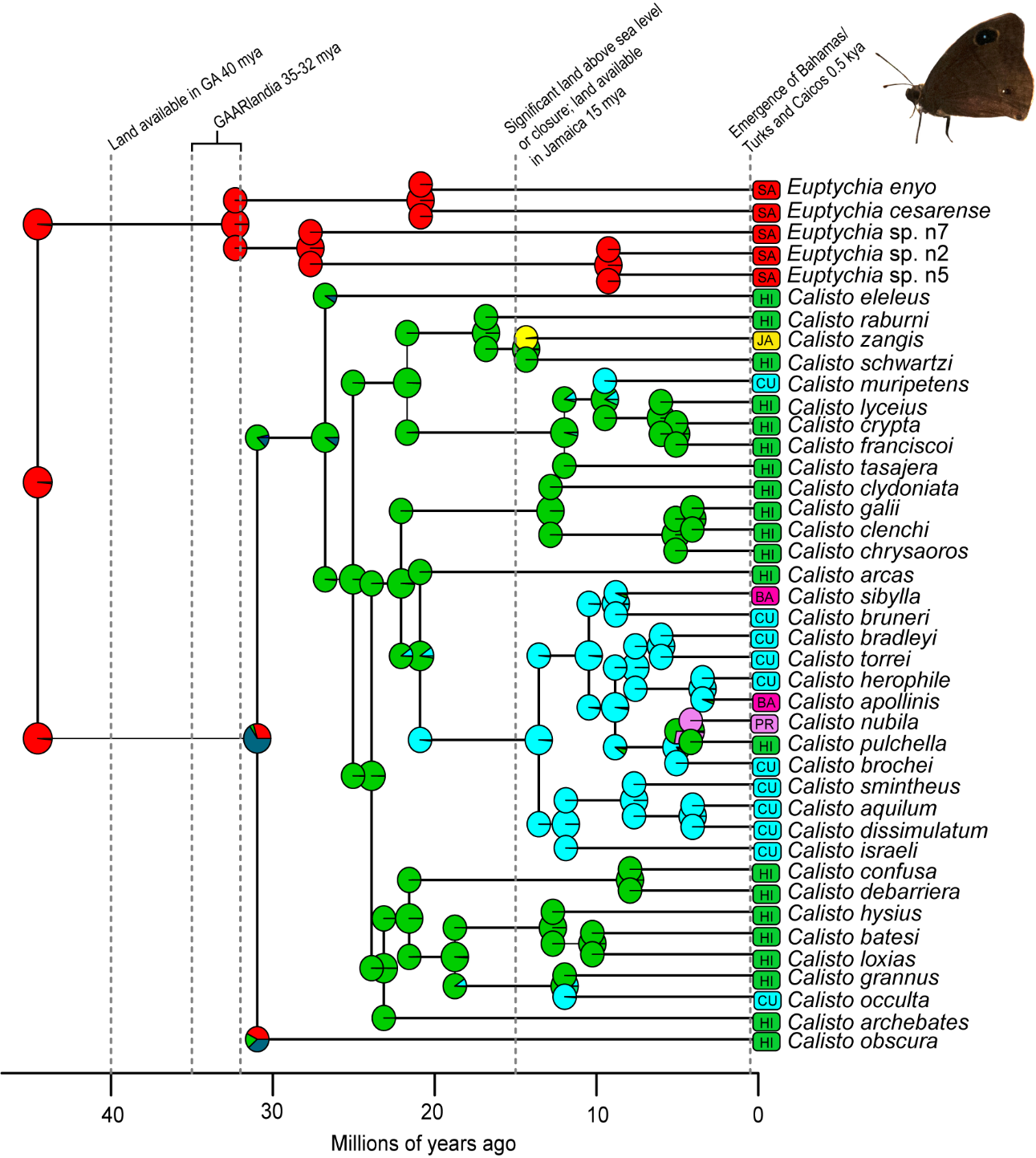


**S5 Fig. Ancestral range estimation for *Calisto* butterflies (*Euptychia* is not the sister taxon).**

BioGeoBEARS phylogram corresponding to the DEC + J A2a model (B2a, D2a, E2a, and G2a being equiprobable as the best models based on the AICc weights) (Table 1). Pie charts represent the probabilities of each possible geographical area before and after each split. Colors correspond to S6 Fig. SA = South America; NA = North America; CU = Cuba; JA = Jamaica; HI = Hispaniola; PR = Puerto Rico; BA = Bahamas. Photo by S. Crews.


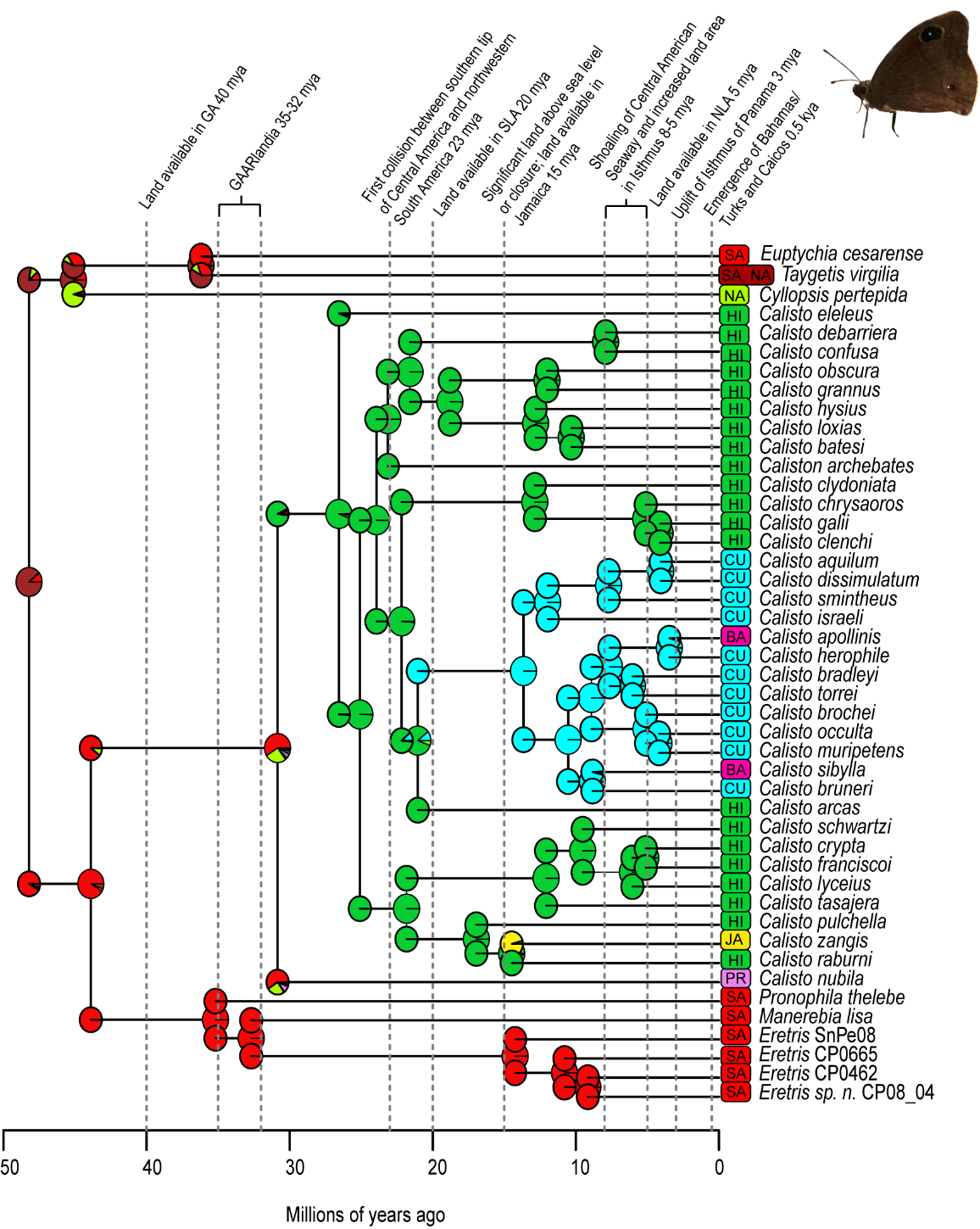


**S6 Fig. Map of the geographical areas used in the *Calisto* BioGeoBEARS analyses.**

This butterfly genus is only found in the Greater Antilles, so these analyses were set up somewhat differently than the others. Colors correspond to those used in the *Calisto* figures of ancestral range reconstructions from BioGeoBEARS analyses. For information on geographic areas and geologic dates, see Table S1, Table 1, and text. Base map created in ArcGIS v9.2 and areas shaded in Adobe Illustrator Creative Cloud. Red = South America (SA); Yellow-Green = North America (NA); Bright Pink = Bahamas and Turks and Caicos Islands (BA, TCI); Blue = Cuba (CU); Yellow = Jamaica (JA); Green = Hispaniola (HI); Pink = Puerto Rico (PR).


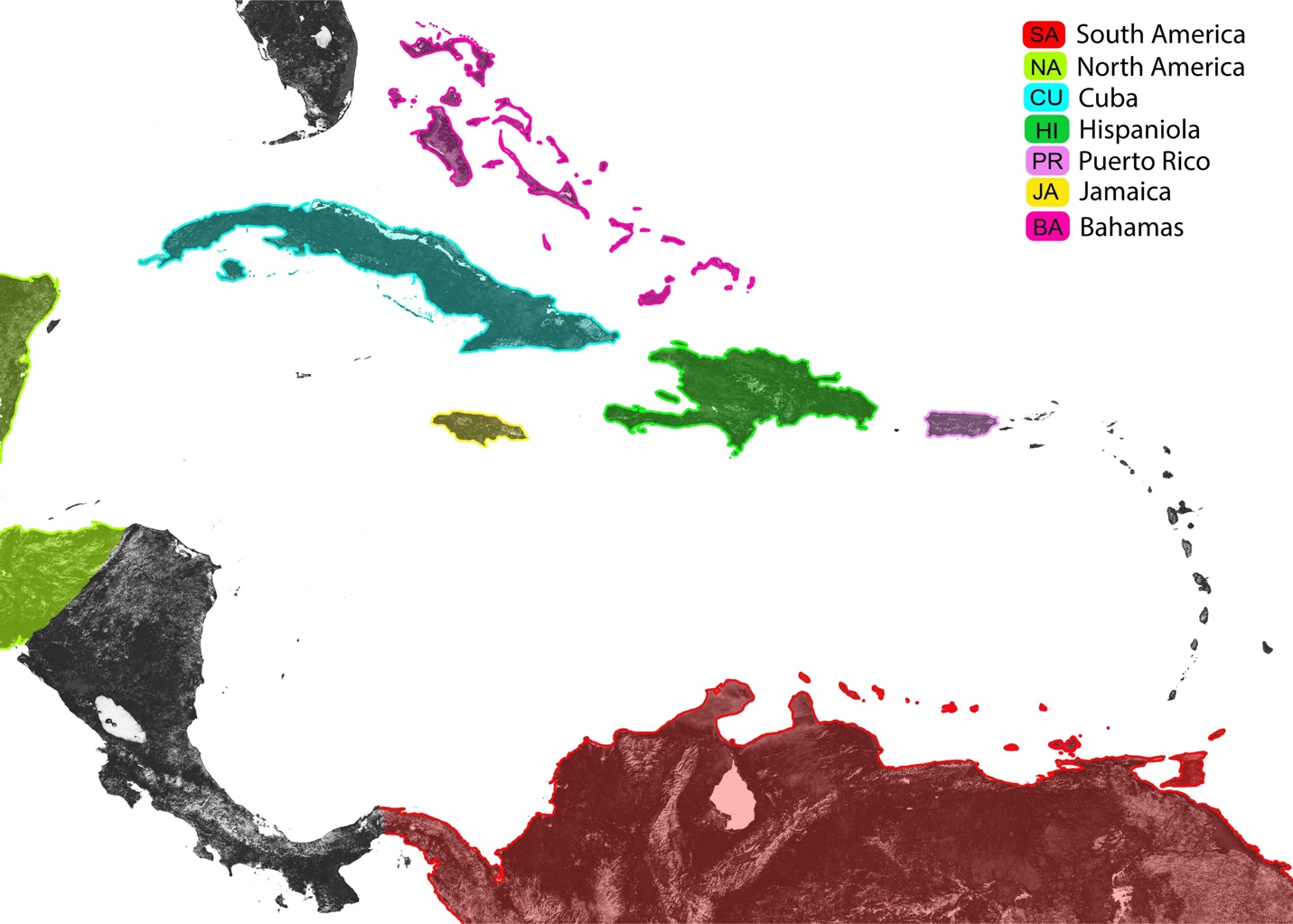


**S9 Table.** ***Calisto* butterfly sample information.** *Calisto* species used in this paper and their distributions. GenBank accession numbers can be found in Matos-Maraví et al. [23]. All *Calisto* species were used in both BioGeoBEARS analyses, all *Euptychia* species were used in one analysis, and *E. cesarense* and the other outgroups were used in the second analysis (see text for details) (SA=South America; CU=Cuba; HI=Hispaniola; JA=Jamaica; BA=Bahamas; PR=Puerto Rico).

| *Calisto apollinis* RN02_25 ^(BA)^ |
| --- |
| *Calisto aquilum* PM20_03 ^(CU)^ |
| *Calisto arcas* NW149_16 ^(HI)^ |
| *Calisto archebates* RN02_14 ^(HI)^ |
| *Calisto batesi* RN03_02 ^(HI)^ |
| *Calisto bradleyi* PM07_26 ^(CU)^ |
| *Calisto brochei* PM07_03 ^(CU)^ |
| *Calisto bruneri* PM07_21 ^(CU)^ |
| *Calisto chrysaoros* RN04_01 ^(HI)^ |
| *Calisto clenchi* RN06_01 ^(HI)^ |
| *Calisto clydoniata* RN02_16 ^(HI)^ |
| *Calisto confusa* DR016 ^(HI)^ |
| *Calisto crypta* CP20_02 ^(HI)^ |
| *Calisto debarriera* DR019 ^(HI)^ |
| *Calisto dissimulatum* PM07_20 ^(CU)^ |
| *Calisto eleleus* RN03_01 ^(HI)^ |
| *Calisto franciscoi* CP19_14 ^(HI)^ |
| *Calisto galli* DR017 ^(HI)^ |
| *Calisto grannus* RN06_02 ^(HI)^ |
| *Calisto herophile* PM07_22 ^(CU)^ |
| *Calisto hysius* RN04_02 ^(HI)^ |
| *Calisto israeli* PM07_02 ^(CU)^ |
| *Calisto loxias* RN02_18 ^(HI)^ |
| *Calisto lyceius* DR075 ^(HI)^ |
| *Calisto muripetens* RN01_05 ^(CU)^ |
| *Calisto nubila* CP19_10 ^(PR)^ |
| *Calisto obscura* DR080 ^(HI)^ |
| *Calisto occulta* PM07_18 ^(CU)^ |
| *Calisto pulchella* DR003 ^(HI)^ |
| *Calisto raburni* CAL_Sat102 ^(HI)^ |
| *Calisto schwartzi* DR063 ^(HI)^ |
| *Calisto sibylla* RN02_22 ^(BA)^ |
| *Calisto smintheus* PM07_05 ^(CU)^ |
| *Calisto tasajera* CP20_08 ^(HI)^ |
| *Calisto torrei* PM07_11 ^(CU)^ |
| *Calisto zangis* RN02_12 ^(JA)^ |
| *Euptychia cesarense* CP04_55 ^(SA)^ |
| *Euptychia enyo* CP06_73 ^(SA)^ |
| *Euptychia* spn2 CP01_33 ^(SA)^ |
| *Euptychia* spn5 CP01_53 ^(SA)^ |
| *Euptychia* spn7 CP02_58 ^(SA)^ |
| *Manerebia lisa* CP04_23 ^(SA)^ |
| *Eretris* SnPe08 ^(SA)^ |
| *Pronophila thelebe* CP03_70 ^(SA)^ |
| *Taygetis virgilia* NW108_3 ^(SA, NA)^ |
| *Eretris* sp. n. 8 CP08_04 ^(SA)^ |
| *Eretris* CP04_62 ^(SA)^ |
| *Eretris* CP06_65 ^(SA)^ |

**Details of *Papilio* Butterflies Analyses**

Methods

After downloading the sequences used in Lewis et al. [53] from GenBank and attempting to set up the same BEAST 2 analysis as these authors, we were unable to get the dataset to run (S10 Table). The authors graciously gave us their BEAST .xml input files upon request which we did not alter before running, but we were unable to obtain the same results regarding divergence dates (the dates from the authors’ datasets were much younger than those in Fig. 5 of Lewis et al. [53]), and the analyses had very low ESS values; the authors did not respond to requests for their tree files. We certainly understand the difficulties in keeping track of all the various inputs and outputs when running many analyses, and assume they sent us the wrong ones. Thus, we started from scratch.

Using Appendix S3 from Lewis et a. [53], we downloaded all the sequences that included both CoxI and II and EF1α, unless only 1 gene was available for a particular taxon, and then it was also included to maximize both molecular and taxonomic sampling, for a total of 73 taxa. We ran PartitionFinder2 [104] and then ran MrBayes.

We added the morphological data from the authors’ BEAST input .xml file to our own dataset before running BEAST 2. We first ran the analyses using BMT, with a relaxed log normal clock model, and a Yule process of speciation, and then altered parameters in subsequent analyses until we achieved high ESS values for each partition. Our final analysis used an HKY model for partitions 2 and 5, the JC model for the morphology partition, and BMT for partitions 1, 3, and 4. A relaxed log normal clock model was used for all partitions. Because the authors [53] found no major differences between the Yule and Birth Death models of speciation, we used the Yule model. Our calibration constraints followed those of Lewis et al. [53] and were 1) The Root Node prior, using a uniform distribution with a lower bound of 40.61 and an upper bound of 57.39. 2) The Teinopalpini prior, with a uniform distribution, and a lower bound of 27.39 and an upper bound of 44.08. The taxa included were *Meandrusa payeni*, *M. sciron*, *Teinopalpus aureus*, and *T*. *imperialis*. 3) the *Papilio* prior, with a uniform distribution, a lower bound of 25.74 and an upper bound of 38.28. This included all the *Papilio* taxa. 4) Finally, because *P. melonius* kept appearing outside of the Caribbean clade, likely due to having so little data (morphology only), we used the authors’ placement as they are experts on the group and constrained the relationship of *Papilio cresphontes* and *P. melonius*.

Because the root node of *Papilio* was dated to be younger than the proposed timing of GAARlandia, only hypotheses concerning the IoP were tested. For *Papilio cresphontes* we found conflicting information about the distribution as some sources included it in Jamaica whereas others did not. According to Butterflies and Moths of North America [121], *P. cresphontes* is found from the United States to Panama, and Cuba, and sporadic elsewhere; thus, we did not include Jamaica as being part of the standard distribution for BioGeoBEARS analyses. Distributions used can be found in S10 Table.

Results (S7 Fig, Fig 8)

Our recovered MrBayes topology was not identical to that of Lewis et al. [53], which is to be expected given that different taxa and genes were used in each analysis; however, there were no major differences, and no differences were supported. BAYAREALIKE + J C1a and BAYAREALIKE + J D1a were both equally the best models based on the AICc weight relative probabilities (Table 2), or a model with founder event dispersal with the following constraints: dispersal between landmasses is affected by distance, and dispersal across the IoP/CAS could have begun at 15 or 8 mya. The analysis indicates a South American origin for the Caribbean fauna with at least 4 dispersal events to the Caribbean islands, including 4 separate dispersals to Jamaica. The ancestral range estimation also indicates dispersal events from the Greater Antilles to the mainland.

**S7 Fig. Ancestral range estimation for *Papilio* butterflies.**

BioGeoBEARS phylogram corresponding to the BAYAREALIKE + J C1a model (D1a is equally likely) (Table 1). Pie charts represent the probabilities of each possible geographical area before and after each split. Colors correspond to Figure 1. SA = South America; CA = Central America; NA = North America; GA = Greater Antilles; JA = Jamaica; SLA = Southern Lesser Antilles; NLA = Northern Lesser Antilles; FL = Florida; BA = Bahamas. *Papilio* photo by Eduardo Lopez.


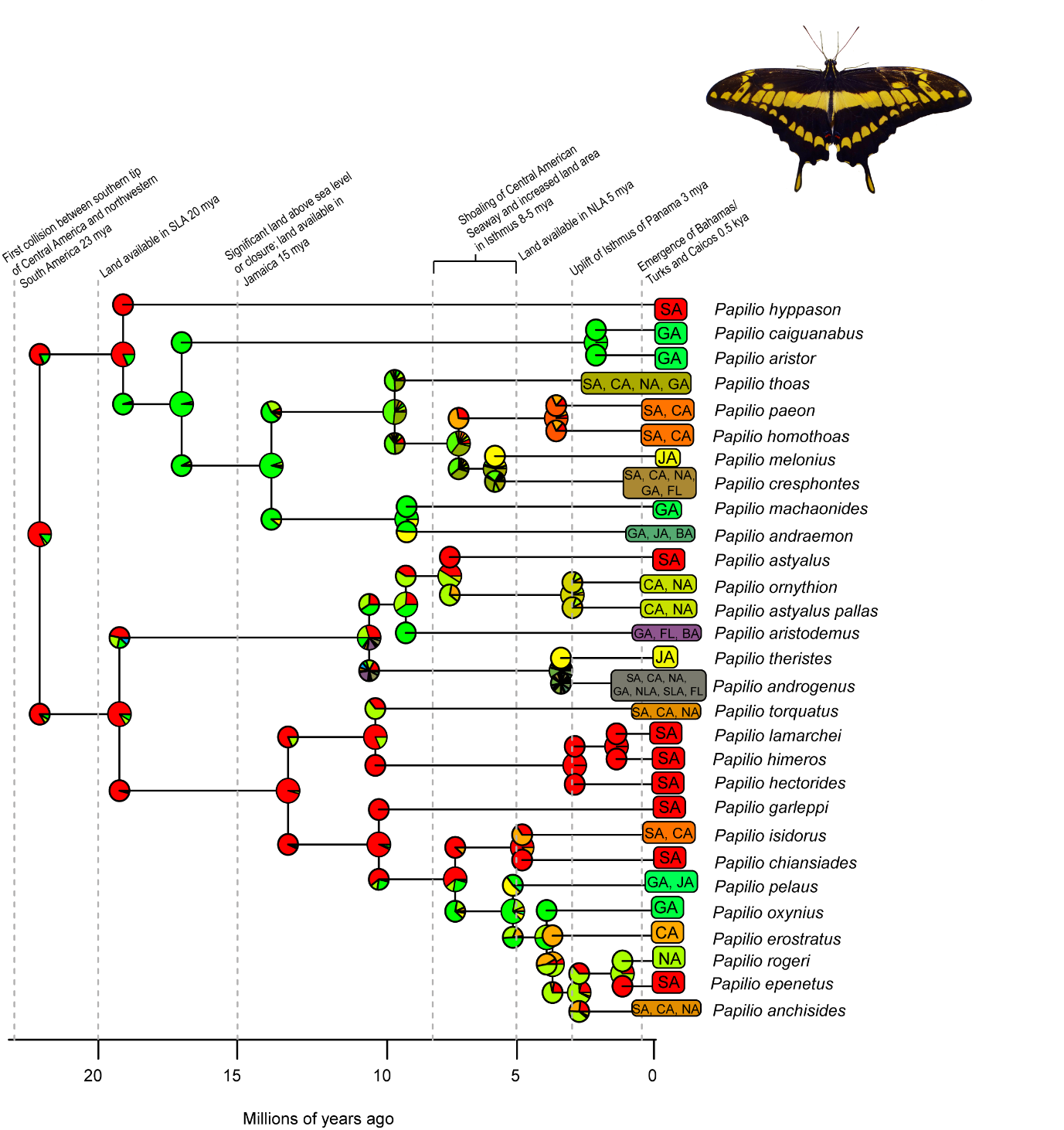


**S10 Table**. ***Papilio* butterfly sample information.** *Papilio* species used in the BioGeoBEARS analyses and their distributions. GenBank accession numbers can be found in Lewis et al [53]. (SA=South America; CA=Central America; NA=North America; GA=Greater Antilles; JA=Jamaica; NLA=Northern Lesser Antilles; SLA=Southern Lesser Antilles; FL=Florida; BA=Bahamas and/or Turks and Caicos Islands).

| *Papilio hyppason* ^(SA)^ |
| --- |
| *Papilio aristor* H032 ^(GA)^ |
| *Papilio caigunabus* ^(GA)^ |
| *Papilio machaonides* SH02 ^(GA)^ |
| *Papilio andraemon* H005 ^(GA, JA, BA)^ |
| *Papilio thoas* 9900302 ^(SA, CA, NA, GA)^ |
| *Papilio melonius* ^(JA)^ |
| *Papilio cresphontes* ^(SA, CA, NA, GA, FL)^ |
| *Papilio paeon* ^(SA, CA)^ |
| *Papilio homothoas* ^(SA, CA)^ |
| *Papilio androgeus* SP779 ^(SA, CA, NA, GA, NLA, SLA, FL)^ |
| *Papilio thersites* H003 ^(JA)^ |
| *Papilio aristodemus* SH01 ^(GA, FL, BA)^ |
| *Papilio astyalus pallas* 615 ^(CA, NA)^ |
| *Papilio ornythion* SP290 ^(CA, NA)^ |
| *Papilio astyalus* SP762 ^(SA)^ |
| *Papilio torquatus* 9900043 ^(SA, CA, NA)^ |
| *Papilio hectorides* 9900045 ^(SA)^ |
| *Papilio lemarchei* SP990 ^(SA)^ |
| *Papilio himeros* ^(SA)^ |
| *Papilio garleppi* SP984 ^(SA)^ |
| *Papilio isidorus* P54_1 ^(SA, CA)^ |
| *Papilio chiansiades* H027 ^(SA)^ |
| *Papilio pelaus* ^(GA, JA)^ |
| *Papilio oxynius* ^(GA)^ |
| *Papilio erostratus* 9900973 ^(CA)^ |
| *Papilio anchisiades* SP267 ^(SA, CA, NA)^ |
| *Papilio rogeri* ^(NA)^ |
| *Papilio epenetus* P4_2 ^(SA)^ |

**Details of *Drosophila* Flies Analyses**

Methods

We began by downloading sequences from GenBank from 187 taxa covering the genes CoxI, CoxII, 16S, ND1, amd, ddc, hb, kl5, sod, and the y exon (S11 Table). This dataset contained a lot of missing data (83%), and we know that there is little concordance between mitochondrial and nuclear genes in this group [122]. For our approach, we decided to minimize the missing data while attempting to maintain the species of interest and their outgroups. This involved constructing a few chimeric sequences. We used 16S-ND1, CoxI, CoxII and the intervening tRNA, adh, and amd. Genes for each taxon were assembled in SequenceMatrix [116] and aligned by eye in Mesquite [110] using codons and ensuring there were no stop codons. We ran MrBayes analyses, including an analysis of mtDNA, an analysis of the nuclear genes, and a concatenated dataset, to determine how our results compared to published data.

Due to the uncertainty of the placement of *D. neomorpha* and *D. polymorpha* in relation to the “dunni” group and the “cardini” group, we used three trees in BEAST 2 with the following relationships: 1) ((neomorpha+polymorpha)+cardini group)dunni group; 2) ((cardini group +dunni group)neomorpha+polymorpha; 3) ((neomorpha+polymorpha)+dunni group)cardini group. We calibrated the trees using methods similar to those of Obbard et al. [123], in which they found that using a mutation rate for *Drosophila* provided better estimates for divergence dating than using fossils or biogeographic events; however, we did not use only 4-fold degenerate codons in our analysis as they did. We tried various analyses with and without altering the Gamma distribution of the clock standard deviation; we found that changing this parameter made little difference in divergence time estimation. We used *D. immigrans*, *D. simulans*, *D. melanogaster*, and *D. yakuba* as outgroups and ensured that the timings from the calibration scheme in our analyses were similar to those of Obbard et al. [123] for the same taxa. MrBayes analyses indicated a polytomy concerning the relationships of *D. neomorpha* + *D. polymorpha* with the “dunni” group and the “cardini” group, and BEAST 2 results indicated a relationship of ((“dunni” group + (neomorpha+polymorpha))”cardini” group, so we used the 3 trees depicting the various relationships of these 3 groups for subsequent BioGeoBEARS analyses. Finally, because we obtained a result of *D. acutilabella* branching before *D. cardini* and not being sister to *D. belladunni* as has been indicated by Brisson et al. [124] and Cenzi et al [122], we took the tree with the highest Bayes Factor from the previous 3 analyses and constrained the *D. belladunni* + *D. acutilabella* relationship on this tree in a final BEAST 2 run. This tree and the 3 with the relationship unconstrained were used for BioGeoBEARS analyses. For the BEAST 2 analyses, the ESS values for some BMT parameters and the rate parameter for partition 7 were slightly less than 200 but changing input parameters did not make a difference. Because the values were close to 200 or seemed unlikely to change the results, we went forward using these resultant trees for the BioGeoBEARS analyses. Because the group is young in the Caribbean according to our results (<12 my), the flies clearly entered after GAARlandia, and we could only test models A1a-A1c, D1a-D1c, and G1a-G1c (Table 1). Fly distributions were taken from: [125] <http://evolution.ibmc.up.pt/node/35> and [126] <https://stockcenter.ucsd.edu/info/geography.php> (the latter compiled from Ashburner et al. [127], as well as Cenzi et al. [122] and Brisson et al. [124]).

Results (S8-S9 Figs, Figs 9, 10)

As in Cenzi et al [122], there is discordance among the trees resulting from the different datasets, largely to do with the placement of *Drosophila neocardini* as well as the placement of *D. neomorpha* and *D. polymorpha*. Additionally, an early branching of *D. cardini* was found in our analyses, congruent with analyses of Cenzi et al [122] and hybridization tests of Futch [145] and Heed [146]. In our tree from the MrBayes analysis, *D. acutilabella* branched earlier than *D. cardini*, and this is well-supported (PP=1.0). Bayes Factors indicated that the best tree was the unconstrained tree (the relationship of *D. acutilabella* and *D. belladunni* was not constrained) from the BEAST 2 analysis with the following relationships (((*neopolymorpha*+*polymorpha*)+dunni group)“cardini” group). Due to uncertainty in the relationships, we conducted four suites of analyses in BioGeoBEARS using four different input trees. For three analyses, the model with the highest relative probability based on the AICc weights is BAYAREALIKE + J D1a (Table 2), and for the ((“cardini” group+dunni group) *neomorpha*+*polymorpha*) tree, a slightly different model was recovered: BAYAREALIKE + J D1b (Table 2). We have chosen to illustrate the ancestral range estimation for the (((*neopolymorpha*+*polymorpha*)+dunni group)”cardini” group) tree with the relationship of *D*. *belladunni* and *D*. *acutilabella* constrained given results of previous authors who are more familiar with *Drosophila* (S8 Fig). The favored model is one in which distance may or may not affect dispersal and where dispersal across the IoP/CAS began 8 mya. Ancestral range estimation indicates at least 5 dispersal events to the Caribbean region, and there is no evidence of dispersal to the mainland from the islands.

*Drosophila* fruit flies are a relatively young group of good dispersers [63], and our results indicate multiple dispersal events. Brisson et al. [124] concluded a single dispersal event for the *D. dunni* subgroup, but it was unclear that the dispersal was directly from South America. Their results also indicated that dispersal throughout the islands didn’t really follow a pattern of island hopping for this group, indicating that Puerto Rico was the starting point. However, our analyses indicate that dispersal began via the Southern Lesser Antilles and proceeded to other islands, but that dispersal did occur in a chaotic manner.

**S8 Fig. Ancestral range estimation for *Drosophila* flies.**

BioGeoBEARS phylogram corresponding to the BAYAREALIKE + J D1a model (Table 1) for the tree with the relationship of *D. belladunni* and *D. acutilabella* constrained. Pie charts represent the probabilities of each possible geographical area before and after each split. Colors correspond to Figure 1. SA = South America; CA = Central America; NA = North America; GA = Greater Antilles; JA = Jamaica; SLA = Southern Lesser Antilles; NLA = Northern Lesser Antilles; FL = Florida; BAR = Barbados. *Drosophila* photo by Mark Yokoyama.


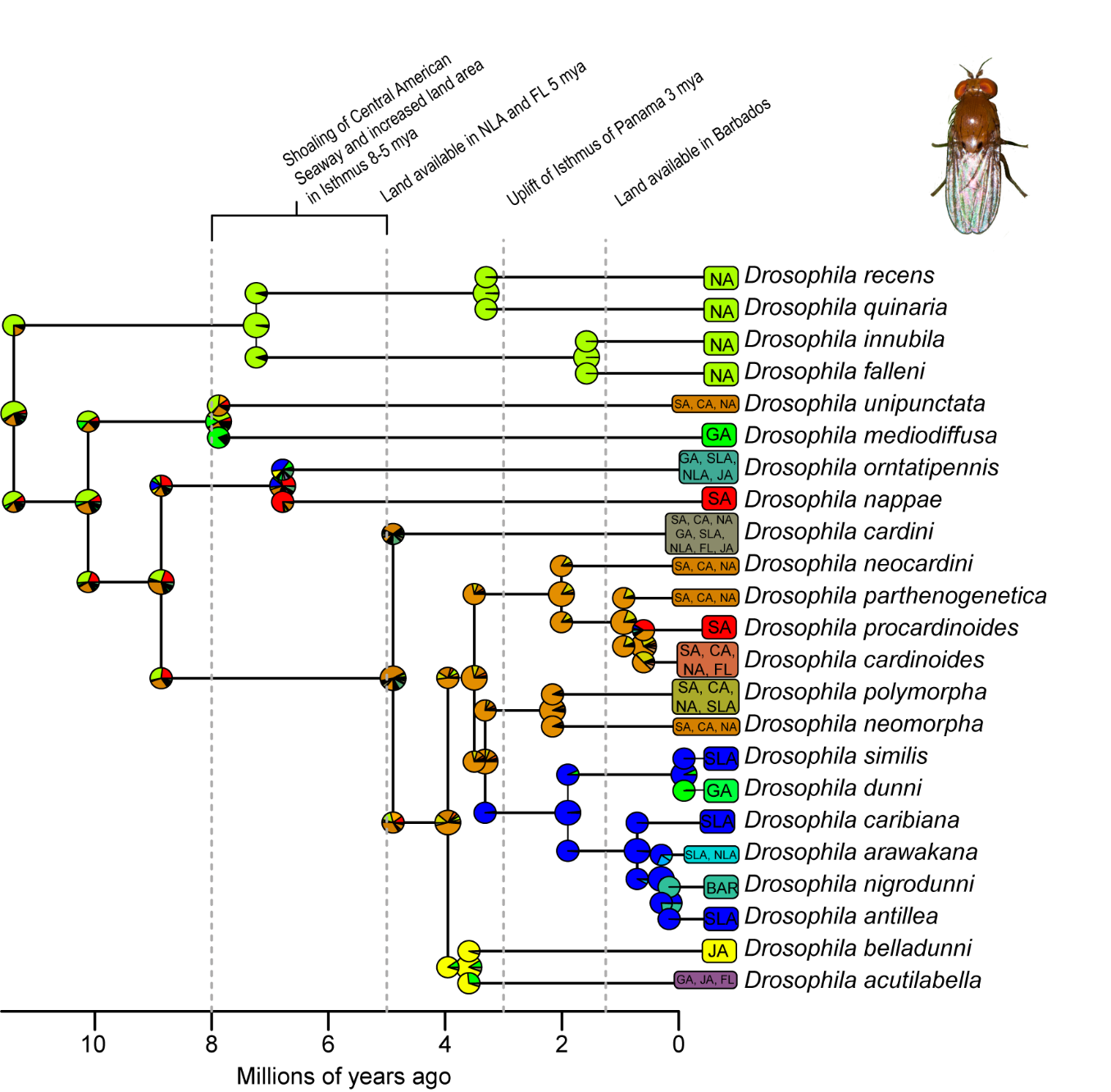


**S11 Table.** **Sequences for *Drosophila* analyses.** These fly sequences and outgroups were downloaded from GenBank and used in the BEAST 2 analyses. The species with asterisks were not used in BioGeoBEARS analyses. Distributions considered for each species used in the BioGeoBEARS analysis are provided. (SA=South America; CA=Central America; NA=North America; GA=Greater Antilles; Ja=Jamaica; SLA=Southern Lesser Antilles; NLA=Northern Lesser Antilles; BAR=Barbados; FL=Florida) Note: some outgroup sequences are chimeric (the 16S and COII sequences are not from the same individuals) to be able to include more taxa. - indicates that no data were available for that gene. For *D. procardinoides*, Cenzi et al [43] list it as being collected in the Everglades, FL; however to our knowledge this species is only found in South America and “Everglades, Florida” is perhaps a mistake made when uploading the sequence to GenBank; the same for *D. ornatipennis* from [49] as we are unaware that this species is found in Brazil and a collection locality is not provided in the paper.

| Species | COI | COII | 16S-ND1 | adh | amd |
| --- | --- | --- | --- | --- | --- |
| *Drosophila acutilabella* ^(GA, JA, FL)^ | Everglades;HM006873  [122] | 15181-2171.10; AB932761; [184] | Florida; 15181-2171.9; AF246509; [185] | 15181-2171.10; AB932645; [184] | Everglades; HM006861; [122] |
| *Drosophila antillea* ^(SLA)^ | - | BDI1; AY173183; [185] | StL; AF246502; [185] | AY695383; [186] | - |
| *Drosophila arawakana* ^(SLA, NLA)^ | StKN; HM006881; [122] | 15182-2261.03; AB932764; [184] | MtJoy line 1; AF246516; [185] | 15182-2261.03; AB932647; [184] | StKN; EU444555; [187] |
| *Drosophila belladunni* ^(JA)^ | - | Jamaica; AF246479; [185] | Jamaica; AF246506; [185] | AY695385; [186] | - |
| *Drosophila cardini* ^(SA, CA, NA, GA, SLA, NLA, JA, FL)^ | 103963; EU493576; [188] | E-17701; AB932767; [184] | 103963; 3u494310; [188] | strain: E-17701; AB932650; [184] | Itaqui; EU444558; [187] |
| *Drosophila cardinioides* ^(SA, CA, NA, FL)^ | L004-16; EF569992; [189] | 15181-2291.00; AB932768; [184] | 15181-2192.8; Mex ; AF246518; [185] | strain:15181-2291.00; AB932651; [184] | Porto Alegre; EU444559; [187] |
| *Drosophila caribiana* ^(SLA)^ | JQ679114; [190] | Martinique; AF246466; [185] | Martinique; AF246493; [185] | AY695387; [186] | - |
| *Drosophila dunni* ^(GA)^ | 103969l; EU493577; [188] | 15182-2291.00; AB932771; [184] | 103969; eu494311; [188] | strain: 15182-2291.00; AB932654; [184] | USVIStT; HM006865; [183] |
| *Drosophila falleni* ^(NA)^ | Roc_M1; AY541122; [191] | AB932772; [184] | AF479802; [192] | AB932655; [184] | - |
| *Drosophila immigrans** | isolate i01; AB824775; [193] | AB932779; [184] | 103956; EU494320; [188] | M97638; [194] | Joinville; EU444567; [197] |
| *Drosophila innubila* ^(NA)^ | CH_F11; AY541182; [191] | AB932780; [184] | - | AB932660; [184] | - |
| *Drosophila mediodiffusa* ^(GA)^ | voucher 109396; EU493616; [188] | 109396; EU493745; [188] | AF246464; [185] | - | PR; EU444572; [187] |
| *Drosophila melanogaster** | isolate ALST; KP843842; [195] | GQ376046; [196] | ISO1; KP161877; [197] | KJ767237; [198] | [199] |
| *Drosophila nappae* ^(SA)^ | L002-17; EF570005; [189] | isolate H05; AY162983; [200] | - | - | Porto Alegre; EU444579; [187] |
| *Drosophila neocardini* ^(SA, CA, NA)^ | PortoAlegre; HM006876; [183] | PortoAlegre; HM006886; [183] | AF246505; Brazil; [185] | - | Porto Alegre; EU444581; [187] |
| *Drosophila neomorpha* ^(SA, CA, NA)^ | Joinville; HM006877; [183] | Joinvile; HM006891; [183] | Panama; AF246517; [185] | - | Joinville; HM006862; [183] |
| *Drosophila nigrodunni* ^(BAR)^ | Barbados; HM006883; [183] | 15182-2311.00; AB932786; [184] | Barbados, 15182-2311.1; AF246494; [185] | strain: 15182-2311.00; AB932663; [184] | Barbados; EU444582; [187] |
| *Drosophila ornatipennis* ^(GA, SLA, NLA, JA)^ | Serra do Japi, Jundiai; EF570010; [187] | 103965; EU494307; [188] | 103965; EU493704; [188] | AY081443; [192] | Guad; EU444585; [187] |
| *Drosophila parthenogenetica* ^(SA, CA, NA)^ | Florianopolis; HM006878; [183] | 15181-2221.00; AB932790; [184] | 15181-2221.1 Mexico; AF246510; [185] | strain: 15181-2221.00; AB932666; [184] | Florianopolis; HM006863; [183] |
| *Drosophila polymorpha* ^(SA, CA, NA, SLA)^ | PortoAlegre; HM006879; [183] | 15181-2231.00; AB932792; [184] | Brazil 15182-2231.1; AF2246507; [185] | strain: 15181-2231.00; AB932668; [184] | Florianopolis; EU444591; [187] |
| *Drosophila procardinoides* ^(SA)^ | Everglades; HM006880; [183] | Bolivia; HM006888; [183] | Brazil; AF246508; [185] | - | Bolivia; HM006864; [183] |
| *Drosophila quinaria* ^(NA)^ | 107542; EU493605; [188] | AB932794; [184] | 107542; eu494338; [188] | AB932670; [184] | - |
| *Drosophila recens* ^(NA)^ | R-SM/TN_Y9; DQ851700; [201] | AB932795; [184] | - | AB932671; [184] | - |
| *Drosophila similis* ^(SLA)^ | 15182-2321.00; AB932753; [184] | 15182-2321.00; AB932798; [184] | SVG; AF246495; [185] | strain: 15182-2321.00; AB932673; [184] | - |
| *Drosophila simulans** | Hawaii; M57909; [202] | strain sm21; KC244283; [203] | KY0418; JQ691660; [195] | 14021-0251.167; KJ767241; [198] | from Brazil; AY699262; [187] |
| *Drosophila unipunctata* ^(SA, CA, NA)^ | L008-6; EF570020; [189] | L008-6; EF570047; [189] | KT272846; [204] | - | Colombia; EU444595; [187] |
| *Drosophila yakuba** | X03240; [205] | strain yak_12Y full mt; KF824873; [206] | yak_12Y; KF824873; [206] | 14021-0261.0; KJ767242; [198] | - |

**Details of Centruroidinae Scorpions Analyses**

Methods

Scorpion distributions used in the BioGeoBEARS analyses can be found in S12 Table. Because these data have been thoroughly analyzed recently, and we have confidence in the relationships [55], we began by conducting a BEAST 2 analysis. Calibration constraints were as follows: 1) New World buthids, including everything but *I. maculatus*, with a normal distribution, a geologic constraint of 30 mya with a large standard deviation; 2) a Greater Antilles constraint using a Dominican amber fossil, containing the following taxa: *Centruroides arctimanus*, *C. bani*, *C. barbudensis*, *C. farri*, *C. griseus borinquensis*, *C. griseus, C. griseus*, *C. guanensis*, *C. hentzi*, *C. insulanus*, *C. luceorum*, *C. mariaorum*, *C. nitidus taino*, *C. platnicki*, *C. pococki*, and *C. sasae* with a log normal distribution, a mean of 15 and sigma of 1.25; 3) a Chiapas amber fossil constraint containing *C. rileyi*, *C. schmidti*, and *C. sissomi*, with a log normal distribution, a mean of 15, and sigma of 1.25. The final analysis used a Yule model of speciation, a relaxed log normal clock model, HKY for 12S, 16S and 28S and JC model of evolution for 18S and COI. Outgroup taxa were pruned from the tree in Mesquite [110], and all 252 models were tested in BioGeoBEARS.

Results (S10 Fig, Fig 11)

Similar to the Lagrange analysis conducted previously which favored a distance-dependent dispersal model, this analysis found two similar models were equiprobable according to the relative model probabilities based on the AICc weights: BAYAREALIKE + J B1a and BAYAREALIKE + J B2a (Table 2). The favored model indicates founder event dispersal, where distance affects dispersal from one landmass to another, dispersal across the IoP/CAS occurred as early as 23 my, and GAARlandia either did or did not play a role in dispersal from South America to the Greater Antilles. Ancestral range estimation shows 2 primary dispersal events to the Greater Antilles, one from North America giving rise to a Caribbean endemic clade of *Centruroides*, and one from South America giving rise to the Greater Antilles endemic genus *Heteroctenus*. A third dispersal event occurred from South America to the Lesser Antilles.

**S9 Fig. Ancestral range estimation for Centruroidinae scorpions.**

BioGeoBEARS phylogram corresponding to the BAYAREALIKE + J B1a and B2a models (Table 1). Pie charts represent the probabilities of each possible geographical area before and after each split. Colors correspond to Figure 1. SA = South America; CA = Central America; NA = North America; GA = Greater Antilles; JA = Jamaica; SLA = Southern Lesser Antilles; NLA = Northern Lesser Antilles; FL = Florida; BA = Bahamas. Photo by S. Crews.


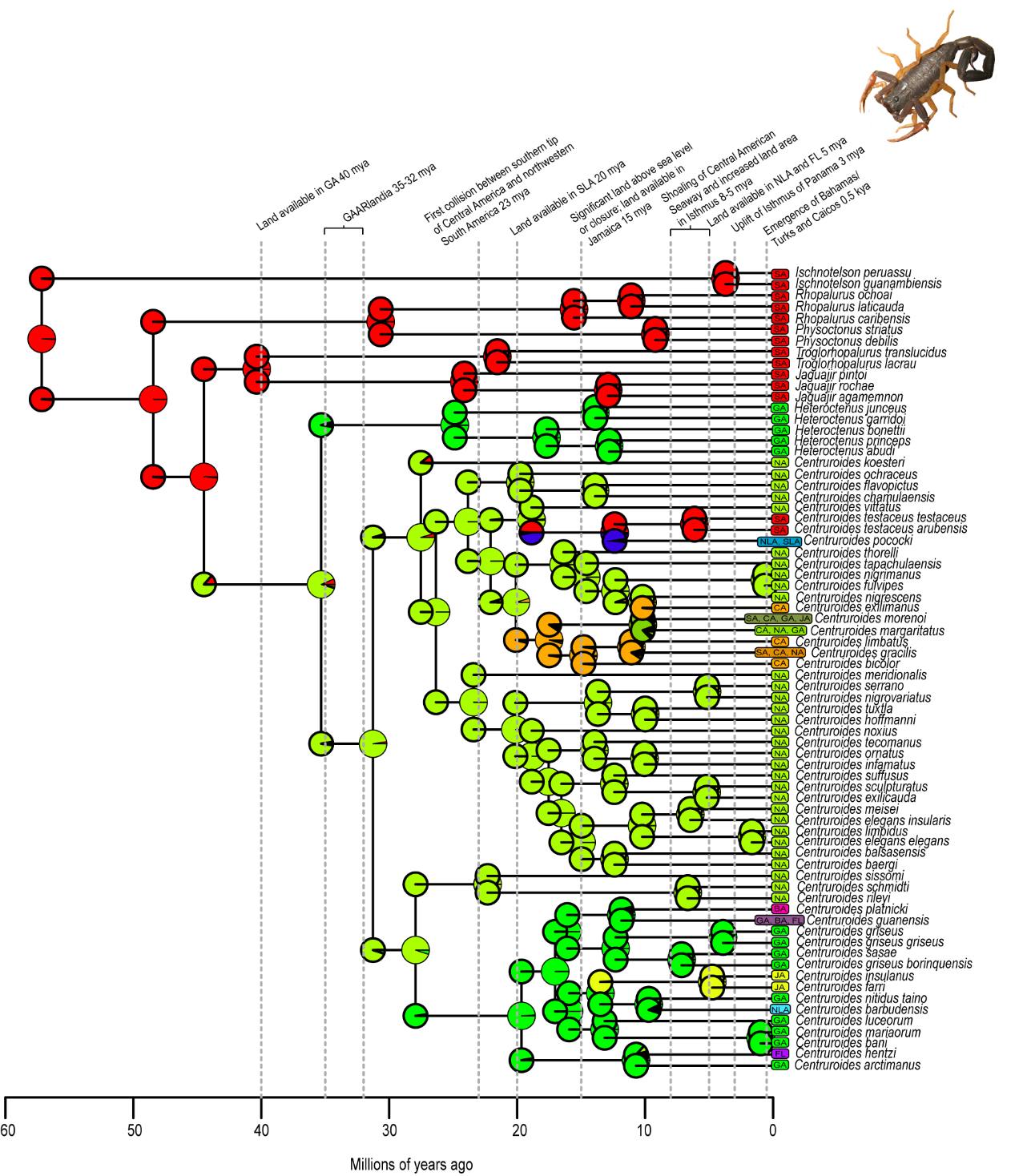


**S12 Table.** **Sequences for Centruroidinae scorpion analyses**. Centruroidinae scorpion species used in the BioGeoBEARS analyses and their distributions. GenBank accession numbers can be found in Esposito and Prendini [76]. (SA=South America; CA=Central America; NA=North America; GA=Greater Antilles; JA=Jamaica; NLA=Northern Lesser Antilles; SLA=Southern Lesser Antilles; FL=Florida; BA=Bahamas and/or Turks and Caicos Islands).

| *Centruroides arctimanus* 7295 ^(GA)^ |
| --- |
| *Centruroides baergi* 2068 ^(NA)^ |
| *Centruroides balsasensis* 2010 ^(NA)^ |
| *Centruroides bani* 3302 ^(GA)^ |
| *Centruroides barbudensis* 6902 ^(NLA)^ |
| *Centruroides bicolor* 6112 ^(CA)^ |
| *Centruroides chamulaensis* 5248 ^(NA)^ |
| *Centruroides elegans* 1820 ^(NA)^ |
| *Centruroides elegans insularis* 9528 ^(NA)^ |
| *Centruroides exilicauda* 2147 ^(NA)^ |
| *Centruroides exilimanus* 9171 ^(CA)^ |
| *Centruroides farri* 5107 ^(JA)^ |
| *Centruroides flavopictus* 1823 ^(NA)^ |
| *Centruroides fulvipes* 8580 ^(NA)^ |
| *Centruroides griseus borinquensis* 10212 ^(GA)^ |
| *Centruroides griseus* 10209 ^(GA)^ |
| *Centruroides gracilis* 1550 ^(SA. CA, NA)^ |
| *Centruroides griseus* 1786 ^(GA)^ |
| *Centruroides guanensis* 6234 ^(GA, FL, BA)^ |
| *Centruroides hentzi* 1673 ^(FL)^ |
| *Centruroides hoffmanni* 3708 ^(NA)^ |
| *Centruroides infamatus* 4709 ^(NA)^ |
| *Centruroides insulanus* 5110 ^(JA)^ |
| *Centruroides koesteri* 6104 ^(CA)^ |
| *Centruroides limbatus* 1957 ^(CA)^ |
| *Centruroides limpidus* 1911 ^(NA)^ |
| *Centruroides luceorum* 9602 ^(GA)^ |
| *Centruroides margaritatus* 1787 ^(CA NA, GA)^ |
| *Centruroides mariaorum* 10228 ^(GA)^ |
| *Centruroides meisei* 6608 ^(NA)^ |
| *Centruroides meridionalis* 5214 ^(NA)^ |
| *Centruroides morenoi* 5118 ^(SA, CA, GA, JA)^ |
| *Centruroides nitidus taino* 2475 ^(GA)^ |
| *Centruroides nigrescens* 2005 ^(NA)^ |
| *Centruroides nigrimanus* 1808 ^(NA)^ |
| *Centruroides nigrovariatus* 2028 ^(NA)^ |
| *Centruroides noxius* 2071 ^(NA)^ |
| *Centruroides ochraceus* 7666 ^(NA)^ |
| *Centruroides ornatus* 2003 ^(NA)^ |
| *Centruroides platnicki* 9051 ^(BA)^ |
| *Centruroides pococki* 9040 ^(NLA, SLA)^ |
| *Centruroides rileyi* 6445 ^(NA)^ |
| *Centruroides sasae* 10239 ^(GA)^ |
| *Centruroides schmidti* 2070 ^(NA)^ |
| *Centruroides sculpturatus* 2169 ^(NA)^ |
| *Centruroides serrano* 9522 ^(NA)^ |
| *Centruroides sissomi* 7297 ^(NA)^ |
| *Centruroides suffusus* ^(NA)^ |
| *Centruroides testaceus arubensis* 9045 ^(SA)^ |
| *Centruroides testaceus* 9068 ^(SA)^ |
| *Centruroides tapachulaensis* 8578 ^(NA)^ |
| *Centruroides tecomanus* 2007 ^(NA)^ |
| *Centruroides thorelli* 5983 ^(NA)^ |
| *Centruroides tuxtla* 5250 ^(NA)^ |
| *Centruroides vittatus* 1721 ^(NA)^ |
| *Heteroctenus abudi* 10234 ^(GA)^ |
| *Heteroctenus bonettii* 2471 ^(GA)^ |
| *Heteroctenus garridoi* 10225 ^(GA)^ |
| *Heteroctenus junceus* 12613 ^(GA)^ |
| *Heteroctenus princeps* 12478 ^(GA)^ |
| *Ischnotelson guanambiensis* 9669 ^(SA)^ |
| *Ischnotelson peruassu* 9937 ^(SA)^ |
| *Jaguajir agamemnon* 9692 ^(SA)^ |
| *Jaguajir pintoi* 8278 ^(SA)^ |
| *Jaguajir rochae* 1775 ^(SA)^ |
| *Physoctonus debilis* 9678 ^(SA)^ |
| *Physoctonus striatus* 9681 ^(SA)^ |
| *Rhopalurus caribensis* 13167 ^(SA)^ |
| *Rhopalurus laticauda* 10046 ^(SA)^ |
| *Rhopalurus ochoai* 5504 ^(SA)^ |
| *Troglorhopalurus lacrau* 10211 ^(SA)^ |
| *Troglorhopalurus translucidus* 9668 ^(SA)^ |

**Details of *Micrathena* Spiders Analyses**

Methods

Sequences were downloaded from GenBank. *Micrathena* sequences that included two or more genes were used, unless a species was only represented by a single gene, in which case that species was also used (S13 Table). Outgroup taxa were chosen based on Dimitrov et al. [128] and Wheeler et al. [129], and distribution data is given in S13 Table. Alignments were made in MAFFT [114] for each gene individually on a server (<http://mafft.cbrc.jp/alignment/software/>), then the genes were concatenated in SequenceMatrix [116]. PartitionFinder2 [104] was used to simultaneously estimate the partitioning scheme and models of molecular evolution. We conducted Bayesian analyses to compare our results with those from McHugh et al. [22] and Magalhães and Santos [130]. Multiple BEAST 2 runs were conducted, with only constraints on the outgroups for calibration purposes or constraining ingroup relationships based on previous studies, until high ESS values (>200) were obtained. We continually got low ESS values for some of the partition 5 BMT rates. We tried using GTR and JC models for this partition, but the values did not improve. In the previous Bayesian analyses, we obtained 3 clades whose relationships to each other were uncertain. These were: Clade 1) *Micrathena banksi*, *M. militaris*, *M. sagittata*, *M. brevipes*, *M.* cf. *sexspinosa*, and *M. furcata*; Clade 2) *M. bimucronota*, *M. cubana*, *M. similis*, *M. saccata*, *M. macfarlanei*, *M. digitata*, *M. nigrichelis*, *M. plana*, and *M. schreibersi*; Clade 3) *M. annulata*, *M. forcipata*, *M. swainsoni*, *M. aureola*, *M. gracilis*, and *M. horrida*. The BEAST 2 tree that we chose to move forward with for BioGeoBEARS analysis was constructed by constraining the above relationships to ((clade 1, clade 3) clade 2), using BMT on all partitions (based on outcomes of previous runs), a log normal relaxed clock, and a Yule model of speciation. Constraints were placed on outgroup relationships based on Kallal and Hormiga [131], Gregorič et al. [132], and Wheeler et al. [129]. They were: 1) *Micrathena* and *Verrucosa arenata* 2) *Argiope* and *Gasteracantha* 3) *Argiope*, *Gasteracantha*, and *Guizygiella* 4) *Nephila* and Araneidae (all taxa used here exclusive of *Leucauge venusta*, *Metellina merianae*, *Phonognatha graeffei*, *Zygiella x-notata*, and *Steatoda bipunctata*) 5) *Zygiella* and *Phonognatha*. Calibrations were based on Dimitrov et al. [128] and were 1) a constraint including all taxa here exclusive of *Leucauge venusta*, *Metellina merianae*, and *Steatoda bipunctata*, using a uniform prior with a lower bound of 115 and an upper bound of 121, based on the fossil *Mesozygiella* and 2) a constraint of *L. venusta* and *M. merianae* using a uniform distribution with a lower bound of 125 and an upper bound of 135 based on the fossil *Macryphantes*. Given that *Micrathena* is an old lineage, we were able to examine the data in the context of all 252 models in BioGeoBEARS.

Results (S11 Fig, Fig 12)

For *Micrathena*, our MrBayes phylogeny differs slightly for outgroup vs. ingroup taxa compared to Dimitrov et al. [128], Wheeler et al. [129], and Garrison et al. [147]; however, given the taxon and gene sampling of our analyses compared to the others, this is to be expected. We used Wheeler et al. [129] for the outgroup relationships and calibration points, with the placement of *Guizygiella* based on Gregorič et al. [132] and Kallal [131] [i.e., ((((((*Verrucosa*+*Micrathena*) *Argiope* + *Gasteracantha* + *Guizygiella*) *Oarces*) *Nephila*) *Zygiella* + *Phonognatha*) *Leucauge*)].

Our results also differ for ingroup taxa compared to both Magalhães and Santos [130] and McHugh et al. [22]. Differences from the former are probably due to the authors including many more species, using only morphology, and having different outgroup taxa, whereas differences with the latter may derive from different outgroup taxa and the only tree that includes all species is from Cox1 only and is not consistent with the other trees presented in the paper. McHugh et al. [22] do not discuss these incongruencies or explain why they chose the Cox1 gene tree as their preferred topology for subsequent analyses. Additionally, we were unable to recover the topology of McHugh et al. [22] using Cox1. Rather than relying on these studies and their results, we chose to move forward using our own results instead of constraining the relationships to either of the previous studies as the low support values give us little reason to do so. The outgroup relationships in the BEAST 2 analysis recovered *Micrathena* older (~20 mya) than in the results of McHugh et al. [22], as expected from differences in calibrations.

BioGeoBEARS analyses indicate the best model based on the AICc weights is DIVALIKE + J B2a (Table 2), or a DIVALIKE model that includes founder event dispersal with the following constraints: dispersal from South America to the Greater Antilles via the GAARlandia landspan, with dispersal across the IoP/CAS occurring as early as 23 mya, and dispersal is affected by distance between landmasses. Ancestral range estimation (S11 Fig, Fig 12) indicates at least 6 separate dispersal events to the Caribbean from the mainland. Although a model that represents the GAARlandia land bridge was chosen as a means of dispersal over other models, nearly all dispersals to the Greater Antilles occur from North America, including those that occur during the 35–32 mya period when GAARlandia is proposed to have existed. It should be noted that in McHugh et al.’s [22] Lagrange analyses, they did not include the entire distributions of the species, but instead only used the areas from which they had collected as the “distributions”. It is unclear why this was done and may be responsible for the differences in our conclusions.

Six dispersal events from the mainland to the Caribbean is quite high for poor dispersers [22]. However, analyses indicate they are a rather old group [22], thus having more time to disperse. Most dispersal events appear to have occurred from North America with fewer from South America, in contrast to the findings of McHugh et al. [22]. However, missing taxa from Central America and South America may affect the outcome of both analyses. Nevertheless, the occurrence of multiple dispersal events to the Caribbean is congruent with the results of Magalhães and Santos [130].

**S10 Fig. Phylogeny and ancestral range estimation for *Micrathena* spiders.**

BioGeoBEARS phylogram corresponding to the DIVALIKE + J B2a model (Table 1). Pie charts represent the probabilities of each possible geographical area before and after each split. Colors correspond to Figure 1. SA = South America; CA = Central America; NA = North America; GA = Greater Antilles; JA = Jamaica; SLA = Southern Lesser Antilles; NLA = Northern Lesser Antilles. Photo by S. Crews.


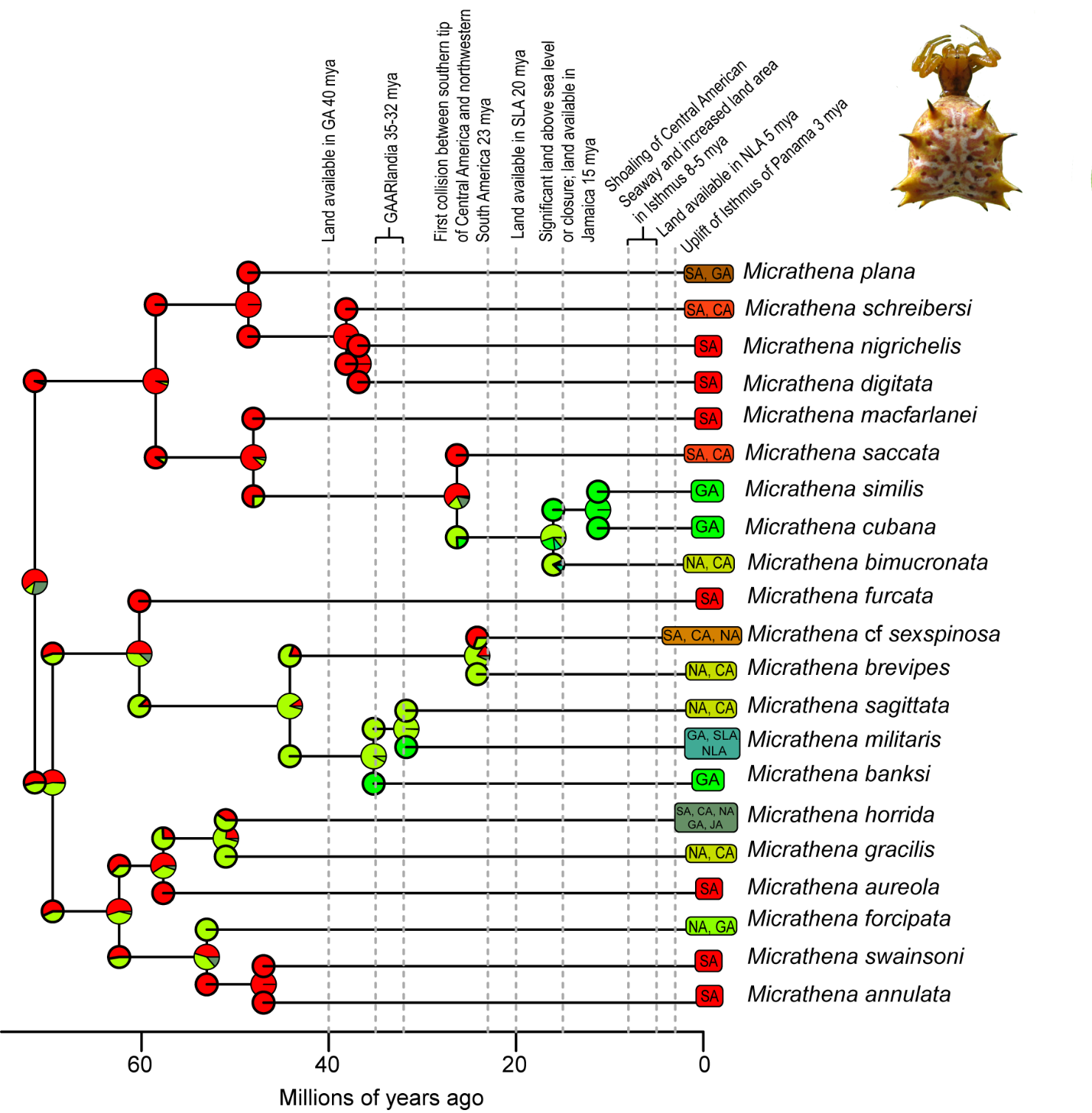


**S13 Table.** **Sequences for *Micrathena* analyses.** GenBank accession numbers for *Micrathena* spiders and outgroups used in analyses. All *Micrathena* sequences were used in the Bayesian analyses along with ougtroups, and those taxa with at least one asterisk were used in the BEAST 2 analysis, and those with two asterisks were also used in BioGeoBEARS analyses. Distributions considered for each species used in the BioGeoBEARS analyses are provided. (SA=South America; CA=Central America; NA=North America; GA=Greater Antilles; Ja=Jamaica; SLA=Southern Lesser Antilles; NLA=Northern Lesser Antilles; BA=Bahamas and Turks and Caicos Islands; FL=Florida)

| Species | 16S | COI | ITS | Additional File References |
| --- | --- | --- | --- | --- |
| *Micrathena annulata* 007** ^(SA)^ | - | KJ157272 | - | [22] |
| *Micrathena aureola* 009** ^(SA)^ | - | KJ157249 | - | [22] |
| *Micrathena banksi* CU 784750** ^(GA)^ | KJ156991 | KJ157215 | KJ157104 | [22] |
| *Micrathena banksi* CU 784976 | KJ156993 | KJ157217 | KJ157105 | [22] |
| *Micrathena banksi* CU 785101 | KJ156994 | KJ157220 | KJ157106 | [22] |
| *Micrathena banksi* CU 785175 | KJ156995 | KJ157219 | KJ157107 | [22] |
| *Micrathena banksi* CU 787933 | KJ156996 | KJ157218 | KJ157108 | [22] |
| *Micrathena bimucronata* 123** ^(CA, NA)^ | - | KJ157236 | - | [22] |
| *Micrathena brevipes* 121** ^(CA, NA)^ | - | KJ157223 | - | [22] |
| *Micrathena* nr. *Sexspinosa*** ^(SA, CA, NA)^ | KJ157091 | KJ157222 | - | [22] |
| *Micrathena cubana* CU 784355** ^(GA)^ | KJ156997 | KJ157224 | KJ157109 | [22] |
| *Micrathena cubana* CU 784820 | KJ156998 | KJ157225 | KJ157110 | [22] |
| *Micrathena cubana* CU 785048 | KJ156999 | KJ157226 | KJ157111 | [22] |
| *Micrathena digitata* 017** ^(SA)^ | - | KJ157238 | - | [22] |
| *Micrathena forcipata* CU 784425** ^(NA, GA)^ | KJ157002 | KJ157256 | KJ157113 | [22] |
| *Micrathena forcipata* DR 782434 | KJ157005 | KJ157260 | KJ157114 | [22] |
| *Micrathena forcipata* DR 784362 | KJ157006 | KJ157264 | KJ157115 | [22] |
| *Micrathena forcipata* DR 784447 | KJ157007 | KJ157261 | KJ157117 | [22] |
| *Micrathena forcipata* DR 785054 | KJ157008 | KJ157263 | KJ157118 | [22] |
| *Micrathena forcipata* DR 785682 | KJ157010 | KJ157262 | KJ157120 | [22] |
| *Micrathena forcipata* DR 787150 | KJ157012 | KJ157267 | KJ157121 | [22] |
| *Micrathena forcipata* DR 787153 | KJ157013 | KJ157269 | KJ157122 | [22] |
| *Micrathena forcipata* DR 787210 | KJ157014 | KJ157268 | KJ157123 | [22] |
| *Micrathena forcipata* DR 787243 | KJ157015 | KJ157270 | KJ157124 | [22] |
| *Micrathena furcata* 037** ^(SA)^ | - | KJ157242 | - | [22] |
| *Micrathena gracilis* NA 00000889A** ^(CA, NA)^ | KJ157082 | KJ157251 | KJ157190 | [22] |
| *Micrathena gracilis* NA 00000935A | KJ157083 | KJ157254 | KJ157191 | [22] |
| *Micrathena gracilis* NA 00000954A | KJ157084 | KJ157252 | KJ157192 | [22] |
| *Micrathena gracilis* NA 00000984A | KJ157086 | KJ157253 | KJ157194 | [22] |
| *Micrathena gracilis* NA 00000988A | KJ157087 | KJ157255 | KJ157195 | [22] |
| *Micrathena horrida* CU 784351** ^(SA, CA, NA, GA, JA)^ | KJ157016 | KJ157243 | KJ15712 | [22] |
| *Micrathena horrida* CU 784751 | KJ157017 | KJ157246 | KJ157126 | [22] |
| *Micrathena horrida* CU 787913 | KJ157018 | KJ157247 | KJ157127 | [22] |
| *Micrathena horrida* CU 787919 | KJ157019 | KJ157244 | KJ157128 | [22] |
| *Micrathena macfarlanei* 054** ^(SA)^ | - | KJ157241 | - | [22] |
| *Micrathena militaris* DR 784363 | KJ157022 | KJ157293 | KJ157130 | [22] |
| *Micrathena militaris* DR 784403 | KJ157023 | KJ157298 | KJ157131 | [22] |
| *Micrathena militaris* DR 784448 | KJ157025 | KJ157294 | KJ157133 | [22] |
| *Micrathena militaris* DR 784503 | KJ157027 | KJ157300 | KJ157135 | [22] |
| *Micrathena militaris* DR 784566 | KJ157029 | KJ157296 | KJ157137 | [22] |
| *Micrathena militaris* DR 784721 | KJ157031 | KJ157310 | KJ157139 | [22] |
| *Micrathena militaris* DR 784759 | KJ157032 | KJ157277 | KJ157140 | [22] |
| *Micrathena militaris* DR 784772 | KJ157034 | KJ157287 | KJ157142 | [22] |
| *Micrathena militaris* DR 785080 | KJ157038 | KJ157274 | KJ157146 | [22] |
| *Micrathena militaris* DR 785169 | KJ157041 | KJ157290 | KJ157149 | [22] |
| *Micrathena militaris* DR 785173 | KJ157042 | KJ157314 | KJ157150 | [22] |
| *Micrathena militaris* DR 785174 | KJ157043 | KJ157292 | KJ157151 | [22] |
| *Micrathena militaris* DR 785208 | KJ157045 | KJ157297 | KJ157152 | [22] |
| *Micrathena militaris* DR 785219 | KJ157046 | KJ157286 | KJ157153 | [22] |
| *Micrathena militaris* DR 785273 | KJ157048 | KJ157275 | KJ157155 | [22] |
| *Micrathena militaris* DR 785280 | KJ157049 | KJ157315 | KJ157156 | [22] |
| *Micrathena militaris* DR 785312 | KJ157050 | KJ157280 | KJ157157 | [22] |
| *Micrathena militaris* DR 785401 | KJ157051 | KJ157276 | KJ157158 | [22] |
| *Micrathena militaris* DR 785402 | KJ157052 | KJ157285 | KJ157159 | [22] |
| *Micrathena militaris* DR 785502** ^(GA, SLA, NLA)^ | KJ157055 | KJ157301 | KJ157161 | [22] |
| *Micrathena militaris* DR 785512 | KJ157056 | KJ157316 | KJ157162 | [22] |
| *Micrathena militaris* DR 785524 | KJ157057 | KJ157311 | KJ157163 | [22] |
| *Micrathena militaris* DR 785527 | KJ157058 | KJ157279 | KJ157164 | [22] |
| *Micrathena militaris* DR 785563 | KJ157059 | KJ157295 | KJ157165 | [22] |
| *Micrathena militaris* DR 785604 | KJ157060 | KJ157288 | KJ157166 | [22] |
| *Micrathena militaris* DR 785706 | KJ157061 | KJ157278 | KJ157167 | [22] |
| *Micrathena militaris* DR 785722 | KJ157062 | KJ157283 | KJ157169 | [22] |
| *Micrathena militaris* DR 785729 | KJ157063 | KJ157284 | KJ157170 | [22] |
| *Micrathena militaris* DR 785743 | KJ157064 | KJ157282 | KJ157171 | [22] |
| *Micrathena militaris* DR 787068 | KJ157066 | KJ157299 | KJ157172 | [22] |
| *Micrathena militaris* DR 787106 | KJ157067 | KJ157289 | KJ157174 | [22] |
| *Micrathena militaris* DR 787148 | KJ157068 | KJ157291 | KJ157175 | [22] |
| *Micrathena militaris* PR 392672 | KJ157074 | KJ157302 | KJ157181 | [22] |
| *Micrathena militaris* PR 392677 | KJ157075 | KJ157303 | KJ157182 | [22] |
| *Micrathena militaris* PR 782048 | KJ157076 | KJ157307 | KJ157183 | [22] |
| *Micrathena militaris* PR 782126 | KJ157076 | KJ157307 | KJ157183 | [22] |
| *Micrathena militaris* PR 782153 | KJ157078 | KJ157306 | KJ157185 | [22] |
| *Micrathena militaris* PR 782174 | KJ157079 | KJ157304 | KJ157186 | [22] |
| *Micrathena militaris* PR 782201 | KJ157080 | KJ157305 | KJ157187 | [22] |
| *Micrathena nigrichelis* 056** ^(SA)^ | - | KJ157239 | - | [22] |
| *Micrathena plana* 062** ^(SA, GA)^ | - | KJ157240 | - | [22] |
| *Micrathena saccata* 076** ^(SA, CA)^ | - | KJ157237 | - | [22] |
| *Micrathena sagittata* NA 00000833A** ^(CA, NA)^ | KJ157081 | KJ157221 | KJ157189 | [22] |
| *Micrathena schreibersi* SA 00000936A** ^(SA, CA)^ | KJ157090 | KJ157318 | KJ157198 | [22] |
| *Micrathena schreibersi* SA 00002357A | KJ157319 | KJ157319 | KJ157199 | [22] |
| *Micrathena similis* DR 785024** ^(GA)^ | KJ157093 | KJ157228 | KJ157200 | [22] |
| *Micrathena similis* DR 785496 | KJ157094 | KJ157232 | KJ157201 | [22] |
| *Micrathena similis* DR 787265 | KJ157095 | KJ157233 | KJ157202 | [22] |
| *Micrathena similis* DR 787308 | KJ157097 | KJ157229 | KJ157204 | [22] |
| *Micrathena similis* DR 787318 | KJ157099 | KJ157234 | KJ157207 | [22] |
| *Micrathena similis* DR 787320 | KJ157100 | KJ157230 | KJ157208 | [22] |
| *Micrathena similis* DR 787322 | KJ157100 | KJ157230 | KJ157208 | [22] |
| *Micrathena swainsoni* 090** ^(SA)^ | - | KJ157320 | - | [22] |
| *Argiope lobata** | KJ156988 | KJ957971 | KJ157103 | [22, 207] |
| *Gasteracantha cancriformis* DR 787198* | KJ156989 | KJ157212 | - | [22] |
| *Gasteracantha cancriformis* PR 782149 | KJ156990 | KJ157214 | - | [22] |
| *Guizygiella nadleri** | KR526577 | - | KR526529 | [132] |
| *Guizygiella salta* | KR526404 | - | KR526530 | [132] |
| *Nephila clavipes** | FJ525361 | FJ525328 | KR526539 | [132, 208] |
| *Oarces* sp. SP000013* | JN010171 | - | - | [128] |
| *Verrucosa arenata** | FJ525364 | FJ525331 | - | [208] |
| *Zygiella x-notata** | KR526424 | KR526601 | **-** | [132] |
| *Zygiella x-notata* FAPDNA003 | EU003251 | EU003311 | - | [209] |
| *Leucauge venusta** | FJ607457 | FJ607568 | - | [210] |
| *Metellina merianae** | EU003270 | KX039254 | KR526536 | [132, 209, 211] |
| *Phonognatha graeffei** | FJ607469 | FJ607469 | KR526547 | [210] |
| *Steatoda bipunctata** | AY230951 | AY231057 | KR526550 | [132, 212] |

**Details of *Spintharus* Spiders Analyses**

Methods

The aligned *Spintharus flavidus* dataset was downloaded from the Supplementary Information of Dziki et al. [52]. We used PartitionFinder2 [104] to simultaneously estimate partitioning scheme and models of evolution for the partitions. We used bPTP [58] to determine species boundaries as the authors indicated that this group represents a species complex rather than a single, widespread species.

For BEAST 2, we used the authors’ calibrations of a fossil of *Spintharus* from Dominican amber and Spintharinae based on a Baltic amber fossil. We pruned taxa in Mesquite [110] because we were treating hypothesized species as single terminals. We ran multiple analyses, using both log normal and exponential prior distributions. We also moved the Dominican amber fossil calibration from the node that includes Cuba and Hispaniola to the one that only includes Hispaniola and used both log normal relaxed and strict clock models as well as BMT. Placing the calibration on only the Hispaniolan putative species, pushed the dates for the previous nodes further back to times we considered unreasonable. In the end, based on the ESS values and plots recovered in Tracer 1.6, we used the following parameters to obtain a tree for subsequent analyses: BMT for each partition, a strict clock for partitions 1–4, and a log normal relaxed clock for partition 5, a Yule process of speciation, a calibration constraint with an exponential prior with a mean of 5 and an offset of 15 for the Cuban and Hispaniolan hypothesized *Spintharus* species, and a Spintharinae exponential prior for the node of *Spintharus* + *Episinus* with a mean of 15 and an offset of 44. We tested all 252 hypotheses in BioGeoBEARS. Distributions used in these analyses are given in S14 Table.

Results (S12 Fig, Fig 13)

Despite using the authors’ [52] dataset, we were unable to recover the same topology in our MrBayes analyses as shown in their Figure 1. The primary difference is in the clade that includes Jamaica, Puerto Rico, and Lesser Antilles specimens: We recovered Grenada and St. Lucia specimens as basal, but in Dziki et al. [52], the Jamaica specimens are basal. This clade is well-supported in their analysis (PP=97) and is slightly lower support in our own (PP=93.2). The rest of the topology is congruent with Dziki et al. [52], and although support values vary, clades are generally well-supported. Differences may be due to how the data were partitioned or how long the analyses were run. We chose to continue using the tree from our own analyses. Our bPTP analyses resulted in a slightly different set of putative species, using one less “species” than the authors for our biogeographic analyses.

The BEAST 2 analysis recovered the same relationships as the MrBayes analysis, although the *Spintharus* clade exclusive of the *Spintharus* MX + *Spintharus* SEUS clade was not supported (PP=0.65). The low support values could be caused by missing samples, particularly from the mainland. The age of the split recovered for Colombia *Spintharus* is much older (20 my) than that recovered in Dziki et al. [52]; however, our *Spintharus* clade is 5 my younger than their date for the same node (their Figure 2), despite using the same calibrations and speciation prior. This could be due to the partitioning, the clock model, or that they used all samples in their analyses whereas our best practices included a single exemplar from each hypothesized species.

Our BioGeoBEARS analyses revealed that the favored model based on AICc weights is DIVALIKE + J A1b (Table 2), or a DIVALIKE model that includes founder event dispersal with the following constraints: the GAARlandia land bridge did not play a role in dispersal from South America to the Greater Antilles, dispersal across the IoP/CAS only occurred within the last 3 my, and dispersal is not affected by distance between geographic areas. According to the ancestral range estimation (S12 Fig, Fig 13), there has been only a single dispersal event into the Caribbean from North America, with the Greater Antilles as a dispersal point for Jamaica, the Northern Lesser Antilles, and the Southern Lesser Antilles.

**S11 Fig. Ancestral range estimation for *Spintharus* spiders.**

BioGeoBEARS phylogram corresponding to the DIVALIKE + J A1b model (Table 1). Pie charts represent the probabilities of each possible geographical area before and after each split. Colors correspond to Figure 1. SA = South America; CA = Central America; NA = North America; GA = Greater Antilles; JA = Jamaica; SLA = Southern Lesser Antilles; NLA = Northern Lesser Antilles. Photo by Judy Gallagher.


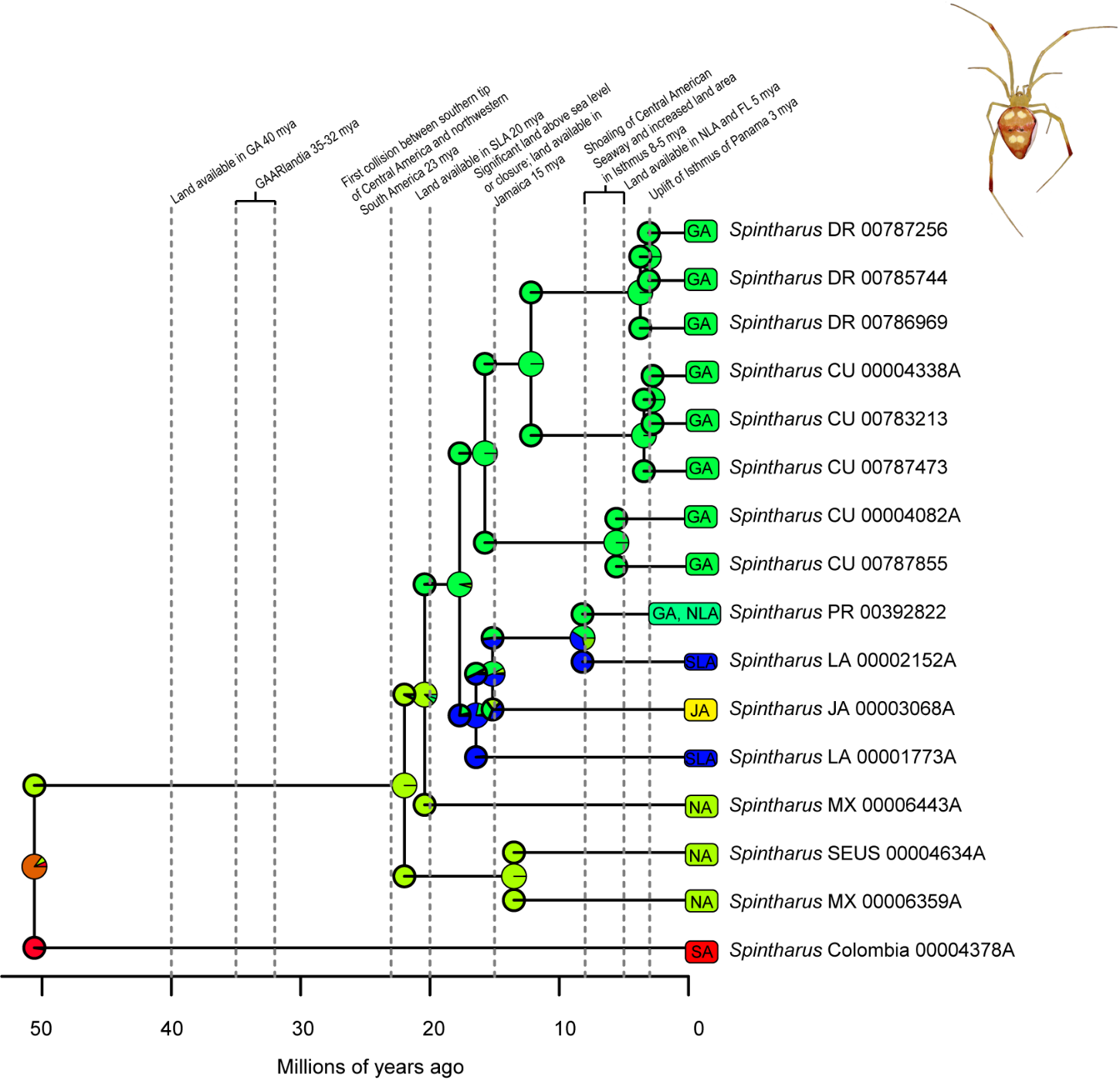


**S14 Table.** **Sequences for *Spintharus* analyses.** *Spintharus* “species” used for BEAST 2 and BioGeoBEARS analyses and the corresponding distributions that were used in BioGeoBEARS for the hypothesized species. GenBank accession numbers and species used in MrBayes analyses can be found in Dziki et al [52] (SA=South America; NA=North America; SLA = Southern Lesser Antilles; JA = Jamaica; GA = Greater Antilles; NLA = Northern Lesser Antilles).

| Hypothesized species | Corresponding clade from [52] Fig. 1 |
| --- | --- |
| *Spintharus* Columbia 00004378A ^(SA)^ | Colombia |
| *Spintharus* SEUS 00004634A ^(NA)^ | 2 |
| *Spintharus* MX 00006359A ^(NA)^ | 1 |
| *Spintharus* MX 00006443A ^(NA)^ | 3 |
| *Spintharus* LA 00001773A ^(SLA)^ | 5 |
| *Spintharus* JA 00003068A ^(JA)^ | 4 |
| *Spintharus* LA 00002152A ^(SLA)^ | 6 |
| *Spintharus* PR 00392822 ^(GA, NLA)^ | 7 |
| *Spintharus* CU 00004082A ^(GA)^ | 8 |
| *Spintharus* CU 00787855 ^(GA)^ | 9, 10 |
| *Spintharus* DR 00786969 ^(GA)^ | 12 |
| *Spintharus* DR 00787256 ^(GA)^ | 13 |
| *Spintharus* DR 00785744 ^(GA)^ | 11 |
| *Spintharus* CU 00787473 ^(GA)^ | 14 |
| *Spintharus* CU 00004338A ^(GA)^ | 15 |
| *Spintharus* CU 00783212 ^(GA)^ | 16 |

*in this analysis, North America was used for the spiders found in Florida and no FL geological constraints because the spiders likely colonized Florida from the north and west, rather than being recent arrivals from the Caribbean.

***Selenops* Spiders Analyses**

In Crews and Gillespie [21], only 1 Cuban endemic species was used in the analyses because we were unable to collect on that island at the time. Since then, we obtained specimens from the CarBio project that included more Cuban endemics as well as additional specimens from other localities. Some of the specimens from Crews and Gillespie [21] were missing data/genes, so we also included those as well as new taxa in hopes of providing better resolution to any nodes that were not well-supported in previous analyses. The former were sent as extracts from the National Museum of Natural History. The latter were extracted using a Qiagen DNeasy spin column kit for animal tissues following the manufacturer’s protocol. The specimens are deposited according to Crews and Gillespie [21], and the tissues and extracts are stored at the Center for Comparative Genomics (CCG) facility at the California Academy of Sciences (S15 Table). PCR primers and protocols are given in S16 Table. PCR products were cleaned using ExoSAP-IT (ThermoFisher Scientific) and cycle sequenced using the protocol in S16 Table. Sequences were read on an ABI 3130 Sequencer in the CCG lab at the California Academy of Sciences. Sequences were edited in SeqTrace [133] and concatenated in SequenceMatrix [116].

Both MrBayes [105] and RAxML [134] were run on the *Selenops* dataset. In MrBayes, each gene was analyzed separately and a concatenated run was conducted. Because the datasets contained so many terminals (concatenated with 925), it was difficult to run them long enough to obtain satisfactory ESS results and traces. To make the analyses more tractable, we only used taxa with data for 2 or more genes (unless a certain species was only represented by a single gene) and removed redundant haplotypes with CD-HIT [135, 136, 137], leaving 319 terminals. RAxML was called as follows: /raxml/8.2.9/raxmlHPC-HYBRID -T 4 -n result -N autoMRE -s infile.txt -q part.txt -p 12345 -x 12345 -c 25 -k -f a -m GTRCAT. Two runs of 100 million generations were conducted in MrBayes. Input files from all analyses are available from the authors; in the additional files on figtree, we only included infiles that we discuss and use for subsequent BEAST 2 and BioGeoBEARS analyses. All sequences are available on GenBank and in Crews and Gillespie [21].

In Crews and Gillespie [21], outgroup taxa consisted of sparassids and ctenids, including a taxon now placed in the newly erected family Viridasiidae [138]. For the analyses conducted here, sparassids were not included as we now know that they are not closely related to selenopids [139] and that ctenids and viridasiids are sister to Selenopidae [129, 138, 139]. Other outgroups to *Selenops* included other genera in the family, as in Crews and Gillespie [21].

For BEAST 2 analyses we tried many (>50) different prior calibration schemes. These included: using the African selenopid outgroups and a biogeographic date to constrain the separation of African and American *Selenops*, using a molecular rate [140], and using fossil calibrations – *Selenops* is known from Dominican amber though despite microCT scanning [5], the placement of the fossil is uncertain, so the constraint was put on multiple nodes. *Selenops* is also known from Chiapas amber (S. Crews has seen the specimen at an amber museum in Chiapas, Mexico, but was not allowed to photograph or look closely at the specimen). Additionally, selenopids are known from Baltic amber (44 myo) and possibly from an impression fossil from the Green River Formation of Wyoming (49-50 myo) [141]; however, the former cannot be placed beyond family as they are juveniles. Both fossils indicate the Selenopidae are a very old group, and the latter indicates that the genus has likely been in the New World a long time. We tried various combinations of these calibration points and the calibrations on various nodes as well as different constraint trees because basal relationships are uncertain (see [21] and results here), as well as birth-death vs. Yule processes. We finally settled on the following parameters: No Old World taxa, an HKY model for partitions 1–7 and JC for partition 8, a relaxed log normal clock for partitions 1, 4, and 6, an exponential clock on partition 2, and a normal distribution on partition 3 with a clock rate of 0.0199, a strict clock on partitions 5, 7, and 8, a Yule model of speciation, with uniform priors (lower bound 22, upper bound 26) for the Chiapas amber fossil on the node with *Selenops huetocatl*, *S. malinalxochitl*, *S. abyssus*, *S.* sp. sel_1007, *S. gracilils*, *S. actophilus*, *S. nesophilus*, *S. debilis*, *S. petenajtoy*, *S. bifurcatus*, and *S. mexicanus*, a uniform prior (lower bound 15, upper bound 20) for the Dominican amber fossil placed on a node of *S. insularis*, *S. simius*, *S. souliga*, *S. marcanoi*, *S. phaselus*, *S. bocacanadensis*, *S. enriquillo*, *S. bani*, *S. vinalesi*, *S. submaculosus*, *S. baweka*, *S. candidus*, *S. petrunkevitchi*, *S. wilmotorum*, *S. wilsoni*, *S. pensilis*, *S. caonabo*, *S. morro*, *S. guerrero*, *S. oviedo*, *S. denia*, *S. kalinago*, *S. aequalis*; finally, we also used a biogeographic date (uniform prior, lower bound 0, upper bound 1) for the Turks and Caicos Islands since they have an endemic species and have likely only been habitable for 500 ky [142]. The results of this run were used to conduct 5 additional analyses, and these were used for subsequent BioGeoBEARS analyses. The five following runs had the aforementioned constraints in addition to or minus the following: 1) no Chiapas amber fossil constraint; 2) constraining 2 clades – one of Caribbean and one of everything else; 3) constraining 3 clades – a Caribbean clade, a South American clade and a Central America/North America clade; 4) constraining (((Caribbean+mainland South America) ABC Islands + Trinidad and Tobago) Mexico-Central America); 5) (((Caribbean + Mexico-Central America) South America)). Four of these trees were used for subsequent BioGeoBEARS analyses, but the dates were too young to include a test of the GAARlandia hypothesis. When we included Old World taxa, this pushed the dates back further and allowed us to test models including GAARlandia; however, we don’t have much faith in this arrangement because it dated the Old World sister group as having a split we deem unreasonable. This is due to missing data. We decided to use both an older tree and a younger tree for further analyses as both seem plausible. Our preferred tree is number 2 above, and this is the primary one we discuss and for which we have uploaded the input file and the consensus tree.

Results (S13 Fig, Fig 14)

In all phylogenetic analyses (e.g., single gene vs. concatenated, Bayesian vs. likelihood), resulting topologies were very similar. There were a few differences in relationships among outgroup taxa in some of the single gene analyses, but these weren’t supported. Only one of two MrBayes runs (100 million generations) resulted in high ESS values. Among the New World *Selenops* species, there are either 2 or 3 well-supported clades. One consisting of taxa from the ABC (Aruba, Bonaire, and Curaçao) islands and Trinidad and Tobago, with close relatives on the mainland (Northern South American clade); AND/OR a clade of North American, Central American, and South American taxa (with the South American clade containing one species found in the Southern Lesser Antilles); a clade with Caribbean species. Support for 2 clades is higher than for 3 clades. The Central America/North America clade is well-supported in all analyses and typically divided into 2 smaller, well-supported clades. One of these contains *S. mexicanus* and the closely related species *S. gracilis* and *S. malinalxochitl*, and the other consists of all other Central American and North American species.

Support for the Caribbean clade is low in all analyses, and basal relationships within this clade are poorly supported. This was also found in Crews and Gillespie [21]. They attributed this to an artefact of phylogenetics in which deep, short branches, such as those that occur when there are species radiations, may be difficult if not impossible to resolve [148, 149]. This hypothesis remains supported as the addition of molecular data and species did not improve resolution. Taxa from the Yucatan are still missing from the dataset, and the dataset is largely mitochondrial with a small nuclear fragment. Within the Caribbean some groups of species are supported, but their relationships to one another are unstable. *Selenops vinalesi* and *S. aissus* are usually well-supported as being sister taxa; *S. denia*, *S. guerrero*, and *S. baweka* are well-supported. Another clade supported by morphology as well as molecular data [5] consists of *S. souliga*, *S. kalinago*, *S. morro*, *S. simius*, and *S. submaculosus.* A clade of Jamaican species is also well-supported in all analyses.

The BioGeoBEARS analyses using the tree with the older date allowing the testing of GAARlandia indicated that the favored model was DIVALIKE+J B1a (Table 2), or a DIVALIKE model that includes founder event dispersal with the following constraints: distance between geographic areas does not affect dispersal, dispersal from South America to the Greater Antilles is inconsistent with GAARlandia, and dispersal over the IoP/CAS began as early as 23 mya. The ancestral range estimation (S13 Fig, Fig 14) indicates a South American and Greater Antillean origin, but because the Greater Antilles did not exist at this time, the authors attribute this to: 1) land available before 40 my that does not appear in the geologic record; or 2) missing species, either due to extinction or incomplete sampling. Two dispersal events to the Caribbean are indicated, one from South America throughout the Southern Lesser Antilles, and another likely from South America just after 40 mya. There is no evidence of dispersal to the mainland from the islands.

The suite of analyses of the 4 younger trees with different relationship constraints testing only the CAS/IoS hypotheses all indicated that the best model based on the AICc weights was DIVALIKE B1a (Table 2), similar to the above, with the following constraints: distance between geographic areas does not affect dispersal, and dispersal over the IoP/CAS began as early as 23 mya; however, this model does not include founder event dispersal. The ancestral range estimation ((S13 Fig, Fig 14) is nearly the same among the topologies, with the same dispersal patterns occurring at later times. Based on fossils, we know the group is very old and has been in North America for a long time, and the Caribbean taxa are certainly unique with a so-far elusive origin.

**S12 Fig. Ancestral range estimation for *Selenops* spiders using older dated phylogenies.**

BioGeoBEARS phylogram corresponding to the DIVALIKE + B1a model (Table 1). Pie charts represent the probabilities of each possible geographical area before and after each split. Colors correspond to Figure 1. SA = South America; CA = Central America; NA = North America; GA = Greater Antilles; JA = Jamaica; SLA = Southern Lesser Antilles; NLA = Northern Lesser Antilles. Photo by S. Crews.

_
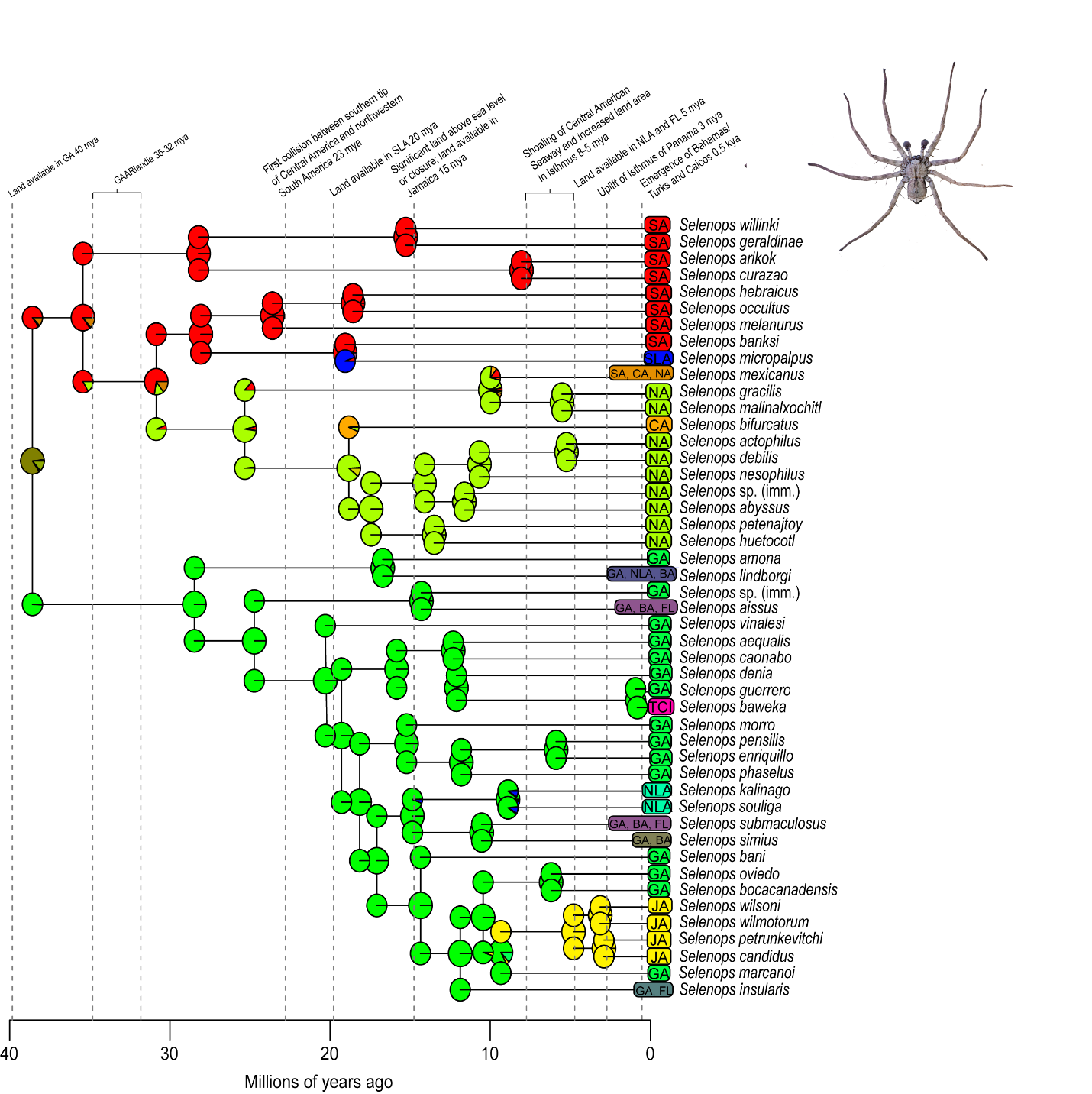
_

**S13 Fig. Ancestral range estimation for *Selenops* spiders using more recent dated phylogenies.**

BioGeoBEARS phylogram corresponding to the DIVALIKE + B1a model (Table 1). The first tree shows the single most probable geographical range at each node pre- and post-split. Pie charts in the second tree represent the probabilities of each possible geographical area before and after each split. Colors correspond to Figure 1. SA = South America; CA = Central America; NA = North America; GA = Greater Antilles; JA = Jamaica; SLA = Southern Lesser Antilles; NLA = Northern Lesser Antilles. Photo by S. Crews.


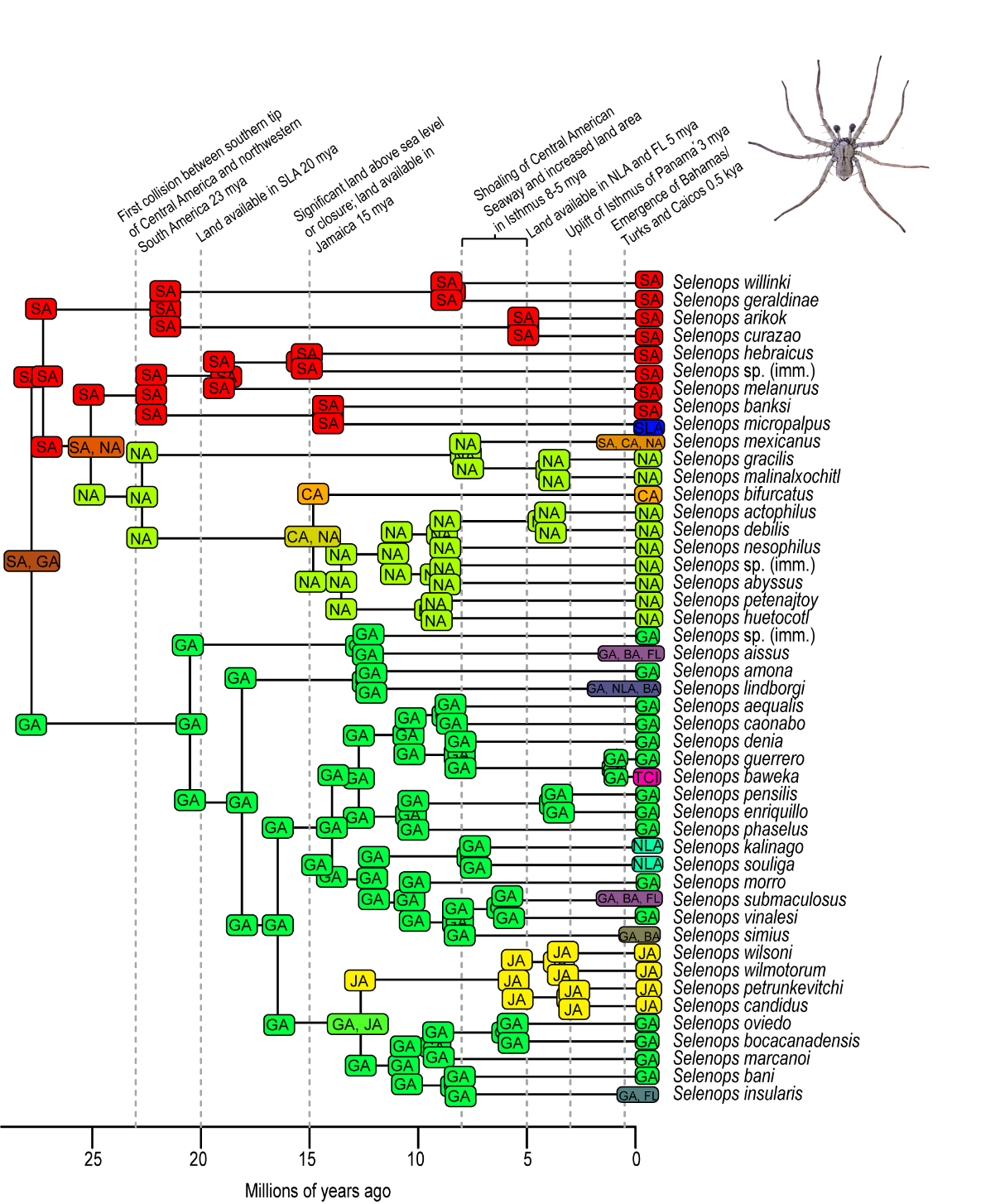


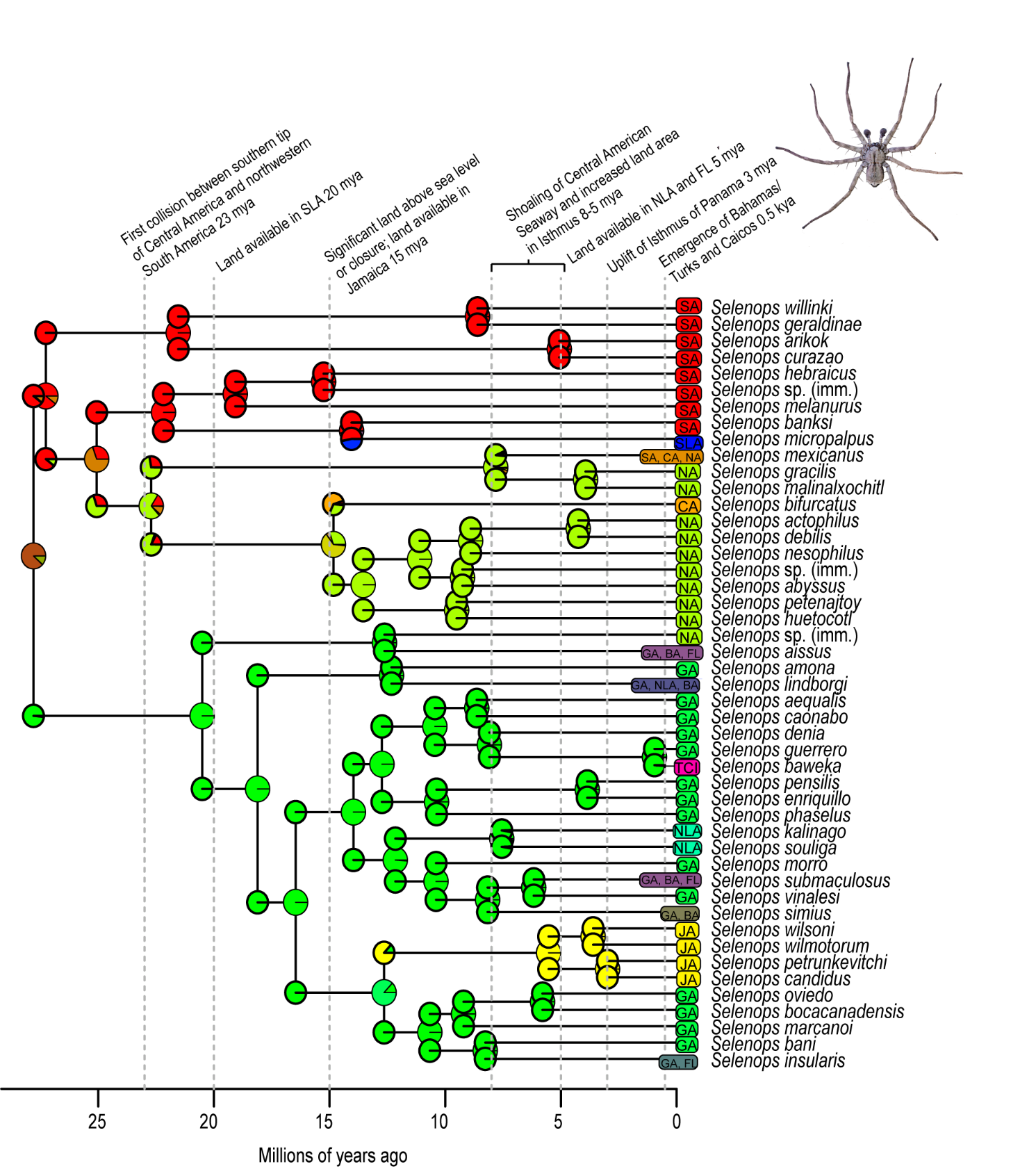


**S14 Fig. Selenopidae tree from the Bayesian analysis using no redundant haplotypes.** Species have been pruned to single terminals.


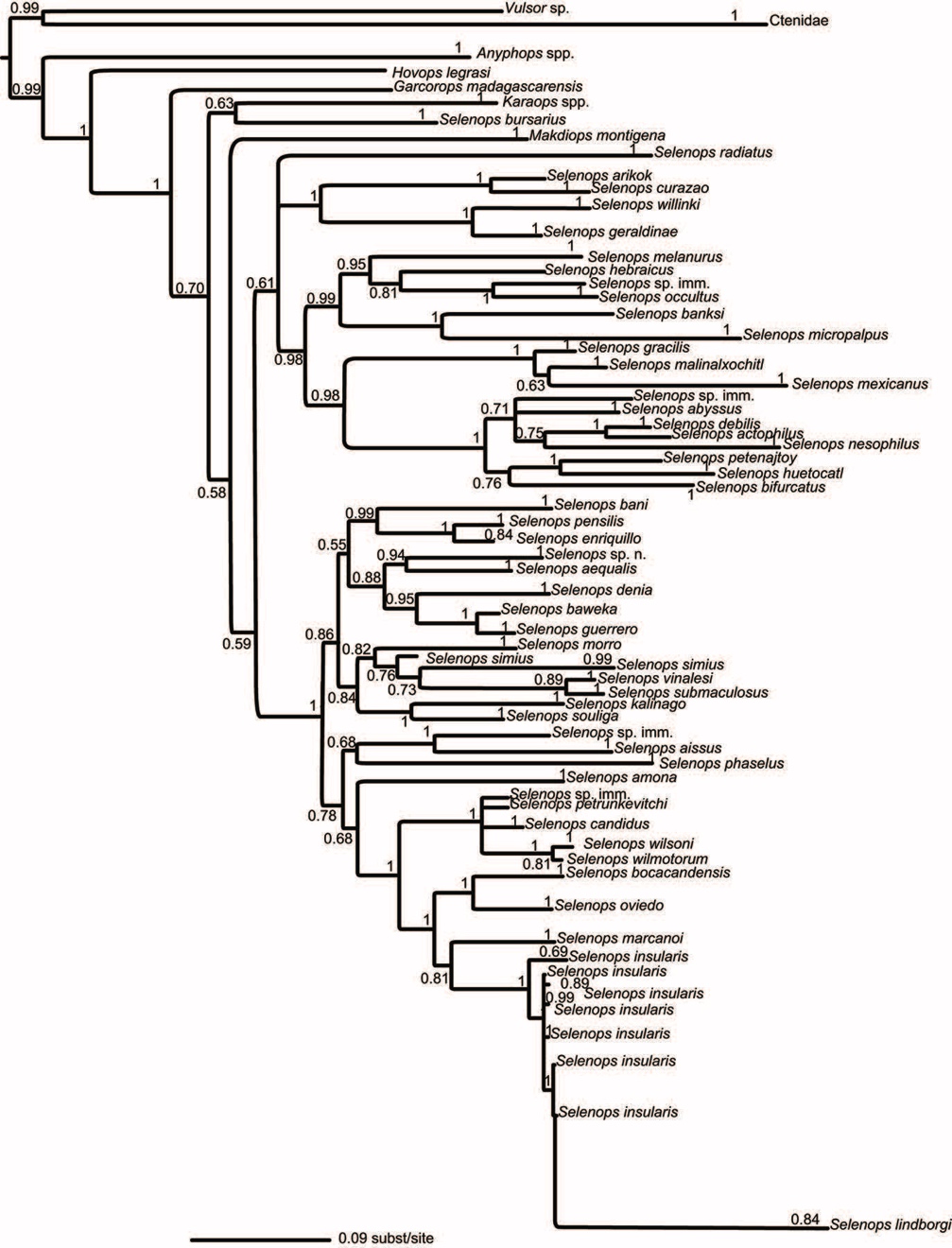


**S15 Fig. Selenopidae tree from the RAxML analysis using no redundant haplotypes.** Species have been pruned to single terminals.


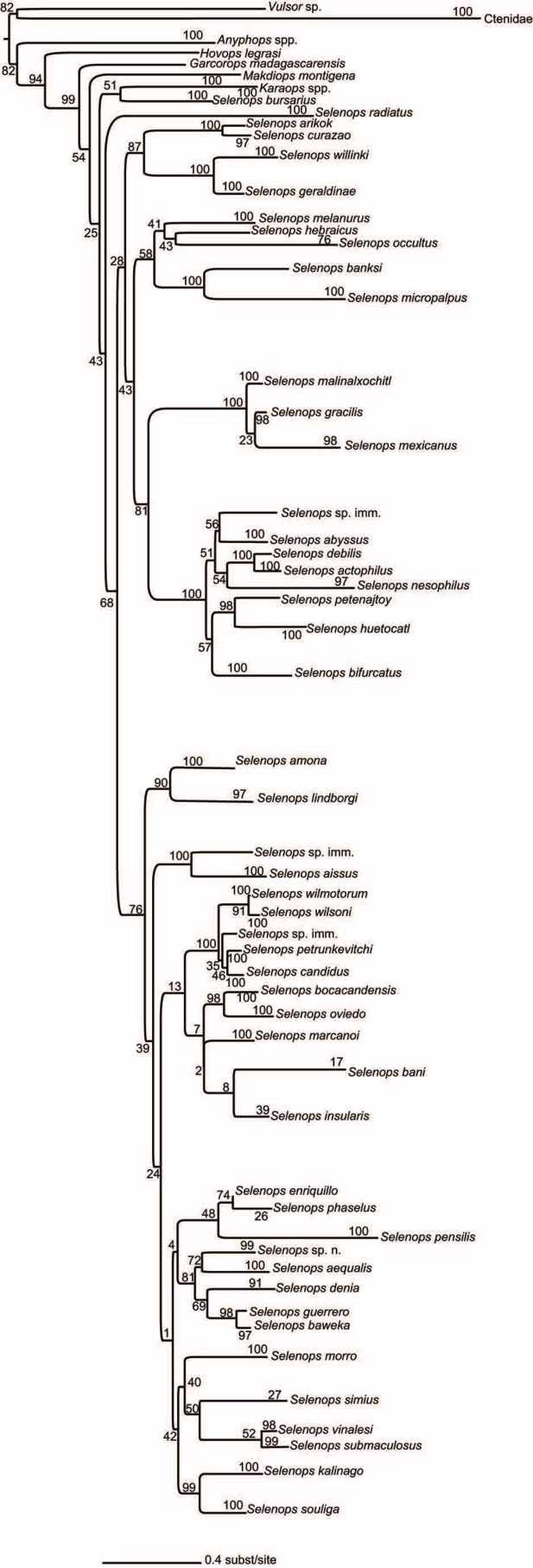


**S15 Table.** **Sequences for *Selenops* analyses.** GenBank accession numbers for *Selenops* spiders and outgroups newly generated for this study. A hyphen (-) indicates that the GenBank numbers can be found in [21] or were not obtained. An asterisk indicates the sequences used in the BEAST 2 and BioGeoBEARS analyses. Distributions considered for each species used in the BioGeoBEARS analyses are provided. (SA=South America; CA=Central America; NA=North America; GA=Greater Antilles; Ja=Jamaica; SLA=Southern Lesser Antilles; NLA=Northern Lesser Antilles; BA=Bahamas and Turks and Caicos Islands; FL=Florida).

| Species | 16S-ND1 | COI | H3 |
| --- | --- | --- | --- |
| *Anyphops* sp*.* sel_996 | - | MN655775 | - |
| *Anyphops barnardi* sel_547 | MN655640 | - | MN655565 |
| *Anyphops barnardi* sel_548 | - | - | MN655566 |
| *Anyphops parvulus* sel_549 | - | - | MN655567 |
| *Anyphops stauntoni* sel_551 | MN655641 | - | - |
| *Hovops legrasi* sel_275 | - | - | MN655568 |
| *Makdiops montigena* sel_853 | MN655642 | - | - |
| *Makdiops montigena* sel_985 | MN655642 | MN655699 | - |
| *Selenops abyssus* sel_1004* ^(NA)^ | MN655689 | MN655750 | - |
| *Selenops abyssus* sel_1006 | MN655690 | MN655749 | - |
| *Selenops abyssus* sel_1012 | - | MN655751 | - |
| *Selenops actophilus* sel_851* ^(NA)^ | MN655691 | MN655752 | - |
| *Selenops aequalis* 00000391A | - | MN655700 | MN655569 |
| *Selenops aequalis* 00784600* ^(GA)^ | MN655665 | MN655723 | MN655571 |
| *Selenops aequalis* 00787562 | MN655666 | MN655741 | MN655570 |
| *Selenops sp.* | MN655683 |  | MN655603 |
| *Selenops aissus* sel_313 | - | MN655753 | - |
| *Selenops aissus* sel_315* ^(GA, FL, BA)^ | - | MN655754 | - |
| *Selenops aissus* sel_320 | - | - | MN655602 |
| *Selenops aissus* sel_325 | MN655684 | - | - |
| *Selenops aissus* sel_326 | - | MN655755 | - |
| *Selenops aissus* sel_331 | - | MN655756 | - |
| *Selenops aissus* sel_336 | MN655685 | - | - |
| *Selenops aissus* sel_338 | MN655686 | MN655757 | - |
| *Selenops amona* sel _847* ^(GA)^ | - | - | - |
| *Selenops arikok* sel_068* ^(SA)^ | - | - | - |
| *Selenops bani* sel_189* ^(GA)^ | MN655664 | - | - |
| *Selenops bani* sel_190 | - | MN655758 | MN655580 |
| *Selenops banksi* sel_1001* ^(SA)^ | - | - | - |
| *Selenops baweka* sel_344* ^(TC)^ | - | - | - |
| *Selenops bifurcatus* sel_882* ^(CA)^ | - | - | - |
| *Selenops bocacanadensis* 00782758 | MN655673 | MN655712 | MN655605 |
| *Selenops bocacanadensis* 00782760 | MN655672 | MN655713 | MN655606 |
| *Selenops bocacanadensis* 00782782 | MN655671 | MN655717 | MN655607 |
| *Selenops bocacanadensis* sel_166* ^(GA)^ | - | - | - |
| *Selenops bocancandensis* 00782740 | MN655670 | MN655711 | MN655604 |
| *Selenops candidus* sel_360* ^(JA)^ | - | - | - |
| *Selenops curazao* sel_047* ^(SA)^ | - | - | - |
| *Selenops debilis* sel_270* ^(NA)^ | - | MN655759 | - |
| *Selenops denia* sel_568 | - | MN655760 | - |
| *Selenops denia* sel_639 | - | - | MN655623 |
| *Selenops denia* sel_641* ^(GA)^ | - | - | - |
| *Selenops denia* sel_643 | MN655667 | - | MN655578 |
| *Selenops denia* sel_646 | MN655668 | - | MN655579 |
| *Selenops enriquillo* sel_182* ^(GA)^ | MN655658 | - | - |
| *Selenops geraldinae* sel_228* ^(SA)^ | - | - | - |
| *Selenops gracilis* sel_1014 | - | MN655761 | MN655630 |
| *Selenops gracilis* sel_1015* ^(NA)^ | - | MN655762 | MN655631 |
| *Selenops guerrero* sel_598* ^(GA)^ | - | - | - |
| *Selenops hebraicus* sel_850* ^(SA)^ | - | - | - |
| *Selenops huetocatl* sel_029 | - | MN655763 | - |
| *Selenops huetocatl* sel_030* ^(NA)^ | - | - | - |
| *Selenops huetocatl* sel_043 | - | - | MN655632 |
| *Selenops huetocatl* sel_045 | - | - | MN655633 |
| *Selenops insularis* 00782766 | - | MN655714 | MN655608 |
| *Selenops insularis* 00782767 | - | MN655715 | MN655612 |
| *Selenops insularis* 00782770 | - | MN655716 | MN655609 |
| *Selenops insularis* 00782871 | - | MN655719 | MN655611 |
| *Selenops insularis* 00784557 | - | MN655722 | MN655613 |
| *Selenops insularis* 00787833 | MN655675 | MN655746 | MN655614 |
| *Selenops insularis* 00787834 | - | MN655747 | MN655610 |
| *Selenops insularis* 00787836 | MN655674 | MN655748 | MN655615 |
| *Selenops insularis* sel_010* ^(GA)^ | - | - | - |
| *Selenops kalinago* sel_760* ^(NLA)^ | - | - | - |
| *Selenops lindborgi* sel_081* ^(GA, NLA, BA)^ | - | - | - |
| *Selenops lindborgi* sel_146 | - | MN655764 | - |
| *Selenops malinalxochitl* sel_1002* ^(NA)^ | - | - | MN655629 |
| *Selenops marcanoi* sel_154* ^(GA)^ | - | - | - |
| *Selenops melanurus* sel_279* ^(SA)^ | MN655687 | - | - |
| *Selenops melanurus* sel_280 | MN655688 | - | - |
| *Selenops* *melanurus* sel_283* ^(SA)^ | - | - | - |
| *Selenops* *sp.* sel_284 | MN655692 | - | - |
| *Selenops mexicanus* sel_977* ^(SA, CA, NA)^ | - | - | - |
| *Selenops micropalpus* 00001886A | MN655693 | MN655707 | MN655624 |
| *Selenops micropalpus* 00001920A | - | MN655708 | MN655625 |
| *Selenops micropalpus* 00001926A | MN655695 | MN655709 | MN655626 |
| *Selenops micropalpus* 00002239A | MN655694 | - | MN655627 |
| *Selenops micropalpus* 00002257A | MN655696 | MN655705 | MN655628 |
| *Selenops micropalpus* sel_090* ^(SLA)^ | - | - | - |
| *Selenops morro* sel_580* ^(GA)^ | - | - | - |
| *Selenops nesophilus* sel_021 | - | MN655765 | MN655636 |
| *Selenops nesophilus* sel_210* ^(NA)^ | - | MN655766 | - |
| *Selenops nesophilus* sel_212 | - | MN655767 | MN655637 |
| *Selenops nesophilus* sel_214 | - | - | MN655638 |
| *Selenops occultus* sel_995 | - | MN655778 | - |
| *Selenops oviedo* sel_624* ^(GA)^ | - | - | - |
| *Selenops pensilis* 00784714 | MN655654 | MN655698 | MN655590 |
| *Selenops pensilis* 00784885 | MN655656 | MN655728 | MN655587 |
| *Selenops pensilis* 00785274 | MN655655 | MN655731 | MN655588 |
| *Selenops pensilis* sel_168 | MN655657 | - | MN655591 |
| *Selenops pensilis* sel_528* ^(GA)^ | - | - | MN655589 |
| *Selenops petenajtoy* sel_865 ^(NA)^ | - | MN655768 | MN655634 |
| *Selenops petenajtoy* sel_866 | - | MN655769 | - |
| *Selenops petenajtoy* sel_867 | - | MN655770 | MN655635 |
| *Selenops petrunkevitchi* 00003309A | - | MN655710 | MN655592 |
| *Selenops petrunkevitchi* sel_368* ^(JA)^ | - | - | - |
| *Selenops phaselus* sel_160* ^(GA)^ | MN655677 | - | - |
| *Selenops phaselus* sel_215 | - | MN655771 | - |
| *Selenops phaselus* sel_562 | - | - | - |
| *Selenops phaselus* sel_565 | MN655679 | - | - |
| *Selenops phaselus* sel_585 | MN655680 | - | MN655620 |
| *Selenops phaselus* sel_632 | MN655678 | MN655772 | - |
| *Selenops phaselus* sel_650 | MN655681 | - | - |
| *Selenops phaselus* sel_651 | - | MN655773 | MN655621 |
| *Selenops phaselus* sel_653 | - | MN655774 | MN655622 |
| *Selenops radiatus* sel_997 | - | MN655776 | - |
| *Selenops simius* 00785225 | MN655659 | - | MN655618 |
| *Selenops simius* 00787702 | MN655660 | MN655742 | - |
| *Selenops simius* 00787703 | MN655662 | - | - |
| *Selenops simius* 00787710 | - | MN655743 | MN655616 |
| *Selenops simius* 00787713 | MN655661 | MN655744 | MN655617 |
| *Selenops simius* 00787714 | MN655663 | MN655745 | MN655619 |
| *Selenops simius* sel_022* ^(GA, BA)^ | - | - | - |
| *Selenops souliga* 00001271A | - | MN655702 | MN655577 |
| *Selenops souliga* 00001065A | - | MN655701 | - |
| *Selenops souliga* 00001268A | - | MN655697 | MN655574 |
| *Selenops souliga* 00001318A | MN655643 | MN655703 | - |
| *Selenops souliga* 00001401A | MN655646 | MN655704 | MN655572 |
| *Selenops souliga* 00001495A | MN655649 | MN655706 | - |
| *Selenops souliga* 00788047 | MN655644 | MN655779 | MN655576 |
| *Selenops souliga* 00788073 | MN655645 | MN655780 | MN655575 |
| *Selenops souliga* sel_1021 | MN655648 | MN655777 | - |
| *Selenops souliga* sel_1022 | MN655647 | - | MN655573 |
| *Selenops souliga* sel_116* ^(NLA)^ | - | - | - |
| *Selenops* sp. cf. *aissus* (imm.) 00783412* ^(GA)^ | MN655682 | MN655721 | MN655601 |
| *Selenops* sp. maybe *S. nigromaculatus* or *S. makimaki* (imm.) sel_1007* ^(NA)^ | - | - | - |
| *Selenops caonabo* 00782859 | MN655669 | MN655718 | - |
| *Selenops* *caonabo* sel_546* | - | - | - |
| *Selenops* sp. sel_285 | MN655676 | - | - |
| *Selenops submaculosus* 00782884 | MN655650 | MN655720 | MN655598 |
| *Selenops submaculosus* 00784744 | - | MN655725 | MN655594 |
| *Selenops submaculosus* 00785204 | - | MN655730 | MN655593 |
| *Selenops submaculosus* 00787294 | - | MN655732 | MN655597 |
| *Selenops submaculosus* 00787405 | - | MN655733 | MN655599 |
| *Selenops submaculosus* 00787423 | MN655652 | MN655734 | MN655596 |
| *Selenops submaculosus* 00787480 | - | MN655729 | MN655595 |
| *Selenops submaculosus* 00787493 | - | MN655735 | - |
| *Selenops submaculosus* 00787494 | - | MN655736 | MN655600 |
| *Selenops submaculosus* 00787495 | MN655651 | MN655737 | - |
| *Selenops submaculosus* sel_301* ^(GA, FL, BA)^ | - | - | - |
| *Selenops submaculosus* 00787521 | - | MN655738 | MN655583 |
| *Selenops vinalesi* 00784745 | MN655653 | MN655726 | MN655582 |
| *Selenops vinalesi* 00784871 | - | MN655727 | MN655584 |
| *Selenops vinalesi* 00787534 | - | MN655739 | MN655586 |
| *Selenops vinalesi* 00787546 | - | MN655740 | MN655585 |
| *Selenops vinalesi* 00784645 |  | MN655724 | MN655581 |
| *Selenops vinalesi* sel_276* ^(GA)^ | - | - | - |
| *Selenops willinki* sel_261* ^(SA)^ | - | - | - |
| *Selenops wilmotourm* sel_376* ^(JA)^ | - | - | - |
| *Selenops wilsoni* sel_381* ^(JA)^ | - | - | - |

**S16 Table.** **Primers and PCR protocols used for amplification of DNA from *Selenops* species.** Certain specimens required different primers to amplify the specific fragments; the details are available from the authors. Some samples required amplification in two pieces, hence the extra primers. Any additional info is available from the authors.

| Primer | Sequence | Reference | PCR protocol |
| --- | --- | --- | --- |
| 16SA – 12261mod  Selenops new 16SR  SPID ND1 13398  16SB - modF | 5’ -CGMCTGTTTAMCAAAAACAT – 3’  5’ – TTCTAATGCATTTWTCTGCCAA – 3’  5’ – TCRTAAGAAATTATTTGAGC - 3’  5’ – GATYTGAGTTCARACCGG – 3’ | This study  This study  [213]  This study | 92˚C 30s  92˚C 30s  44˚C 45s, increase temp after cycle 1 by 0.2˚C every cycle  72˚C 1min 30s  30X  72˚C 5 min  10˚C hold |
| LCO11490 mod  C1N2568 mod  Jerry mod R  Selenops new CO1 F | 5’ - ATTCWACWAATCATAARGATATTGG-3’  5’ – GCTACWACATAATAMGTATCATG – 3’  5’ – CCAAAAAAYCAAAAYAAATGTTG -3’  5’ – CWGATCGWAATTTAATACTTCDTTTT –3’ | This study | 95˚C 2 min  95˚C 30s  47˚C 45s  72˚C 1min 30s  35X  72˚C 5 min  8˚C hold |
| H3aF  H3aR | 5′-ATGGCTCGTACCAAGCAGACVGC-3′  5′-ATATCCTTRGGCATRATRGTGAC-3′ | [214] | 95˚C 2 min  95˚C 30s  53˚C 45s  72˚C 1min  35X  72˚C 10 min  8˚C hold |

25 μL rxns

16S-ND1 and COI - 11.3 μL ddH_2_O, 5 μL Promega GoTaq 5x Buffer, 0.5 μL Promega dNTPs, 3 μL Promega MgCl_2_, 2 μL BSA, 0.2 μL Promega GoTaq, 1 μL ea. primer

H3 – 13.3 μL ddH_2_O, 5 μL Promega GoTaq 5x Buffer, 0.5 μL Promega dNTPs, 2 μL Promega MgCl_2_, 1 μL BSA, 0.2 μL Promega GoTaq, 1 μL ea. primer

Cycle Sequence – 10 μL rxns – 6.45 μL ddH_2_O, 1.5 μL Buffer, 0.3 μL primer, 0.75 μL Big Dye

For faint bands: 20 μL rxns – 7.4 μL ddH_2_O, 3 μL Buffer, 0.6 μL primer, 4μL Big Dye

96˚C 1min; (96˚C – 10s; 50˚C – 5s; 60˚C – 1min 15s; 15X); (96˚C – 10s; 50˚C – 5s; 60˚C – 1min 30s; 5X; 96˚C – 10s; 50˚C – 5s; 60˚C – 2 min; 5X); 8˚C hold
